# Supplementary figures and images for: Identical anthropometric characteristics of impaired fasting glucose combined with impaired glucose tolerance and newly diagnosed type 2 diabetes: anthropometric indicators to predict hyperglycaemia in a community-based prospective cohort study in southwest China
Source: BMJ Open. 2018 May 9;8(5):e019735. doi: 10.1136/bmjopen-2017-019735 (PMC5942465; doi:10.1136/bmjopen-2017-019735)

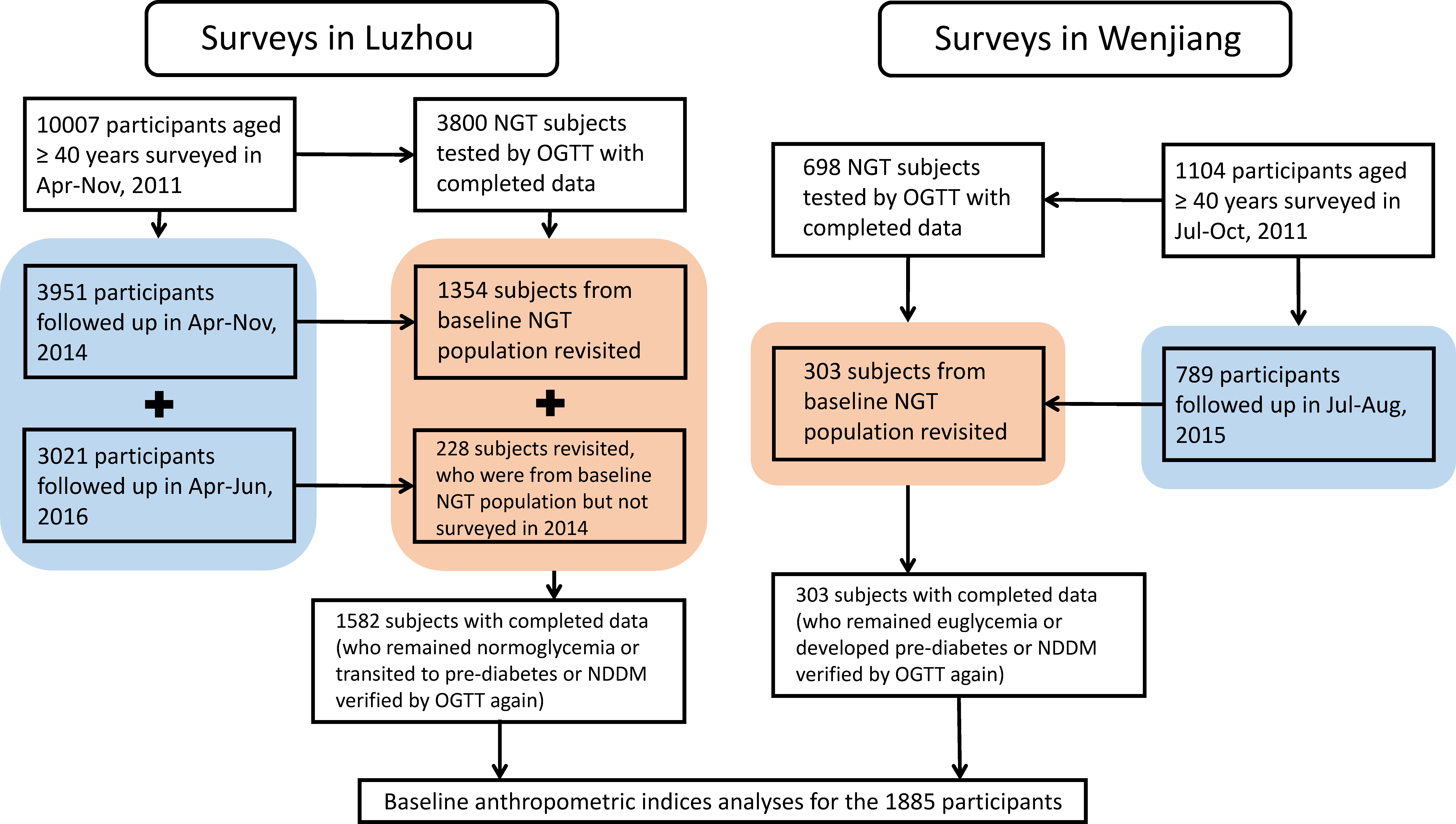

Supplement: Supplementary data [file bmjopen-2017-019735supp001.jpg]
