# Supplementary material for: Identical anthropometric characteristics of impaired fasting glucose combined with impaired glucose tolerance and newly diagnosed type 2 diabetes: anthropometric indicators to predict hyperglycaemia in a community-based prospective cohort study in southwest China
Source: BMJ Open. 2018 May 9;8(5):e019735. doi: 10.1136/bmjopen-2017-019735 (PMC5942465; doi:10.1136/bmjopen-2017-019735)
Supplement: Supplementary data [file bmjopen-2017-019735supp002.pdf]

Supplemental Table 1. Baseline characteristics of all the participants screened in Luzhou and Wenjiang

	Luzhou baseline survey			*P*	Wenjiang baseline survey			*P*
	Total (n = 1582)	Men (n = 495)	Women (n = 1087)		Total (n = 303)	Men (n = 154)	Women (n = 149)	
Age (year)	57 (50—63)	60 (54—66)	56 (49—61)	0.000	47 (43—54)	47 (43—54)	46 (42—52)	0.161
Female (N/n%)	1087 (68.71%)	—	—		149 (49.17%)	—	—	
Height (cm)	157.00 (152.40—163.00)	165.00 (160.50—169.00)	154.55 (151.00—158.20)	0.180	161.28 ±7.60	166.46 ±5.27	155.56 ±5.32	0.000
Weight (kg)	59.00 (53.00—65.30)	65.00 (58.30—72.00)	56.50 (51.20—62.50)	0.000	61.88 ±11.36	67.66 ±8.84	55.49 ±10.38	0.000
Hip circumference (cm)	94.00 (89.20—99.00)	95.00 (90.00—100.00)	94.00 (89.00—98.20)	0.655	93.47 ±6.04	95.01 ±5.43	91.76 ±6.24	0.000
SBP (mmHg)	120.67 (108.67—135.67)	126.00 (113.83—140.17)	119.00 (107.00—133.37)	0.000	114.90 ±14.27	118.20 ±14.02	111.26 ±13.69	0.000
DBP (mmHg)	75.33 (69.00—82.67)	79.00 (71.67—88.17)	74.00 (68.00—80.67)	0.000	78.40 ±16.26	81.15 ±10.84	75.36 ±20.27	0.001
FPG (mmol/L)	5.14 (4.93—5.34)	5.15 (4.96—5.38)	5.13 (4.92—5.32)	0.011	4.90 (4.60—5.10)	4.90 (4.70—5.20)	4.80 (4.60—5.10)	0.286
2hPG (mmol/L)	6.32 (5.57—7.00)	6.32 (5.57—6.98)	6.32 (5.57—7.01)	0.777	6.00 (5.00—6.70)	5.90 (5.03—5.78)	6.00 (5.00—6.80)	0.541
HbA1c (%)	5.70 (5.40—5.90)	5.70 (5.50—5.95)	5.70 (5.40—5.90)	0.069	5.48 ±0.42	5.51 ±0.38	5.45 ±0.45	0.228
TG (mmol/L)	1.33 ±0.94	1.29 ±0.84	1.34 ±0.98	0.388	1.10 (0.80—1.80)	1.50 (0.90—2.18)	0.90 (0.70—1.50)	0.000
TC (mmol/L)	4.44 (3.75—5.18)	4.32 (3.63—5.12)	4.50 (3.82—5.19)	0.017	4.53 ±0.83	4.60 ±0.82	4.45 ±0.83	0.178
HDL-c (mmol/L)	1.28 (1.06—1.52)	1.26 (1.03—1.52)	1.29 (1.08—1.52)	0.107	1.59 ±0.39	1.47 ±0.32	1.71 ±0.41	0.000
LDL-c (mmol/L)	2.52 ±0.77	2.47 ±0.74	2.54 ±0.79	0.078	2.86 ±0.75	2.92 ±0.70	2.79 ±0.80	0.252
WHtR (cm/cm)	0.52 (0.48—0.56)	0.53 (0.49—0.56)	0.52 (0.48—0.56)	0.900	0.49 (0.45—0.53)	0.50 (0.47—0.53)	0.47 (0.44—0.51)	0.000
BMI (kg/m^2)	23.74 (21.61—26.00)	24.04 (21.81—26.14)	23.68 (21.51—25.92)	0.462	23.49 (21.64—25.59)	24.40 (22.33—26.03)	22.44 (21.10—24.24)	0.000
Waist circumference (cm)	82.00 (76.00—89.00)	87.00 (80.00—92.45)	80.00 (75.00—87.10)	0.201	79.00 (72.00—86.00)	84.00 (79.00—89.00)	73.00 (69.75—78.00)	0.000
WHR (cm/cm)	0.86 (0.80—0.91)	0.89 (0.83—0.94)	0.85 (0.79—0.90)	0.476	0.86 (0.80—0.91)	0.83 (0.78—0.89)	0.87 (0.81—0.92)	0.256
Outcomes at follow-up: N/total (%)								
NGT	757 (47.85%)	203 (41.01%)	554 (50.97%)	—	215 (70.96%)	103 (66.88%)	112 (75.17%)	—
Isolated IFG	131 (8.28%)	51 (10.30%)	80 (7.34%)	—	28 (9.24%)	12 (7.79%)	16 (10.74%)	—
Isolated IGT	304 (19.22%)	103 (20.81%)	201 (18.49%)	—	38 (12.54%)	24 (15.58%)	14 (9.40%)	—
IFG+IGT	137 (8.66%)	46 (9.29%）	91 (8.37%)	—	15 (4.95%)	11 (7.14%)	4 (2.68%)	—

NDDM	253 (15.99%)	92 (18.59%)	161 (14.81%)	—	7 (2.31%)	4 (2.61%)	3 (2.01%)	—

SBP, systolic blood pressure; DBP, diastolic blood pressure; FPG, fasting plasma glucose; 2hPG, 2 hour plasma glucose (after oral glucose tolerance test); TG, triglyceride; TC, total cholesterol; HDL-c, high-density lipoprotein cholesterol; LDL-c, low-density lipoprotein cholesterol; WHtR, waist-to-height ratio; BMI, body mass index; WHR, waist-to-hip ratio; NGT, normal glucose tolerance; IFG, impaired fasting glucose; IGT, impaired glucose tolerance; IFG+IGT, IFG combined with IGT; NDDM, newly-diagnosed diabetes mellitus.

Data are expressed as mean ± SD, or median (interquartile range), or N (%).

Mann-Whitney U analysis was used for DBP and BMI in Luzhou, TG and HDL-c in Wenjiang; one-way ANOVA analysis was used for the rest measurements in two surveys.

P value of men versus women.
