# Supplementary material for: Identical anthropometric characteristics of impaired fasting glucose combined with impaired glucose tolerance and newly diagnosed type 2 diabetes: anthropometric indicators to predict hyperglycaemia in a community-based prospective cohort study in southwest China
Source: BMJ Open. 2018 May 9;8(5):e019735. doi: 10.1136/bmjopen-2017-019735 (PMC5942465; doi:10.1136/bmjopen-2017-019735)
Supplement: Author's manuscript [file bmjopen-2017-019735.draft_revisions.pdf]

BMJ Open is committed to open peer review. As part of this commitment we make the peer review history of every article we publish publicly available.

When an article is published we post the peer reviewers' comments and the authors' responses online. We also post the versions of the paper that were used during peer review. These are the versions that the peer review comments apply to.

The versions of the paper that follow are the versions that were submitted during the peer review process. They are not the versions of record or the final published versions. They should not be cited or distributed as the published version of this manuscript.

BMJ Open is an open access journal and the full, final, typeset and author-corrected version of record of the manuscript is available on our site with no access controls, subscription charges or pay-per-view fees (http://bmjopen.bmj.com).

If you have any questions on BMJ Open's open peer review process please email
info.bmjopen@bmj.com

BMJ Open

Identical anthropometry characteristics of impaired fasting glucose combined with impaired glucose tolerance and newly-diagnosed type 2 diabetes: anthropometric indicators for hyperglycemia prediction in a community-based prospective cohort study

Journal:	*BMJ Open*
Manuscript ID	bmjopen-2017-019735
Article Type:	Research
Date Submitted by the Author:	23-Sep-2017

Complete List of Authors:	Zhang, Fang; Sichuan University West China Hospital, Division of Endocrinology and Metabolism<br>Wan, Qin; Hospital affiliated to Southwest Medical University, Division of Endocrinology and Metabolism<br>Cao, Hongyi; The Fifth People's Hospital of Chengdu, Chengdu<br>Tang, Lizhi; Sichuan University West China Hospital, Division of Endocrinology and Metabolism<br>Li, Daigang; Chengdu Yinchao Community Hospital<br>Yan, Zhe; Sichuan University West China Hospital<br>Li, Jing; Sichuan University West China Hospital<br>Yang, Qiu; The Fifth People's Hospital of Chengdu<br>Lü, Qingguo; Sichuan University West China Hospital<br>Zhang, Yuwei; Sichuan University West China Hospital<br>Tong, Nanwei; Sichuan University West China Hospital, Division of Endocrinology and Metabolism

**Primary Subject Heading**:	Diabetes and endocrinology
Secondary Subject Heading:	Epidemiology, Diabetes and endocrinology
Keywords:	anthropometric index, impaired fasting glucose combined with impaired glucose tolerance, newly-diagnosed diabetes mellitus, pre-diabetes, waist-to-height ratio

SCHOLARONE™ Manuscripts

Title:

Identical anthropometry characteristics of impaired fasting glucose combined with impaired glucose tolerance and newly-diagnosed type 2 diabetes: anthropometric indicators for hyperglycemia prediction in a community-based prospective cohort study

Authors:

Fang Zhang 1*, Qin Wan 2*, Hongyi Cao 3*, Lizhi Tang 1, Daigang Li 4, Zhe Yan 1, Jing Li 1, Qiu Yang 3, QingguoLü 1, Yuwei Zhang 1, Nanwei Tong 1△

*These authors contributed equally to this paper.

△Corresponding author

Authors' affiliations:

1. Division of Endocrinology and Metabolism, West China Hospital, Sichuan University, Chengdu, Sichuan, China
2. Division of Endocrinology and Metabolism, Hospital affiliated to Southwest Medical University, Luzhou, Sichuan, China
3. Division of Endocrinology and Metabolism, The Fifth People's Hospital of Chengdu, Chengdu, Sichuan, China
4. Chengdu Yinchao Community Hospital, Chengdu, Sichuan, China

Correspondences:

Address correspondence and reprint requests to Nanwei Tong, MD, Division of Endocrinology and Metabolism, West China Hospital, Sichuan University, 37 Guoxuexiang, Chengdu, Sichuan 610041, China. Telephone number: 00+86-18980601196. Fax number: 00+86-28-85423459. E-mail: tongnw@scu.edu.cn.

For peer review only

Abstract

Objective: To assess the anthropometry characteristics in euglycemic individuals who developed hyperglycemia subsequently and to evaluate the validity for pre-diabetes and diabetes identification by anthropometric indices in Southwest China.

Design: Community-based prospective cohort study.

Participants and setting: Pre-diabetes-free and diabetes-free residents (n=1885) at entry from six communities were enrolled in this study.

Main outcome measures: Pre-diabetes or diabetes incidence.

Methods: In this community-based prospective cohort study, the waist-to-height ratio (WHtR), body mass index (BMI), waist circumference (WC) and waist-to-hip ratio (WHR) of all participants were measured at each survey in Sichuan. The 75 g glucose oral glucose tolerance test was conducted both at baseline and follow-up surveys.

Results: During a median of 3.00 (2.92-4.17) years follow-up, the cumulative rates of incident isolated impaired fasting glucose (IFG), isolated impaired glucose tolerance (IGT), IFG combined with IGT (IFG+IGT) and newly-diagnosed diabetes mellitus (NDDM) were 8.44%, 18.14%, 8.06% and 13.79% among all the participants. WHtR, BMI, WC and WHR were significantly different among subjects who progressed to isolated IFG/IGT, IFG+IGT or NDDM subsequently ($P < 0.05$). Of note, the anthropometry characteristics of IFG+IGT subjects were similar to that of NDDM population ($P > 0.005$). All the anthropometric indices at entry were valuable to predict future pre-diabetes and NDDM incidences ($P < 0.05$). The optimal cut-off points of the four measurements were obtained to predict hyperglycemia, with WHtR of the value around 0.52 performing best to identify isolated IFG/IGT, IFG+IGT and NDDM.

Conclusions: Anthropometric measures, especially WHtR, could predict hyperglycemia incidences in advance for 3 years. Differed from isolated IFG/IGT, the individuals who developed IFG+IGT had identical anthropometric profiles to those who transited to NDDM.

Key words: anthropometric index, impaired fasting glucose combined with impaired glucose tolerance, newly-diagnosed diabetes mellitus, pre-diabetes, waist-to-height ratio

For peer review only

Strengths and limitations of this study

1.This study not only illustrated and compared the anthropometric characteristics of participants who subsequently progressed to diverse hyperglycemic conditions, but also revealed the variation tendencies of waist-to-height ratio (WHtR), body mass index (BMI), waist circumference (WC) and waist-to-hip ratio (WHR) in the natural transition from normal glucose tolerance (NGT) to pre-diabetes, and to overt newly-diagnosed diabetes mellitus (NDDM) in advance for 3 years.

2. WHtR performed best to identify future hyperglycemia incidence.

3. The follow-up duration of a median 3.00 years was relatively short.

4. The overall re-visiting ratio was low (41.91%).

5. The sample size was limited to obtain the anthropometric cut-off values in each hyperglycemic state by gender.

For peer review only

Introduction

With the rapidly growing diabetes occurrences, it is now reaching epidemic proportions in China. The overall prevalence of diabetes and pre-diabetes were estimated to be 11.6% and 50.1% in the Chinese adult population in 2010 [1]. In 2007-2008, another national cross-sectional study in China indicated that the prevalence of isolated impaired fasting glucose (IFG), isolated impaired glucose tolerance (IGT), and IFG combined with IGT (IFG+IGT) were 3.2%, 11.0% and 1.9% in men, while 2.2%, 10.9% and 1.7% among women [2]. Isolated IFG, isolated IGT and IFG+IGT were three different status of pre-diabetes, reflecting the natural transition from normoglycemia to type 2 diabetes (T2D). Approximately 75-80% diabetes patients develop cardiovascular disease (CVD) ultimately and pre-diabetes is also verified to be at increased risk of heart attacks and strokes [3-5]. It was estimated that from 2005 to 2015, diabetes and its relative CVD would cause a total of US$ 557.7 billion loss in China [6].

Overall and central adiposities are closely linked to hyperglycemia. Body mass index (BMI) is a measurement correlated with overall fat, while waist circumference (WC), waist-to-height ratio (WHtR) and waist-to-hip ratio (WHR) are three central obesity indicators. The four anthropometric indices are globally used to assess the risk of having current or future diabetes [7-9].

Actions to address pre-diabetes are critical for preventing diabetes. Early recognition and prompt intervention could release the stress from the whole society. Anthropometry is an affordable and practical screening tool for hyperglycemia both in advanced and impoverished areas of China. In this community-based prospective cohort study, we aimed to examine whether the baseline anthropometric indices could predict the future pre-diabetes and diabetes incidences with optimal cut-off values. The baseline anthropometric characteristics of euglycemic subjects, who developed isolated IFG, isolated IGT, IFG+IGT and newly-diagnosed diabetes mellitus (NDDM) during follow-up, were displayed. Additionally, the potential similarity and distinction between any two hyperglycemic disorders were detected.

Study design and methods

Study population

The present study included two surveys conducted in Luzhou City and Wenjiang area of Chengdu City, respectively. The Luzhou survey was one part of the REACTION research, which is a multicenter prospective observational study containing 25 communities in mainland China [10, 11]. A total of 10007 regular residents, aged of 40-89 years, were randomly recruited to participate in our investigation from five communities of Luzhou in 2011. Subjects with history of diabetes, incident diabetes or pre-diabetes verified by oral glucose tolerance test (OGTT), missing values of any measurement, or of other exclusive conditions (see below) were excluded. There were only 3800 individuals with euglycemia remained as our baseline population. Among them, only 1354 participants revisited in 2014 and had completed data. Furthermore, in 2016, 228 residents from baseline normoglycemia population but not surveyed in 2014 were followed up. Therefore, a total of 1582 subjects

For peer review only

from Luzhou screening were available for current work.

In the Wenjiang survey, a cohort of 1104 participants aged 40-75 years were randomly recruited from Yinchao community in 2011. According to the same inclusion criteria, 698 euglycemic individuals were considered as the baseline population. Among them, 303 subjects were followed up in 2015 and received completed measurements. Thus, from Luzhou and Wenjiang, a total of 1885 participants were included in our study.

All the included populations are of the Han nationality in China. The flow path of our study design is displayed in Supplemental Figure 1. The other exclusion criteria were infection, pregnancy, malignant tumor, acute cardiovascular accidents, serious trauma, liver or renal dysfunction, and long history of glucocorticoids use. The research was conducted in accordance with the principles of the Declaration of Helsinki II. All protocols used in this work were approved either by the Medical Ethics Committee of Hospital affiliated to Southwest Medical University in Luzhou, or by the Committee on Human Research at the Fifth People's Hospital of Chengdu in Wenjiang. Each participant provided written informed consent.

Diagnosis of diabetes and pre-diabetes

The hyperglycemic disorder definitions are in accordance with American Diabetes Association recommendation by OGTT in 2011 [12]. Normal glycemia tolerance (NGT) is defined as fasting plasma glucose (FPG) < 5.6mmol/L and 2-hour plasma glucose (2hPG) < 7.8 mmol/L. Isolated IFG means 5.6 mmol/L ≤ FPG < 7.0 mmol/L and 2hPG < 7.8 mmol/L, while isolated IGT means FPG < 5.6mmol/L and 7.8 mmol/L ≤ 2hPG < 11.1 mmol/L. IFG+IGT equals to 5.6 mmol/L ≤ FPG < 7.0 mmol/L and 7.8 mmol/L ≤ 2hPG < 11.1 mmol/L. Diabetes is defined as FPG ≥ 7.0 mmol/L and/or 2hPG ≥ 11.1 mmol/L.

Anthropometric measurements

Anthropometric measurements including body weight, height, WC and hip circumference were recorded by trained examiners. All participants were measured when wearing light clothing without foot-wearing after 10-12 hours overnight fasting in the morning. Measurements were conducted by using calibrated weighing scale, standard steel strip stadiometer and tape measure. The results were recorded to the nearest 0.1 kg and 0.1 cm. WC was obtained at the midway between the costal border and iliac crest at the end of exhalation. Hip circumference was taken around the widest portion of the buttocks. BMI was calculated as body weight (in kg) divided by squared height (in m^2), WHtR as WC (in cm) divided by height (in cm), and WHR as WC (in cm) divided by hip circumference (in cm).

Lifestyle variables and biological evaluation

Trained investigators collected lifestyle information on demographic characteristics, current smoking status, physical activity situation, medications, personal and family disease histories through a standard questionnaire and face-to-face interviews. Blood pressure (BP) was measured three times for each participant by an electronic sphygmomanometer (OMRON, HEM-7220, Liaoning, China) with 5 min intervals after at least 10 min rest, whose average

For peer review only

value was taken.

All participants accepted an OGTT screening. After 10-12 hours overnight fasting, venous blood specimens were drawn both before and 2 hours after they drank 300 ml water containing 75 g anhydrous glucose within 5 min. FPG and 2hPG concentrations were measured within 24 hours by hexokinase method (Hitachi 7600 automatic biochemical analyzer, Hitachi Ltd., Tokyo, Japan). Fasting total cholesterol (TC), triglyceride (TG), high-density lipoprotein cholesterol (HDL-c) and low-density lipoprotein cholesterol (LDL-c) were also obtained within 24 hours by oxidase colorimetric method (same Hitachi 7600 analyzer as above). HbA1c was measured via the high performance liquid chromatography method (VARIANT™ II TURBO Hemoglobin Testing System, Bio-Rad Laboratories, CA, USA). These samples were stored at -20 □ till analyzed, which were of 3-week storage per batch.

Statistical analysis

Data analyses were carried out by SPSS software version 16.0 (SPSS, Chicago, IL, USA) and MedCalc software version 15.2.2 (MedCalc software, Ostend, Belgium). All data were expressed as means ± SD or median (interquartile range) or frequency (%), as appropriate. One-way ANOVA analysis was used for parametric materials, while rank sum test was applied for nonparametric variables. Chi-square test was assessed for constituent ratio comparison. All tests performed were two-sided. Among multiple groups (groups ≥ 3) comparison, the overall P value less than 0.05 was considered as significant. Furthermore, the Bonferroni correction and chi-square segmentation were used for multiple comparison adjustments. The P value within two specific subgroups comparison was significant when less than 0.005. For BMI, WHtR, WC and WHR, the receiver operating characteristic (ROC) curve analyses were applied for comparing their ability to predict incident pre-diabetes and NDDM. The nonparametric approach described by DeLong et al. was used to compare the areas under correlated ROC curves [13]. The predictive cut-off values for hyperglycemia were calculated. COX proportional hazards regression was used to evaluate the association between anthropometric indices and hyperglycemia incidences. Time axis was follow-up until (pre-)diabetes incidence or follow-up termination. Hazard ratio (HR) and 95% confidence interval (CI) were calculated.

Results

Characteristics of all subjects at baseline

A total of 1885 euglycemic subjects (649 males and 1236 females), with median age of 56.00 (48.00-61.00) years old, were recruited in our study in 2011. After a median follow-up of 3.00 (2.92-4.17) years, 159 individuals of them transited to isolated IFG, 342 to isolated IGT, 152 to IFG+IGT, 260 to NDDM, while the rest 972 participants still remained normoglycemia. The incidence rates of pre-diabetes and NDDM were calculated to be 104.9 per 1000 person-years and 41.8 per 1000 person-years. Characteristics of all subjects at baseline in Luzhou and Wenjiang are illustrated in Supplemental Table 1. The baseline general measurements of participants, who developed isolated IFG, isolated IGT, IFG+IGT and NDDM in future, are shown in Table 1.

For peer review only

Anthropometric measures of subjects who subsequently developed diverse hyperglycemic disorders at follow-up and baseline

During the follow-up survey, it was found that the WHtR in NGT group was lower than that in isolated IGT, IFG+IGT and NDDM groups ($P < 0.005$) (Table 2), while it was lower in isolated IFG and isolated IGT populations than that in IFG+IGT and NDDM individuals ($P < 0.005$). Though the p values were 0.009 and 0.006 of BMI in isolated IFG vs. IFG+IGT and isolated IGT vs. IFG+IGT, 0.005 of WHR in isolated IFG/IGT vs. IFG+IGT, respectively, it displayed a trend of the difference between isolated IFG/IGT and IFG+IGT. Conclusively, the values of BMI, WC and WHR in the five glucose metabolic statuses were presented in the variation tendency of NGT < isolated IFG, isolated IGT < IFG+IGT, NDDM. Unlike isolated IFG and isolated IGT, the anthropometric characteristics in IFG+IGT were similar to that in NDDM at follow-up ($P > 0.005$).

To assess whether the anthropometry had already changed before hyperglycemia presenting, we concentrated in its alteration at baseline when all subjects were still of euglycemia. Except for WC, the baseline WHtR, BMI and WHR were substantially disparate among the five glucose metabolic groups ($P < 0.05$) (Table 2). The WHtR values of IFG+IGT and NDDM groups were higher than that of NGT and isolated IFG groups ($P < 0.005$), while isolated IGT populations had smaller WHtR than NDDM patients ($P < 0.005$). The BMI index of NGT group was lower than that of isolated IGT, IFG+IGT and NDDM groups ($P < 0.005$), while isolated IFG people had lower BMI than NDDM subjects ($P < 0.005$). Additionally, NGT individuals were of thinner WHR than NDDM patients ($P < 0.005$). Consistent findings as above at follow-up, it is worthy to note that there was no significant difference of WHtR, BMI or WHR between subsequent IFG+IGT and NDDM at baseline ($P > 0.005$).

Predictive values of baseline anthropometric indices to identify future pre-diabetes and NDDM incidences

For isolated IFG prediction, baseline WHtR, WC and WHR were of significant areas under the curves (AUCs) ($P < 0.05$) (Table 3). WHtR and WC to predict isolated IFG yielded higher value than BMI ($P < 0.05$) (Figure 1A). In isolated IGT population, the AUCs of all the four indices were significant ($P = 0.000$). WHtR received higher predictive value than BMI, WC and WHR ($P < 0.05$), while WC was superior to WHR for predicting isolated IGT ($P < 0.05$) (Figure 1B). For IFG+IGT incidence, the four measurements were valuable predictors ($P = 0.000$), among which WHtR and WC ranked higher than WHR ($P < 0.05$) (Figure 1C). For NDDM identification, the four indices were substantially significant ($P < 0.05$), where WHtR was the best predictor ($P < 0.05$) (Figure 1D). Moreover, the optimal cut-off points for predicting hyperglycemia of the four indices (WC and WHR cut-off values for men and women respectively) were obtained.

Multivariable analysis of baseline anthropometric indices in relation to risk of subsequent pre-diabetes and NDDM

According to COX proportional hazards regression, potential risk factor for developing isolated IFG was increased WC at baseline ($P < 0.05$) (Table 4). Potential risk factors for

transiting to isolated IGT were incremental WHtR, BMI and WC at entry ($P < 0.05$). For both IFG+IGT and NDDM incidences, increased baseline WHtR, BMI, WC and WHR were the risk factors ($P < 0.05$).

Discussion

From this community-based prospective cohort study, we found that: (1) When patients diagnosed overt pre-diabetes and NDDM, the values of WHtR, BMI, WC and WHR were presented as the tendency of NGT < isolated IFG, isolated IGT < IFG+IGT, NDDM. (2) Among the diverse hyperglycemic disorders, noteworthy is that unlike isolated IFG and isolated IGT, no significant difference of baseline WHtR or BMI was found between IFG+IGT and NDDM subjects. (3) WHtR, BMI, WC and WHR could predict subsequent incidences of pre-diabetes and diabetes in advance for 3 years. The greater baseline anthropometric values people were of, the higher risk for developing hyperglycemia they were at. (4) Optimal cut-off values of the four anthropometric measures for identifying pre-diabetes and diabetes were obtained, with WHtR performing best to detect hyperglycemia among all indices.

An Iranian research including 5879 participants who were initially free of hyperglycemia, after 9-year follow-up, 1755 subjects developed pre-diabetes, where isolated IFG had the highest incidence rate among all pre-diabetes phenotypes. They found that among women, compared with BMI, hip and wrist circumferences, WHtR was the only significant anthropometric predictor of pre-diabetes [14]. Lyssenko et al. reported a study of 1190 NGT subjects at baseline in Finland. During a median follow-up of 6 years, 199 progressed to pre-diabetes. Compared with those who remained NGT, the pre-diabetes had substantially higher BMI and WHtR at entry [15]. An increasing number of scholars have realized that the anthropometry is tightly correlated with pre-diabetes incidence, though most of their evidences based on cross-sectional data [16-19].

After reviewing these literatures, we found some points in common: (1) Referred to pre-diabetes, the majority of these studies only involved one or two pre-diabetic phenotypes. Some even mixed them as a whole, generally called “pre-diabetes”. (2) Rare investigators described the respective anthropometry characteristics of various hyperglycemic disorders in their manuscript. We only read one report displaying the anthropometric information in details of all pre-diabetic phenotypes and NDDM together [20]. It was observed that WHtR, BMI, WC and WHR were substantially distinct among NGT, isolated IFG, isolated IGT, IFG+IGT and NDDM subjects. But none of the anthropometric indices were compared within any two hyperglycemic groups. Therefore, the variation tendency of anthropometry in pre-diabetes and NDDM could not be illustrated. Moreover, this study was based on cross-sectional design. To our knowledge, the present work is the first prospective cohort study that not only illustrated the anthropometric characteristics of participants who progressed to diverse hyperglycemic conditions, but also revealed the variation tendencies of WHtR, BMI, WC and WHR in the natural transition from NGT to pre-diabetes, and to overt NDDM.

Isolated IFG and isolated IGT are of heterogeneous pathogenesis, while IFG+IGT manifests

For peer review only

both hepatic and peripheral insulin resistances. Pre-diabetes, as an intermediate hyperglycemia, is a high-risk state for diabetes development. Among the three phenotypes of pre-diabetes, IFG+IGT approximately doubled the rate of diabetes transition compared with subjects with just one of them [21]. In our previous work, it was found that during the progression from NGT to overt T2D, differentiated from isolated IFG and isolated IGT, several biomarkers in IFG+IGT individuals had already presented the similar alteration to those in NDDM population [22-24]. Consistently, in the present study, we observed that participants who developed hyperglycemia in future had higher WHtR, BMI and WHR at entry than those who remained NGT. Among the three pre-diabetic statuses, IFG+IGT subjects were of the highest anthropometric profile at baseline, which manifested no significant difference from that in NDDM group. These findings may imply that though IFG+IGT is a subtype of pre-diabetes, some disorders of its pathophysiology have already deteriorated to the same extent as NDDM does. Pre-diabetes is a reversible condition. Consequently, prompt intervention is needed to avoid or delay its progression, especially for the patients with IFG+IGT.

A prospective study conducted in Pima Indians population found that BMI and WHtR were the best predictors of diabetes in men, while BMI, WHtR, WC and waist-to-thigh ratio were the best predictors in women [25]. Chei et al. published a cohort study of 5617 Japanese participants. Only for women, the significant predictors for T2D were BMI, WC and WHtR [26]. In a multi-ethnic cohort of 1073 non-Hispanic white, Hispanic and African American non-diabetic individuals, their baseline anthropometric information showed that in the non-Hispanic white and Hispanic populations, BMI was most predictive of diabetes, whereas all central obesity indicators ranked higher than overall adiposity measures in African American population [27]. These inconclusive evidences indicated that the validities of those anthropometry measurements for diabetes identification are variable in different ethnicities, genders and regions. Based on our ROC analysis, WHtR showed the highest value for identifying pre-diabetes and overt NDDM, followed by WC, while BMI and WHR were relatively weak predictors. Results from two Western Pacific studies were consistent with our findings [28, 29].

A systematic review proposed that the boundary values of WHtR for diabetes prediction in men and women were 0.52 and 0.53, respectively [30]. In a Chinese community-based prospective cohort study, the optimal cut-offs for diabetes of WHtR, and BMI were 0.51 and 24 for men, while 0.55 and 25 for women [29]. These predictive cut-off values were similar to the data in our study.

Several limitations in our work should be addressed. First, the follow-up duration of a median 3.00 years was relatively short. But we were shocked by the cumulative incidence rates of pre-diabetes and NDDM at 34.64% and 13.79%. The fast-paced life and sedentary lifestyle may contribute mostly to the rapidly growing hyperglycemia. Second, the overall re-visiting ratio was low (41.91%). Phone interview once a year at least and prompt examination propagandizing for visitors may reduce the lost rate. Third, the sample size was limited. On account of this weakness, it was invalid to obtain the anthropometric cut-off values in each

For peer review only

hyperglycemic state by gender. Further studies are needed to acquire specific cut-off points for screening pre-diabetes and NDDM in men and women respectively, especially for the WC and WHR indicators.

In summary, WHtR, BMI, WC and WHR are all predictable to identify pre-diabetes and NDDM incidences in advance for 3 years. Individuals with increased WHtR, BMI, WC and WHR at entry are at higher risk for developing pre-diabetes and T2D. The optimal cut-off points of all the anthropometric measurements to predict hyperglycemia were obtained, with WHtR of the value around 0.52 performing best to identify isolated IFG/IGT, IFG+IGT and NDDM. WHtR and BMI at baseline could illustrate the gradually increased tendency in the natural progression from euglycemia to pre-diabetes, and to overt T2D subsequently. Of note, distinguished from isolated IFG and isolated IGT, the anthropometry characteristics of IFG+IGT subjects were similar to that of NDDM population both at baseline and follow-up.

For peer review only

Contributorship statement

All the authors engaged in the surveys. FZ and NT designed this article. QW, HC, DL and QY acquired and collected data. JL, ZY, QL and YZ organized all the data. FZ, QW and HC analyzed all the information. FZ and LT drafted the manuscript. FZ and NT revised the article critically. All the authors read and approved the final manuscript.

Acknowledgements

All the authors appreciated the staffs from primary health care centers for materials supplement and participant organization.

Competing interests

All the authors declared that there were no competing interests among them.

Fundings

The surveys in Luzhou were supported by the grants from the National Clinical Research Center for Metabolic Diseases (2013BAI09B13), the National Key New Drug Creation and Manufacturing Program of Ministry of Science and Technology (2012ZX09303006-001). The screenings in Wenjiang were supported by the National Key Technology R&D Program of China (Grant No. 2009BAI80B01 and No. 2009BAI80B02) and Sichuan Provincial Science and Technology Foundation (Grant No. 150022).

Data sharing statement

A supplementary profile will be available online which contains comprehensive figure

and table of used input data. Inquiries about additional unpublished data could be contacted with the corresponding author.

References

1. Xu Y, Wang L, He J, Bi Y, Li M, Wang T, Wang L, Jiang Y, Dai M, Lu J, Xu M, Li Y, Hu N, Li J, Mi S, Chen CS, Li G, Mu Y, Zhao J, Kong L, Chen J, Lai S, Wang W, Zhao W, Ning G; 2010 China Noncommunicable Disease Surveillance Group. Prevalence and Control of Diabetes in Chinese Adults. *JAMA.* 2013; 310 (9): 948-958.
2. Yang W, Lu J, Weng J, Jia W, Ji L, Xiao J, Shan Z, Liu J, Tian H, Ji Q, Zhu D, Ge J, Lin L, Chen L, Guo X, Zhao Z, Li Q, Zhou Z, Shan G, He J; China National Diabetes and Metabolic Disorders Study Group. Prevalence of Diabetes among Men and Women in China. *N Engl J Med.* 2010; 362: 1090-101.
3. Rydén L, Grant PJ, Anker SD, Berne C, Cosentino F, Danchin N, Deaton C, Escaned J, Hammes HP, Huikuri H, Marre M,Marx N, Mellbin L, Ostergren J, Patrono C, Seferovic P, Uva MS, Taskinen MR, Tendera M, Tuomilehto J, Valensi P, Zamorano JL; ESC Committee for Practice Guidelines (CPG), Zamorano JL, Achenbach S, Baumgartner H, Bax JJ, Bueno H, Dean V, Deaton C, Erol C, Fagard R, Ferrari R, Hasdai D, Hoes AW, Kirchhof P, Knuuti J, Kolh P, Lancellotti P, Linhart A, Nihoyannopoulos P, Piepoli MF, Ponikowski P, Sirnes PA, Tamargo JL, Tendera M, Torbicki A,Wijns W, Windecker S; Document Reviewers, De Backer G, Sirnes PA, Ezquerra EA, Avogaro A, Badimon L, Baranova E, Baumgartner H, Betteridge J,Ceriello A, Fagard R, Funck-Brentano C, Gulba DC, Hasdai D, Hoes AW, Kjekshus JK, Knuuti J, Kolh P, Lev E, Mueller C, Neyses L, Nilsson PM, Perk J,Ponikowski P, Reiner Z, Sattar N, Schächinger V, Scheen A, Schirmer H, Strömberg A, Sudzhaeva S, Tamargo JL, Viigimaa M, Vlachopoulos C, Xuereb RG. ESC Guidelines on diabetes, pre-diabetes, and cardiovascular diseases developed in collaboration with the EASD. *European Heart Journal.* 2013; 34: 3035–3087.
4. Tanne D, Koren-Morag N, Goldbourt U. Fasting plasma glucose and risk of incident ischemic stroke or transient ischemic attacks: a prospective cohort study. *Stroke.* 2004; 35:2351-5.
5. Norhammar A, Tenerz A, Nilsson G, Hamsten A, Efendíc S, Rydén L, Malmberg K. Glucose metabolism in patients with acute myocardial infarction and no previous diagnosis of diabetes mellitus: a prospective study. *Lancet.* 2002; 359: 2140-4.
6. Chinese Diabetes Society. China Guideline for Type 2 Diabetes. *Chinese Diabetes Society.* 2010. (Chinese)
7. Chen L, Magliano DJ, Balkau B, Colagiuri S, Zimmet PZ, Tonkin AM, Mitchell P, Phillips PJ, Shaw JE. AUSDRISK: an Australian type 2 diabetes risk assessment tool based on demographic, lifestyle and simple anthropometric measures. *Medical Journal of Australia.* 2010;192:(4)197–202.
8. Lindstrom J, Tuomilehto J. The diabetes risk score: a practical tool to predict type 2 diabetes risk. *Diabetes Care.* 2003;26:(3)725–731.

9. Mohan V, Sandeep S, Deepa M, Gokulakrishnan K, Datta M, Deepa R. A diabetes risk score helps identify metabolic syndrome and cardiovascular risk in Indians – the Chennai Urban Rural Epidemiology Study (CURES-38). *Diabetes, Obesity and Metabolism.* 2007;9:(3)337–343.
10. Lu J, Bi Y, Wang T, Wang W, Mu Y, Zhao J, Liu C, Chen L, Shi L, Li Q, Wan Q, Wu S, Qin G, Yang T, Yan L, Liu Y, Wang G, Luo Z, Tang X, Chen G, Huo Y, Gao Z, Su Q, Ye Z, Wang Y, Deng H, Yu X, Shen F, Chen L, Zhao L, Dai M, Xu M, Xu Y, Chen Y, Lai S, Ning G. The relationship between insulin-sensitive obesity and cardiovascular diseases in a Chinese population: Results of the REACTION study. *International Journal of Cardiology.* 2014; 172: 388–394.
11. Bi Y, Lu J, Wang W, Mu Y, Zhao J, Liu C, Chen L, Shi L, Li Q, Wan Q, Wu S, Yang T, Yan L, Liu Y, Wang G, Luo Z, Tang X, Chen G, Huo Y, Gao Z, Su Q, Ye Z,Wang Y, Qin G, Deng H, Yu X, Shen F, Chen L, Zhao L, Zhang J, Sun J, Dai M, Xu M, Xu Y, Chen Y, Lai S, Bloomgarden ZT, Li D, Ning G. Cohort profile: Risk evaluation of cancers in Chinese diabetic individuals: A longitudinal (REACTION) study. *J Diabetes.* 2014; 6 (2): 147–57.
12. American Diabetes Association. Standards of medical care in diabetes–2010. *Diabetes Care.* 2011; 33 (Suppl. 1): S11–61.
13. DeLong ER, DeLong DM, Clarke-Pearson DL. Comparing the areas under two or more correlated receiver operating characteristic curves: a nonparametric approach. *Biometrics.* 1988; 44 (3):837-45.
14. Hadaegh F, Derakhshan A, Zafari N, Khalili D, Mirbolouk M, Saadat N, Azizi F. Pre-diabetes tsunami: incidence rates and risk factors of pre-diabetes and its different phenotypes over9 years of follow-up. *Diabet Med*. 2015 Nov 25. doi: 10.1111/dme.13034.
15. Lyssenko V, Almgren P, Anevski D, Perfekt R, Lahti K, Nissén M, Isomaa B, Forsen B, Homström N, Saloranta C, Taskinen MR, Groop L, Tuomi T, Botnia study group. Predictors of and longitudinal changes in insulin sensitivity and secretion preceding onset of type 2 diabetes. *Diabetes.* 2005;54(1):166-74.
16. Xu Z, Qi X, Dahl AK, Xu W. Waist-to-height ratio is the best indicator for undiagnosed type 2 diabetes. *Diabet Med*. 2013;30(6):e201-7.
17. Hu D, Xie J, Fu P, Zhou J, Yu D, Whelton PK, He J, Gu D. Central rather than overall obesity is related to diabetes in the Chinese population: the InterASIA study. *Obesity (Silver Spring)*. 2007 Nov;15(11):2809-16.
18. Jung SH, Ha KH, Kim DJ. Visceral fat mass has stronger associations with diabetes and prediabetes than other anthropometric obesity indicators among Korean adults. *Yonsei Med J.* 2016 May;57(3):674-80.
19. Hayashi T, Boyko EJ, Leonetti DL, McNeely MJ, Newell-Morris L, Kahn SE, Fujimoto WY. Visceral adiposity and the risk of impaired glucose tolerance: a prospective study among Japanese Americans. *Diabetes Care*. 2003;26(3):650-5.
20. Alperet DJ, Lim WY, Mok-Kwee Heng D, Ma S, van Dam RM. Optimal anthropometric measures and thresholds to identify undiagnosed type 2 diabetes in three major Asian

ethnic groups. *Obesity (Silver Spring).* 2016 Oct;24(10):2185-93.

21. Nathan DM, Davidson MB, DeFronzo RA, Heine RJ, Henry RR, Pratley R, Zinman B, American Diabetes Association. Impaired fasting glucose and impaired glucose tolerance: implications for care. *Diabetes Care*. 2007 Mar;30(3):753-9.
22. Huang L, Tong Y, Zhang F, Yang Q, Li D, Xie S, Li Y, Cao H, Tang L, Zhang X, Tong N. Increased acyl ghrelin but decreased total ghrelin and unacyl ghrelin in Chinese Han people with impaired fasting glucose combined with impaired glucose tolerance. *Peptides*. 2014 Oct;60:86-94.
23. Lü Q, Tong N, Liu Y, Li N, Tang X, Zhao J, Cao H, Li D, Gou L, Zhang Y, Wan J, Jiang L. Community-based population data indicates the significant alterations of insulinresistance , chronic inflammation and urine ACR in IFG combined IGT group amongprediabetic pop ulation. *Diabetes Res Clin Pract*. 2009 Jun;84(3):319-24.
24. Zhang F, Tang X, Cao H, Lü Q, Li N, Liu Y, Zhang X, Zhang Y, Cao M, Wan J, An Z, Tong N. Impaired secretion of total glucagon-like peptide-1 in people with impaired fasting glucose combined impaired glucose tolerance. *Int J Med Sci.* 2012;9(7):574-81.
25. Tulloch-Reid MK, Williams DE, Looker HC, Hanson RL, Knowler WC. Do measures of body fat distribution provide information on the risk of type 2 diabetes in addition to measures of general obesity? Comparison of anthropometric predictors of type 2 diabetes in Pima Indians. *Diabetes Care*. 2003 Sep;26(9):2556-61.
26. Chei CL, Iso H, Yamagishi K, Tanigawa T, Cui R, Imano H, Kiyama M, Kitamura A, Sato S, Shimamoto T. Body fat distribution and the risk of hypertension and diabetes among Japanese men and women. *Hypertens Res.* 2008 May;31(5):851-7.
27. MacKay MF, Haffner SM, Wagenknecht LE, D'Agostino RB Jr, Hanley AJ. Prediction of type 2 diabetes using alternate anthropometric measures in a multi-ethnic cohort: the insulin resistance atherosclerosis study. *Diabetes Care.* 2009 May;32(5):956-8.
28. Son YJ, Kim J, Park HJ, Park SE, Park CY, Lee WY, Oh KW, Park SW, Rhee EJ. Association of Waist-Height Ratio with Diabetes Risk: A 4-Year Longitudinal Retrospective Study. *Endocrinol Metab (Seoul).* 2016 Mar;31(1):127-33.
29. Xu F, Wang YF, Lu L, Liang Y, Wang Z, Hong X, Li J. Comparison of anthropometric indices of obesity in predicting subsequent risk of hyperglycemia among Chinese men and women in Mainland China. *Asia Pac J Clin Nutr.* 2010;19(4):586-93.
30. Browning LM, Hsieh SD, Ashwell M. A systematic review of waist-to-height ratio as a screening tool for the prediction of cardiovascular disease and diabetes: 0·5 could be a suitable global boundary value. *Nutrition Research Reviews.* 2010; 23: 247–269.

Table 1. General measurements of all subjects at baseline who progressed to diverse hyperglycemic status during follow-up

	NGT (n = 972)	Isolated IFG (n = 159)	Isolated IGT (n = 342)	IFG+IGT (n = 152)	NDDM (n = 260)	overall P value
Follow-up time (year)	3.00 (2.92-4.17)‡	3.00 (2.92-4.17)‡	2.92 (2.92-3.17)*†	3.00 (2.92-3.17)	3.00 (2.92-3.17)	0.000
Age (year)	53.00 (46.00-59.00)†‡§¶	55.00 (48.00-62.00)*¶	59.00 (49.00-65.00)*	56.00 (49.00-62.00)*¶	60.00 (53.50-65.00)*†§	0.000
Female (N/n%)	675 (69.44%)	96 (60.38%)	220 (64.33%)	97 (63.82%)	166 (63.85%)	0.075
Height (cm)	158.00 (153.10-164.00)	159.45 (154.00-165.52)	157.00 (152.00-162.70)	157.10 (154.00-164.00)	156.00 (152.00-163.20)	0.492
Weight (kg)	58.00 (52.00-65.00)	60.50 (53.99-66.85)	60.00 (53.00-66.20)	62.10 (56.70-69.50)	62.00 (55.00-69.75)	0.498
Hip circumference (cm)	93.00 (88.20-97.20)	94.00 (90.00-99.00)	95.00 (90.20-100.00)	96.00 (92.00-100.30)	96.00 (92.00-101.00)	0.879
SBP (mmHg)	115.67 (105.33-128.67)‡§¶	118.50 (107.46-133.00)¶	122.50 (109.33-136.67)*¶	123.00 (114.00-137.67)*	130.67 (118.67-142.17)*†‡	0.000
DBP (mmHg)	74.33 (68.00-81.33)§¶	77.00 (70.00-83.75)	76.33 (69.00-83.33)¶	77.50 (72.33-82.67)*	79.00 (72.33-88.17)*‡	0.000
FPG (mmol/L)	5.08 (4.83-5.29)†§¶	5.20 (4.98-5.38)*	5.11 (4.90-5.33)	5.16 (4.93-5.36)*	5.16 (4.92-5.36)*	0.000
2hPG (mmol/L)	6.15 (5.40-6.88)‡§	6.14 (5.45-6.93)	6.40 (5.67-7.09)*	6.54 (5.85-7.10)*	6.33 (5.50-7.08)	0.000
HbA1c (%)	5.60 (5.30-5.90)‡§¶	5.70 (5.48-5.90)	5.70 (5.40-5.90)*	5.70 (5.50-6.00)*	5.70 (5.40-6.00)*	0.000
TG (mmol/L)	1.10 (0.80-1.60)	1.12 (0.80-1.63)	1.11 (0.84-1.59)	1.14 (0.89-1.60)	1.07 (0.81-1.50)	0.494
TC (mmol/L)	4.46 ± 1.01	4.45 ± 1.17	4.50 ± 1.02	4.72 ± 1.14	4.52 ± 1.10	0.062
HDL-c (mmol/L)	1.32 (1.09-1.60)	1.32 (1.05-1.52)	1.30 (1.08-1.56)	1.36 (1.20-1.57)	1.31 (1.09-1.60)	0.376
LDL-c (mmol/L)	2.51 (2.04-3.03)	2.44 (1.97-3.09)	2.53 (1.99-2.99)	2.65 (2.06-3.17)	2.45 (1.95-3.01)	0.688
Family history of diabetes (N/%)	119 (12.24%)	12 (7.55%)	29 (8.48%)	18 (11.84%)	29 (11.15%)	0.214
Current smoker (N/%)	137 (14.10%)	28 (17.61%)	42 (12.28%)	18 (11.84%)	44 (16.92%)	0.307
Physical activity (N/%)	719 (73.97%)	107 (67.30%)	255 (74.56%)	113 (74.34%)	195 (75.00%)	0.435

NGT, normal glucose tolerance; isolated IFG, isolated impaired fasting glucose; isolated IGT, isolated impaired glucose tolerance; IFG+IGT, IFG combined IGT;

For peer review only

NDDM, newly-diagnosed diabetes mellitus; SBP, systolic blood pressure; DBP, diastolic blood pressure; FPG, fasting plasma glucose; 2hPG, 2 hour plasma glucose (after oral glucose tolerance test); TG, triglyceride; TC, total cholesterol; HDL-c, high-density lipoprotein cholesterol; LDL-c, low-density lipoprotein cholesterol.

Data are expressed as means± SD or median (interquartile range) or N (%).

Chi-square test was used to compare gender compositions, family history of diabetes, current smoking status and physical activity among five groups. If needed, chi-square segmentation was applied for further comparisons between any two subgroups with an adjusted significance level (a' = 0.005).

Kruskal-Wallis H analysis was applied for follow-up time among five groups. Mann-Whitney U analysis was performed for comparison within any two subgroups additionally (a' = 0.005).

One-way ANOVA analysis was used for the rest measurements among five groups, while LSD analysis was applied for age, SBP, DBP, FPG, 2hPG and HbA1c comparisons between any two subgroups (a' = 0.005).

* vs. NGT, $p < 0.005$; † vs. isolated IFG, $p < 0.005$; ‡ vs. isolated IGT, $P < 0.005$; § vs. IFG+IGT, $P < 0.005$; ¶ vs. NDDM, $P < 0.005$.

For peer review only

Table 2. Anthropometric indicators of participants who further transited to diverse hyperglycemic statuses at follow-up and baseline

	NGT (n = 972)	Isolated IFG (n = 159)	Isolated IGT (n = 342)	IFG+IGT (n = 152)	NDDM (n = 260)	overall P value
At follow-up survey						
WHtR (cm/cm)	0.51 (0.47-0.55)‡§¶	0.52 (0.48-0.56)§¶	0.53 (0.49-0.57)*§¶	0.54 (0.51-0.59)*†‡	0.56 (0.52-0.60)*†‡	0.000
BMI (kg/m^2)	23.46 (21.77-25.53)†‡§¶	24.27 (22.49-26.17)*¶	24.44 (22.63-26.50)*¶	25.09 (23.62-27.01)*	25.73 (23.29-27.82)*†‡	0.000
Waist circumference (cm)	80.65 (74.00-87.00)†‡§¶	82.80 (77.00-91.00)*§¶	84.00 (78.00-90.00)*§¶	86.70 (80.28-93.00)*†‡	88.00 (82.00-95.00)*†‡	0.000
WHR (cm/cm)	0.86 (0.81-0.91)†‡§¶	0.88 (0.84-0.92)*¶	0.88 (0.83-0.92)*¶	0.90 (0.86-0.94)*	0.91 (0.87-0.95)*†‡	0.000
At baseline survey						
WHtR (cm/cm)	0.50 ± 0.05†‡§¶	0.52 ± 0.06*§¶	0.53 ± 0.05*¶	0.54 ± 0.05*†	0.55 ± 0.06*†‡	0.000
BMI (kg/m^2)	23.03 (21.23-25.16)‡§¶	23.31 (21.56-25.64)¶	24.03 (22.10-26.22)*	24.98 (23.47-26.67)*	25.42 (23.17-27.22)*†	0.000
Waist circumference (cm)	79.00 (73.00-86.00)	82.00 (76.00-89.00)	83.00 (77.10-89.00)	87.00 (81.00-91.28)	86.00 (80.00-93.00)	0.282
WHR (cm/cm)	0.86 (0.81-0.90)¶	0.87 (0.92-0.92)	0.87 (0.82-0.91)	0.89 (0.86-0.93)	0.90 (0.86-0.94)*	0.010

NGT, normal glucose tolerance; isolated IFG, isolated impaired fasting glucose; isolated IGT, isolated impaired glucose tolerance; IFG+IGT, IFG combined IGT; NDDM, newly-diagnosed diabetes mellitus; WHtR, waist-to-height ratio; BMI, body mass index; WHR, waist-to-hip ratio.

Data are expressed as median (interquartile range).

At follow-up survey: One-way ANOVA analysis was used for WHtR, BMI and WC among the five glucose metabolic groups. LSD analysis was applied for the further comparisons between any two subgroups (a' = 0.005).Kruskal-Wallis H analysis was applied for WHR among the five groups and Mann-Whitney U analysis was performed for the following comparisons within any two subgroups (a' = 0.005).

At baseline survey: One-way ANOVA analysis was used for all indices among the five glucose metabolic groups. LSD analysis was applied for WHtR, BMI and WHR between any two subgroups' comparison (a' = 0.005).

* vs. NGT, p＜0.005; † vs. isolated IFG, p＜0.005; ‡ vs. isolated IGT, P < 0.005; § vs. IFG+IGT, P < 0.005; ¶ vs. NDDM, P < 0.005.

For peer review only

Table 3. ROC curve analysis of baseline anthropometric indices for predicting future hyperglycemic disorders

	AUC	SE	P value	95%CI	Cut-off point	Youden's value	Sensitivity	Specificity	DeLong's test (P value) WHtR (cm/cm)	BMI (kg/m^2)	Waist circumference (cm)	WHR (cm/cm)
Isolated IFG												
WHtR (cm/cm)	0.578	0.025	0.002	(0.529-0.626)	0.51	0.151	54.90%	60.19%	-	0.010	0.201	0.611
BMI (kg/m^2)	0.544	0.025	0.081	(0.495-0.593)	21.36	0.078	80.40%	27.43%	0.010	-	0.023	0.421
Waist circumference (cm)	0.592	0.024	0.000	(0.545-0.639)	77.10	0.148	71.24%	43.54%	0.201	0.023	-	0.195
Women	0.584	0.031	0.010	(0.524-0.644)	75.00	0.166	74.44%	42.11%	-	-	-	-
Men	0.579	0.041	0.050	(0.526-0.631)	87.00	0.165	49.21%	67.24%	-	-	-	-
WHR (cm/cm)	0.567	0.026	0.008	(0.537-0.597)	0.88	0.128	47.06%	65.71%	0.611	0.421	0.195	-
Women	0.568	0.033	0.036	(0.504-0.632)	0.85	0.140	57.78%	56.19%	-	-	-	-
Men	0.525	0.042	0.534	(0.471-0.578)	0.90	0.095	53.97%	55.52%	-	-	-	-
Isolated IGT												
WHtR (cm/cm)	0.634	0.017	0.000	(0.600-0.667)	0.51	0.214	62.24%	59.12%	-	0.003	0.006	0.000
BMI (kg/m^2)	0.591	0.018	0.000	(0.556-0.627)	22.68	0.155	68.88%	46.64%	0.003	-	0.178	0.223
Waist circumference (cm)	0.610	0.017	0.000	(0.576-0.645)	78.00	0.197	71.90%	47.81%	0.006	0.178	-	0.001
Women	0.635	0.021	0.000	(0.593-0.676)	78.00	0.260	68.42%	57.59%	-	-	-	-
Men	0.542	0.032	0.174	(0.480-0.605)	87.80	0.132	43.44%	69.76%	-	-	-	-
WHR (cm/cm)	0.567	0.018	0.000	(0.539-0.594)	0.86	0.123	61.63%	50.64%	0.000	0.223	0.001	-
Women	0.587	0.022	0.000	(0.544-0.630)	0.82	0.154	77.03%	38.39%	-	-	-	-
Men	0.524	0.032	0.433	(0.463-0.586)	0.89	0.098	57.38%	52.41%	-	-	-	-
IFG+IGT												

For peer review only

WHtR (cm/cm)	0.713	0.022	0.000	(0.670-0.755)	0.53	0.351	62.33%	72.79%	-	0.106	0.556	0.026
BMI (kg/m^2)	0.685	0.022	0.000	(0.642-0.729)	23.38	0.316	77.40%	54.22%	0.106	-	0.254	0.492
Waist circumference (cm)	0.706	0.021	0.000	(0.665-0.748)	79.80	0.351	82.88%	52.19%	0.556	0.254	-	0.032
Women	0.732	0.026	0.000	(0.682-0.783)	79.80	0.420	79.57%	62.38%	-	-	-	-
Men	0.656	0.039	0.000	(0.579-0.733)	90.30	0.242	43.40%	80.76%	-	-	-	-
WHR (cm/cm)	0.667	0.022	0.000	(0.638-0.695)	0.87	0.274	69.18%	58.23%	0.026	0.492	0.032	-
Women	0.686	0.027	0.000	(0.633-0.739)	0.83	0.312	86.02%	45.20%	-	-	-	-
Men	0.631	0.038	0.003	(0.556-0.705)	0.92	0.261	54.72%	71.38%	-	-	-	-
NDDM												
WHtR (cm/cm)	0.730	0.017	0.000	(0.696-0.764)	0.52	0.366	74.21%	62.43%	-	0.000	0.001	0.010
BMI (kg/m^2)	0.677	0.020	0.000	(0.639-0.716)	24.32	0.315	64.68%	66.81%	0.000	-	0.093	0.596
Waist circumference (cm)	0.700	0.018	0.000	(0.665-0.735)	78.00	0.292	81.35%	47.81%	0.001	0.093	-	0.429
Women	0.714	0.021	0.000	(0.673-0.756)	77.10	0.344	81.76%	52.63%	-	-	-	-
Men	0.686	0.033	0.000	(0.622-0.750)	88.00	0.298	56.99%	72.85%	-	-	-	-
WHR (cm/cm)	0.688	0.018	0.000	(0.661-0.715)	0.88	0.304	67.73%	62.71%	0.010	0.596	0.429	-
Women	0.696	0.022	0.000	(0.653-0.738)	0.84	0.301	79.75%	50.31%	-	-	-	-
Men	0.681	0.030	0.000	(0.622-0.740)	0.92	0.299	60.22%	69.66%	-	-	-	-

ROC, receiver operating characteristic; isolated IFG, isolated impaired fasting glucose; isolated IGT, isolated impaired glucose tolerance; IFG+IGT, IFG combined IGT; NDDM, newly-diagnosed diabetes mellitus; AUC, area under curve; SE, standard error; CI, confidence interval; WHtR, waist-to-height ratio; BMI, body mass index; WHR, waist-to-hip ratio.

For peer review only

Table 4. Multivariable analysis of baseline anthropometric indices in relation to subsequent incidences of pre-diabetes and NDDM

	Isolated IFG			Isolated IGT			IFG+IGT			NDDM		
	HR	95% CI	P	HR	95% CI	P	HR	95% CI	P	HR	95% CI	P
WHtR (cm/cm)	1.471	(0.901-2.402)	0.123	1.951	(1.550-2.457)	0.000	3.002	(2.137-4.216)	0.000	2.765	(2.065-3.703)	0.000
BMI (kg/m^2)	1.186	(0.699-2.012)	0.526	1.571	(1.241-1.988)	0.000	3.298	(2.224-4.892)	0.000	2.305	(1.773-2.998)	0.000
Waist circumference (cm)	1.603	(1.112-2.310)	0.011	1.644	(1.275-2.118)	0.000	4.570	(2.948-7.084)	0.000	2.666	(1.886-3.769)	0.000
WHR (cm/cm)	1.182	(0.739-1.889)	0.486	0.972	(0.724-1.304)	0.848	1.571	(1.003-2.465)	0.048	1.706	(1.196-2.433)	0.003

NDDM, newly-diagnosed diabetes mellitus; isolated IFG, isolated impaired fasting glucose; isolated IGT, isolated impaired glucose tolerance; IFG+IGT, IFG combined IGT; HR, hazard ratio; CI, confidence interval; WHtR, waist-to-height ratio; BMI, body mass index; WHR, waist-to-hip ratio.

Cox proportional hazards models were used to calculate HR and 95% CI. A univariable analysis was performed for each potential risk factor firstly, including age (years), gender (male/female), systolic blood pressure (mmHg), diastolic blood pressure (mmHg), fasting plasma glucose (mmol/L), 2h plasma glucose (mmol/L) (after oral glucose tolerance test), HbA1c (%), total cholesterol (mmol/L), triglyceride (mmol/L), high-density lipoprotein cholesterol (mmol/L), low-density lipoprotein cholesterol (mmol/L), diabetes family history (yes/no), current smoking status (yes/no), physical activity situation (none/mild/robust), WHtR (low/high), BMI (low/high), WC (low/high) and WHR (low/high). The four anthropometric indicators were dichotomized into low or high level by using cut-off values derived from previous ROC curve analysis. Then those risk factors with a P-value < 0.2 in univariable analysis were selected to enter the multivariable model.

For peer review only

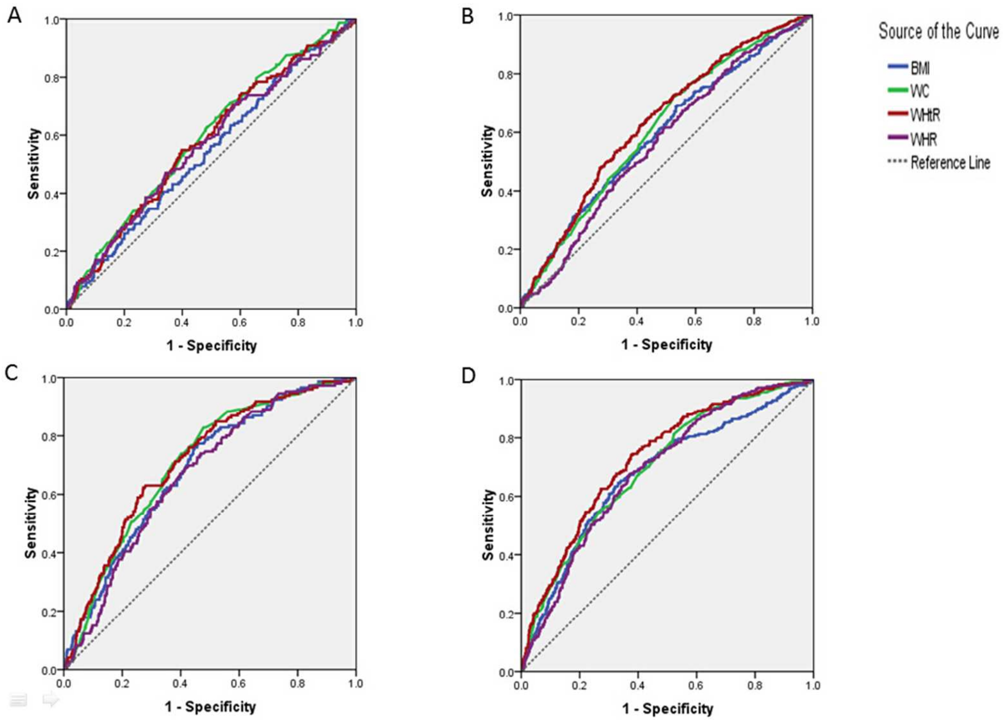

Figure 1. ROC curves of baseline anthropometric indices in subjects who further progressed to (A) isolated IFG, (B) isolated IGT, (C) IFG+IGT and (D) NDDM. ROC, receiver operating characteristic; isolated IFG, isolated impaired fasting glucose; isolated IGT, isolated impaired glucose tolerance; IFG+IGT, IFG combined with IGT; NDDM, newly-diagnosed diabetes mellitus; WHtR, waist-to-height ratio; BMI, body mass index; WHR, waist-to-hip ratio; WC, waist circumference.

266x191mm (96 x 96 DPI)

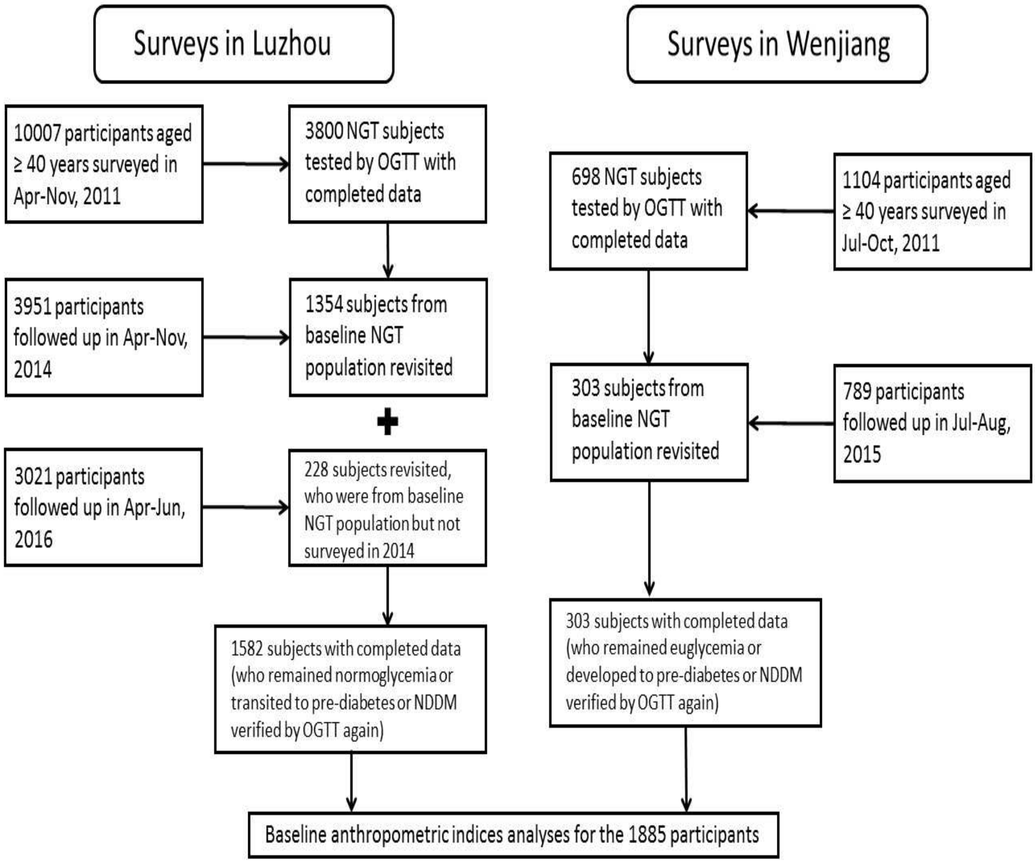

Supplemental Figure 1. Flow-chart of study design. NGT, normal glucose tolerance; OGTT, oral glucose tolerance test; NDDM, newly-diagnosed diabetes mellitus.

For peer review only - http://bmjopen.bmj.com/site/about/guidelines.xhtml

Supplemental Table 1. Baseline characteristics of all participants screened in Luzhou and Wenjiang

	Luzhou baseline survey			P	Wenjiang baseline survey			P
	Total (n = 1582)	Men (n = 495)	Women (n = 1087)		Total (n = 303)	Men (n = 154)	Women (n = 149)	
Age (year)	57.00 (50.00-63.00)	60.00 (54.00-66.00)	56.00 (49.00-61.00)	0.000	47.00 (43.00-54.00)	47.00 (43.00-54.00)	46.00 (42.00-52.00)	0.161
Female (N/n%)	1087 (68.71%)	-	-		149 (49.17%)	-	-	
Height (cm)	157.00 (152.40-163.00)	165.00 (160.50-169.00)	154.55 (151.00-158.20)	0.180	161.28 ± 7.60	166.46 ± 5.27	155.56 ± 5.32	0.000
Weight (kg)	59.00 (53.00-65.30)	65.00 (58.30-72.00)	56.50 (51.20-62.50)	0.000	61.88 ± 11.36	67.66 ± 8.84	55.49 ± 10.38	0.000
Hip circumference (cm)	94.00 (89.20-99.00)	95.00 (90.00-100.00)	94.00 (89.00-98.20)	0.655	93.47 ± 6.04	95.01 ± 5.43	91.76 ± 6.24	0.000
SBP (mmHg)	120.67 (108.67-135.67)	126.00 (113.83-140.17)	119.00 (107.00-133.37)	0.000	114.90 ± 14.27	118.20 ± 14.02	111.26 ± 13.69	0.000
DBP (mmHg)	75.33 (69.00-82.67)	79.00 (71.67-88.17)	74.00 (68.00-80.67)	0.000	78.40 ± 16.26	81.15 ± 10.84	75.36 ± 20.27	0.001
FPG (mmol/L)	5.14 (4.93-5.34)	5.15 (4.96-5.38)	5.13 (4.92-5.32)	0.011	4.90 (4.60-5.10)	4.90 (4.70-5.20)	4.80 (4.60-5.10)	0.286
2hPG (mmol/L)	6.32 (5.57-7.00)	6.32 (5.57-6.98)	6.32 (5.57-7.01)	0.777	6.00 (5.00-6.70)	5.90 (5.03-5.78)	6.00 (5.00-6.80)	0.541
HbA1c (%)	5.70 (5.40-5.90)	5.70 (5.50-5.95)	5.70 (5.40-5.90)	0.069	5.48 ± 0.42	5.51 ± 0.38	5.45 ± 0.45	0.228
TG (mmol/L)	1.33 ± 0.94	1.29 ± 0.84	1.34 ± 0.98	0.388	1.10 (0.80-1.80)	1.50 (0.90-2.18)	0.90 (0.70-1.50)	0.000
TC (mmol/L)	4.44 (3.75-5.18)	4.32 (3.63-5.12)	4.50 (3.82-5.19)	0.017	4.53 ± 0.83	4.60 ± 0.82	4.45 ± 0.83	0.178
HDL-c (mmol/L)	1.28 (1.06-1.52)	1.26 (1.03-1.52)	1.29 (1.08-1.52)	0.107	1.59 ± 0.39	1.47 ± 0.32	1.71 ± 0.41	0.000
LDL-c (mmol/L)	2.52 ± 0.77	2.47 ± 0.74	2.54 ± 0.79	0.078	2.86 ± 0.75	2.92 ± 0.70	2.79 ± 0.80	0.252
WHtR (cm/cm)	0.52 (0.48-0.56)	0.53 (0.49-0.56)	0.52 (0.48-0.56)	0.900	0.49 (0.45-0.53)	0.50 (0.47-0.53)	0.47 (0.44-0.51)	0.000
BMI (kg/m^2)	23.74 (21.61-26.00)	24.04 (21.81-26.14)	23.68 (21.51-25.92)	0.462	23.49 (21.64-25.59)	24.40 (22.33-26.03)	22.44 (21.10-24.24)	0.000
Waist circumference (cm)	82.00 (76.00-89.00)	87.00 (80.00-92.45)	80.00 (75.00-87.10)	0.201	79.00 (72.00-86.00)	84.00 (79.00-89.00)	73.00 (69.75-78.00)	0.000
WHR (cm/cm)	0.86 (0.80-0.91)	0.89 (0.83-0.94)	0.85 (0.79-0.90)	0.476	0.86 (0.80-0.91)	0.83 (0.78-0.89)	0.87 (0.81-0.92)	0.256
Outcomes at follow-up: N/total (%)								
NGT	757 (47.85%)	203 (41.01%)	554 (50.97%)	-	215 (70.96%)	103 (66.88%)	112 (75.17%)	-
Isolated IFG	131 (8.28%)	51 (10.30%)	80 (7.34%)	-	28 (9.24%)	12 (7.79%)	16 (10.74%)	-
Isolated IGT	304 (19.22%)	103 (20.81%)	201 (18.49%)	-	38 (12.54%)	24 (15.58%)	14 (9.40%)	-
IFG+IGT	137 (8.66%)	46 (9.29%）	91 (8.37%)	-	15 (4.95%)	11 (7.14%)	4 (2.68%)	-

For peer review only

NDDM	253 (15.99%)	92 (18.59%)	161 (14.81%)	-	7 (2.31%)	4 (2.61%)	3 (2.01%)	-

SBP, systolic blood pressure; DBP, diastolic blood pressure; FPG, fasting plasma glucose; 2hPG, 2 hour plasma glucose (after oral glucose tolerance test); TG, triglyceride; TC, total cholesterol; HDL-c, high-density lipoprotein cholesterol; LDL-c, low-density lipoprotein cholesterol; WHtR, waist-to-height ratio; BMI, body mass index; WHR, waist-to-hip ratio; NGT, normal glucose tolerance; isolated IFG, isolated impaired fasting glucose; isolated IGT, isolated impaired glucose tolerance; IFG+IGT, IFG combined IGT; NDDM, newly-diagnosed diabetes mellitus.

Data are expressed as means± SD or median (interquartile range) or N (%).

Mann-Whitney U analysis was used for DBP and BMI in Luzhou, TG and HDL-c in Wenjiang; one-way ANOVA analysis was used for the rest measurements in two surveys.

P value of men vs. women

For peer review only

STROBE 2007 (v4) checklist of items to be included in reports of observational studies in epidemiology*
Checklist for cohort, case-control, and cross-sectional studies (combined)

Section/Topic	Item #	Recommendation	Reported on page #
**Title and abstract**	1	(*a*) Indicate the study's design with a commonly used term in the title or the abstract	1, 2
		(*b*) Provide in the abstract an informative and balanced summary of what was done and what was found	2
**Introduction**			
Background/rationale	2	Explain the scientific background and rationale for the investigation being reported	4
Objectives	3	State specific objectives, including any pre-specified hypotheses	4
**Methods**			
Study design	4	Present key elements of study design early in the paper	4, 5
Setting	5	Describe the setting, locations, and relevant dates, including periods of recruitment, exposure, follow-up, and data collection	4. 5
Participants	6	(*a*) *Cohort study*—Give the eligibility criteria, and the sources and methods of selection of participants. Describe methods of follow-up<br>*Case-control study*—Give the eligibility criteria, and the sources and methods of case ascertainment and control selection. Give the rationale for the choice of cases and controls<br>*Cross-sectional study*—Give the eligibility criteria, and the sources and methods of selection of participants	4, 5
		(*b*) *Cohort study*—For matched studies, give matching criteria and number of exposed and unexposed<br>*Case-control study*—For matched studies, give matching criteria and the number of controls per case	NA
Variables	7	Clearly define all outcomes, exposures, predictors, potential confounders, and effect modifiers. Give diagnostic criteria, if applicable	5, 6
Data sources/ measurement	8*	For each variable of interest, give sources of data and details of methods of assessment (measurement). Describe comparability of assessment methods if there is more than one group	*5, 6*
Bias	9	Describe any efforts to address potential sources of bias	NA
Study size	10	Explain how the study size was arrived at	4, 5
Quantitative variables	11	Explain how quantitative variables were handled in the analyses. If applicable, describe which groupings were chosen and why	5, 6
Statistical methods	12	(*a*) Describe all statistical methods, including those used to control for confounding	6
		(*b*) Describe any methods used to examine subgroups and interactions	6
		(*c*) Explain how missing data were addressed	6
		(*d*) *Cohort study*—If applicable, explain how loss to follow-up was addressed<br>*Case-control study*—If applicable, explain how matching of cases and controls was addressed	6

For peer review only

		Cross-sectional study—If applicable, describe analytical methods taking account of sampling strategy	
		(*e*) Describe any sensitivity analyses	NA
**Results**			
Participants	13*	(a) Report numbers of individuals at each stage of study—eg numbers potentially eligible, examined for eligibility, confirmed eligible, included in the study, completing follow-up, and analysed	7
		(b) Give reasons for non-participation at each stage	5
		(c) Consider use of a flow diagram	5
Descriptive data	14*	(a) Give characteristics of study participants (eg demographic, clinical, social) and information on exposures and potential confounders	6
		(b) Indicate number of participants with missing data for each variable of interest	NA
		(c) *Cohort study*—Summarise follow-up time (eg, average and total amount)	5
Outcome data	15*	*Cohort study*—Report numbers of outcome events or summary measures over time	6
		Case-control study—Report numbers in each exposure category, or summary measures of exposure	NA
		Cross-sectional study—Report numbers of outcome events or summary measures	NA
Main results	16	(*a*) Give unadjusted estimates and, if applicable, confounder-adjusted estimates and their precision (eg, 95% confidence interval). Make clear which confounders were adjusted for and why they were included	7, 8
		(*b*) Report category boundaries when continuous variables were categorized	6-8
		(*c*) If relevant, consider translating estimates of relative risk into absolute risk for a meaningful time period	7, 8
Other analyses	17	Report other analyses done—eg analyses of subgroups and interactions, and sensitivity analyses	NA
**Discussion**			
Key results	18	Summarise key results with reference to study objectives	8
Limitations	19	Discuss limitations of the study, taking into account sources of potential bias or imprecision. Discuss both direction and magnitude of any potential bias	9, 10
Interpretation	20	Give a cautious overall interpretation of results considering objectives, limitations, multiplicity of analyses, results from similar studies, and other relevant evidence	8-10
Generalisability	21	Discuss the generalisability (external validity) of the study results	10
**Other information**			
Funding	22	Give the source of funding and the role of the funders for the present study and, if applicable, for the original study on which the present article is based	10

*Give information separately for cases and controls in case-control studies and, if applicable, for exposed and unexposed groups in cohort and cross-sectional studies.

Note: An Explanation and Elaboration article discusses each checklist item and gives methodological background and published examples of transparent reporting. The STROBE checklist is best used in conjunction with this article (freely available on the Web sites of PLoS Medicine at http://www.plosmedicine.org/, Annals of Internal Medicine at http://www.annals.org/, and Epidemiology at http://www.epidem.com/). Information on the STROBE Initiative is available at www.strobe-statement.org.

For peer review only

BMJ Open

Identical anthropometric characteristics of impaired fasting glucose combined with impaired glucose tolerance and newly-diagnosed type 2 diabetes: anthropometric indicators to predict hyperglycemia in a community-based prospective cohort study in southwest China

Journal:	*BMJ Open*
Manuscript ID	bmjopen-2017-019735.R1
Article Type:	Research
Date Submitted by the Author:	12-Dec-2017
Complete List of Authors:	Zhang, Fang; Sichuan University West China Hospital, Division of Endocrinology and Metabolism<br>Wan, Qin; Hospital affiliated to Southwest Medical University, Division of Endocrinology and Metabolism<br>Cao, Hongyi; The Fifth People's Hospital of Chengdu, Chengdu<br>Tang, Lizhi; Sichuan University West China Hospital, Division of Endocrinology and Metabolism<br>Li, Daigang; Chengdu Yinchao Community Hospital<br>Lü, Qingguo; Sichuan University West China Hospital<br>Yan, Zhe; Sichuan University West China Hospital<br>Li, Jing; Sichuan University West China Hospital<br>Yang, Qiu; The Fifth People's Hospital of Chengdu<br>Zhang, Yuwei; Sichuan University West China Hospital<br>Tong, Nanwei; Sichuan University West China Hospital, Division of Endocrinology and Metabolism
**Primary Subject Heading**:	Diabetes and endocrinology
Secondary Subject Heading:	Epidemiology, Diabetes and endocrinology
Keywords:	impaired fasting glucose combined with impaired glucose tolerance, newly-diagnosed diabetes mellitus, pre-diabetes, waist-to-height ratio, anthropometric measurements

SCHOLARONE™
Manuscripts

Title:

Identical anthropometric characteristics of impaired fasting glucose combined with impaired glucose tolerance and newly-diagnosed type 2 diabetes: anthropometric indicators to predict hyperglycemia in a community-based prospective cohort study in southwest China

Authors:

Fang Zhang 1*, Qin Wan 2*, Hongyi Cao 3*, Lizhi Tang 1, Daigang Li 4, Qingguo Lü 1, Zhe Yan 1, Jing Li 1, Qiu Yang 3, Yuwei Zhang 1, Nanwei Tong 1△

*These authors contributed equally to this paper.

△Corresponding author

Authors' affiliations:

1. Division of Endocrinology and Metabolism, West China Hospital of Sichuan University, Chengdu, Sichuan, China
2. Division of Endocrinology and Metabolism, Hospital affiliated to Southwest Medical University, Luzhou, Sichuan, China
3. Division of Endocrinology and Metabolism, The Fifth People's Hospital of Chengdu, Chengdu, Sichuan, China
4. Chengdu Yinchao Community Hospital, Chengdu, Sichuan, China

Correspondences:

Address correspondence and reprint requests to Nanwei Tong, MD, Division of Endocrinology and Metabolism, West China Hospital of Sichuan University, 37 Guoxuexiang, Chengdu, Sichuan 610041, China. Telephone number: 00+86-18980601196. Fax number: 00+86-28-85423459. E-mail: tongnw@scu.edu.cn.

For peer review only

Abstract

Objectives: To assess the anthropometric characteristics of normoglycemic individuals who subsequently developed hyperglycemia, and to evaluate the validity of these measures to predict pre-diabetes and diabetes.

Design: A community-based prospective cohort study.

Participants: In total, 1885 residents with euglycemia from six communities were enrolled.

Setting: Sichuan, southwest China

Primary outcome measures: The incidences of pre-diabetes and diabetes were the primary outcomes.

Methods: The waist-to-height ratio (WHtR), body mass index (BMI), waist circumference (WC), and waist-to-hip ratio (WHR) of all participants were measured at baseline and during follow-up. A 75 g glucose oral glucose tolerance test was conducted at each survey.

Results: During a median of 3.00 (interquartile range: 2.92—4.17) years follow-up, the cumulative incidence of isolated impaired fasting glucose (IFG), isolated impaired glucose tolerance (IGT), IFG combined with IGT (IFG+IGT), and newly-diagnosed diabetes mellitus (NDDM) were 8.44%, 18.14%, 8.06%, and 13.79%, respectively. WHtR, BMI, WC, and WHR were significantly different among subjects who subsequently progressed to isolated IFG or IGT, IFG+IGT, or NDDM ($P < 0.05$). The anthropometric characteristics of IFG+IGT subjects were similar to those of the NDDM population ($P > 0.005$). All the baseline anthropometric measurements were useful for the prediction of future pre-diabetes and NDDM ($P < 0.05$). The optimal thresholds for the four measurements were calculated for the prediction of hyperglycemia, with a WHtR value of 0.52 performing best to identify isolated IFG or IGT, IFG+IGT, and NDDM.

Conclusions: Anthropometric measures, especially WHtR, could be used to predict hyperglycemia 3 years in advance. Distinct from isolated IFG and IGT, the individuals who developed combined IFG+IGT had identical anthropometric profiles to those who progressed to NDDM.

Key words: anthropometric measurements, impaired fasting glucose combined with impaired glucose tolerance, newly-diagnosed diabetes mellitus, pre-diabetes, waist-to-height ratio

For peer review only

Strengths and limitations of this study

1. This study described and compared the anthropometric characteristics of participants who subsequently progressed to isolated impaired fasting glucose (IFG), isolated impaired glucose tolerance (IGT), IFG combined with IGT, newly-diagnosed diabetes mellitus (NDDM), or who remained normoglycemic.
2. Variations in waist-to-height ratio, body mass index, waist circumference, and waist-to-hip ratio, were used to predict the transition from euglycemia to pre-diabetes, and overt NDDM in the following 3 years.
3. The optimal threshold values for the prediction of hyperglycemia were determined from the anthropometric measurements collected.
4. The inherent limitations of the present work were a relatively short follow-up period (median 3 years), a low completion ratio of 41.9%, and a limited sample size, meaning that anthropometric threshold values could not be determined by gender for each category of hyperglycemia.

For peer review only

Introduction

The rapidly growing incidence of diabetes means that, it is now reaching epidemic proportions in China. The overall prevalences of diabetes and pre-diabetes were estimated to be 11.6% and 50.1%, respectively, in Chinese adults in 2010 [1]. In 2007—2008, another cross-sectional study conducted across China found that the prevalences of isolated impaired fasting glucose (IFG), isolated impaired glucose tolerance (IGT), and IFG combined with IGT (IFG+IGT), were 3.2%, 11.0%, and 1.9% in men, and 2.2%, 10.9%, and 1.7% in women, respectively [2]. Isolated IFG, isolated IGT, and IFG+IGT, were selected as three different categories of pre-diabetes, reflecting the progression from euglycemia to type 2 diabetes (T2D). Approximately 75%—80% of diabetes patients develop cardiovascular disease (CVD) ultimately, and patients with pre-diabetes have also been shown to be at greater risk of heart attack and stroke [3-5]. It has been estimated that between 2005 and 2015, diabetes and consequent CVD have cost China US$ 557.7 billion [6].

Measures to limit pre-diabetes are critical for the prevention of diabetes. Early recognition of pre-diabetes and prompt intervention could also reduce the impact on society as a whole. Both overall and central adiposity are closely linked to hyperglycemia. Body mass index (BMI) correlates with overall adiposity, while waist circumference (WC), waist-to-height ratio (WHtR), and waist-to-hip ratio (WHR) are indicators of central obesity. These four anthropometric indices are used globally to assess the risk of current or future diabetes [7-9].

Anthropometry is an affordable and practical screening tool for the presence of hyperglycemia, in both wealthy and impoverished areas of China. In this community-based prospective cohort study, we aimed to determine whether these anthropometric indices could predict future pre-diabetes and diabetes, and to establish optimal threshold values for the population. The baseline anthropometric characteristics of normoglycemic subjects, who subsequently developed isolated IFG, isolated IGT, IFG+IGT, and newly-diagnosed diabetes mellitus (NDDM) during follow-up, were compared and the similarities and differences between pairs of hyperglycemic categories were analyzed.

Study design and methods

Study population

The present study included two populations, in Luzhou City and in the Wenjiang area of Chengdu City. The Luzhou population are participants in the Risk Evaluation of cAncers in Chinese diabeTic Individuals: a lONgitudinal (REACTION) study, which is multicenter prospective observational study of 25 communities in mainland China [10, 11]. A total of 10007 residents, aged 40—89 years, were randomly recruited to participate in this study from five communities in Luzhou in 2011. Subjects with a history of diabetes, incident diabetes, or pre-diabetes, verified by an oral glucose tolerance test (OGTT), those missing values or any parameter, or having any of the other conditions (listed below), were excluded. After this, 3800 individuals with normoglycemia remained to form the baseline population. Of these, 1354 participants returned to complete the study in 2014. In addition, in 2016, 228 members of the baseline normoglycemic population who had not been studied in 2014, were followed up. Therefore, data from a total of 1582 subjects from Luzhou baseline screen were available

For peer review only

for analysis.

In the Wenjiang survey, a cohort of 1104 participants aged 40—75 years were randomly recruited from Yinchao community in 2011. Using the same inclusion criteria, 698 normoglycemic individuals comprised the baseline population. Of these, 303 subjects were followed up in 2015 and completed the study. Thus, from Luzhou and Wenjiang, a total of 1885 participants were included in the analysis.

All of the subjects were of Han Chinese ethnicity. A flow diagram of the study design is displayed as Supplemental Figure 1. Individuals with the following conditions were excluded from the study: infection, pregnancy, malignant tumor, acute cardiovascular accident, serious trauma, liver or renal dysfunction, or long history of glucocorticoid use. The research was conducted in accordance with the principles of the Declaration of Helsinki II. All protocols used in this work were approved either by the Medical Ethics Committee of the hospital affiliated to the Southwest Medical University in Luzhou, or by the Committee on Human Research at the Fifth People's Hospital of Chengdu in Wenjiang. Each participant provided written informed consent.

Diagnosis of diabetes and pre-diabetes

The diagnosis of hyperglycemic disorder was made in accordance with the American Diabetes Association recommendations, using OGTT, in 2011 [12]. Normal glycemic tolerance (NGT) was defined by a fasting plasma glucose (FPG) < 5.6 mmol/L and a 2-hour plasma glucose (2hPG) < 7.8 mmol/L. Isolated IFG was defined by 5.6 mmol/L ≤ FPG < 7.0 mmol/L and a 2hPG < 7.8 mmol/L, while isolated IGT was defined by an FPG < 5.6 mmol/L and 7.8 mmol/L ≤ 2hPG < 11.1 mmol/L. IFG+IGT was defined by 5.6 mmol/L ≤ FPG < 7.0 mmol/L and 7.8 mmol/L ≤ 2hPG < 11.1 mmol/L. Diabetes was defined by an FPG ≥ 7.0 mmol/L and/or a 2hPG ≥ 11.1 mmol/L.

Anthropometric measurements

Anthropometric measurements, including body mass, height, WC, and hip circumference were made by trained investigators. Measurements were conducted while all participants were wearing light clothing, without footwear after a 10—12 hour overnight fast in the morning. Measurements were made using calibrated weighing scales, standard steel strip stadiometers, and tape measures. The results were recorded to the nearest 0.1 kg or 0.1 cm. WC was measured at the midpoint between the costal border and the iliac crest at the end of exhalation. Hip circumference was measured around the widest portion of the buttocks. BMI was calculated as body mass (kg) divided by height squared (m^2), WHtR was calculated as WC (cm) divided by height (cm), and WHR as WC (cm) divided by hip circumference (cm).

Lifestyle variables and biological evaluation

Trained investigators collected lifestyle information, consisting of demographic characteristics, current smoking status, physical activity situation, medications, and personal and family disease histories, using a standard questionnaire and face-to-face interviews. The questionnaire categorized the participants into two groups: subjects undertaking vigorous

For peer review only

physical activity ≥ 1 day per week and subjects undertaking vigorous physical activity on < 1 day per week. Blood pressure (BP) was measured three times in each participant using an electronic sphygmomanometer (OMRON, HEM-7220, Liaoning, China), with 5 min intervals between measurements, after at least 10 min rest, and the mean value was recorded.

All participants underwent an OGTT. After a 10—12 hour overnight fast, venous blood was drawn both before and 2 hours after they drank 300 ml water containing 75 g anhydrous glucose within 5 min. FPG and 2hPG concentrations were measured within 24 hours using the hexokinase method (Hitachi 7600 automatic biochemical analyzer, Hitachi Ltd., Tokyo, Japan). Fasting blood samples were collected for lipid profile measurements, including total cholesterol (TC), triglyceride (TG), high-density lipoprotein cholesterol (HDL-c), and low-density lipoprotein cholesterol (LDL-c). Serum TC, TG, and HDL-c concentrations were measured using oxidase colorimetric methods, and LDL-c concentration was measured by homogeneous assay, on a Hitachi 7600 automatic biochemical analyzer (Hitachi Ltd., Tokyo, Japan) within 24 hours. Hemoglobin A1c (HbA1c) was measured using the high performance liquid chromatography (VARIANT™ II TURBO Hemoglobin Testing System, Bio-Rad Laboratories, CA, USA). The samples were stored at − 20°C until analysis, which was undertaken within 3 weeks.

Statistical analysis

Data were analyzed using SPSS software version 16.0 (SPSS, Chicago, IL, USA) and MedCalc software version 15.2.2 (MedCalc software, Ostend, Belgium). All data are expressed as mean ± SD, median (interquartile range), or frequency (%), as appropriate. One-way ANOVA was used for parametric data, whereas the rank sum test was applied for non-parametric data. The chi-square test was used for the comparison of ratio. All tests were two-sided. In analyses of more than three groups, overall $P < 0.05$ was considered significant. The Bonferroni correction and chi-square segmentation were used for multiple comparison adjustments. For the comparison of two specific subgroups, $P < 0.005$ was considered significant. For BMI, WHtR, WC, and WHR, receiver operating characteristic (ROC) curve analyses were used to compare their ability to predict incident pre-diabetes and diabetes. The non-parametric approach described by DeLong *et al*. was used to compare the areas under ROC curves [13]. The predictive threshold values for hyperglycemia were calculated. COX proportional hazards regression was used to evaluate associations between anthropometric indices and hyperglycemic categories; the time axis consisted of the period of follow-up until pre-diabetes or diabetes developed, or the end of the study. Hazard ratio (HR) and 95% confidence interval (CI) were calculated.

Results

Characteristics of subjects at baseline

A total of 1885 normoglycemic subjects (649 men and 1236 women), with a median age of 56 (interquartile range: 48—61) years old, were recruited in 2011. After a median follow-up of 3.00 (2.92—4.17) years, 159 individuals had developed isolated IFG, 342 had developed isolated IGT, 152 had developed IFG+IGT, 260 had developed NDDM, and the remaining 972 participants remained normoglycemic. The incidences of pre-diabetes and NDDM were

For peer review only - http://bmjopen.bmj.com/site/about/guidelines.xhtml

calculated to be 104.9 per 1000 person-years and 41.8 per 1000 person-years, respectively. The characteristics of all the subjects at baseline in Luzhou and Wenjiang are shown in Supplemental Table 1. The participants in Luzhou were older than the participants in Wenjiang, and had higher glucose levels at baseline and greater incidences of pre-diabetes and diabetes during follow-up. The baseline measurements of the participants who subsequently developed isolated IFG, isolated IGT, IFG+IGT, or NDDM in the future, are shown in Table 1. The subjects who developed NDDM were the oldest group at baseline of the five groups (P = 0.000). The individuals who transited to isolated IGT, IFG+IGT, or NDDM had higher baseline HbA1c levels than the subjects who remained normoglycemic ($P < 0.005$).

Baseline and follow-up anthropometric values in subjects who subsequently developed hyperglycemic disorders

During the follow-up examination, it was found that WHtR in the NGT group was lower than in the isolated IGT, IFG+IGT, or NDDM groups ($P < 0.005$) (Table 2), and was lower in the isolated IFG and isolated IGT groups than in the IFG+IGT and NDDM groups ($P < 0.005$). The P values were 0.009 and 0.006 for BMI in isolated IFG versus IFG+IGT, and isolated IGT versus IFG+IGT, respectively, and 0.005 for WHR in the isolated IFG or IGT groups versus the IFG+IGT group. There were the trends towards the differences in both BMI and WHR between the isolated IFG or IGT groups, and the IFG+IGT group. To summarize, BMI, WC, and WHR in the five hyperglycemic groups tended to follow the following pattern: NGT < isolated IFG and isolated IGT < IFG+IGT and NDDM. Unlike when the isolated IFG or isolated IGT groups were compared, the anthropometric characteristics of the IFG+IGT group were similar to those of the NDDM at follow-up ($P > 0.005$).

To assess whether the anthropometric values were already different before hyperglycemia developed, we evaluated the differences between groups at baseline, when all the subjects were still normoglycemic. Baseline WHtR, BMI, and WHR, but not WC, substantially differed among the five groups ($P < 0.05$) (Table 2). NGT subjects had lower WHtR than the subjects who subsequently developed hyperglycemia ($P < 0.005$). The WHtR values of the IFG+IGT and NDDM groups were higher than those of the isolated IFG group ($P < 0.005$), while the isolated IGT group had a lower WHtR than the NDDM group ($P < 0.005$). The BMI of the NGT group was lower than those of the isolated IGT, IFG+IGT, and NDDM groups ($P < 0.005$), and the isolated IFG group had a lower BMI than NDDM subjects ($P < 0.005$). In addition, NGT individuals had a lower WHR than NDDM patients at baseline ($P < 0.005$). Consistent with the findings at follow-up, it is worth noting that at baseline, there were no significant differences in WHtR, BMI, and WHR between individuals who subsequently developed IFG+IGT and those who converted to NDDM ($P > 0.005$).

Use of baseline anthropometric indices to predict future pre-diabetes and NDDM

For the prediction of isolated IFG, baseline WHtR, WC, and WHR showed significantly different areas under the curve (AUCs) ($P < 0.05$) (Table 3). WHtR and WC were more effective at predicting isolated IFG than BMI ($P < 0.05$) (Figure 1A). For subjects who developed isolated IGT, the AUCs of all the four indices were significant (P = 0.000). WHtR had a higher predictive value than BMI, WC, and WHR ($P < 0.05$), while WC was superior to

For peer review only - http://bmjopen.bmj.com/site/about/guidelines.xhtml

WHR for predicting isolated IGT ($P < 0.05$) (Figure 1B). For IFG+IGT incidence, all four parameters were valuable predictors ($P = 0.000$), among which WHtR and WC ranked higher than WHR ($P < 0.05$) (Figure 1C). For the prediction of NDDM, the four indices were significant ($P < 0.05$), but WHtR was the best predictor ($P < 0.05$) (Figure 1D). The optimal thresholds for predicting hyperglycemia for the four indices (WC and WHR thresholds for men and women) were then calculated.

Multivariate analysis of baseline anthropometric indices with respect to risk of subsequent pre-diabetes and NDDM

According to COX proportional hazards regression, the risk of developing isolated IFG was greater with higher WC at baseline ($P < 0.05$) (Table 4). The risk factors for the development of isolated IGT were baseline WHtR, BMI, and WC ($P < 0.05$). For both IFG+IGT and NDDM, high baseline WHtR, BMI, WC, and WHR were all risk factors ($P < 0.05$).

Discussion

In this community-based prospective cohort study, we have shown that: (1) For patients with hyperglycemia, WHtR, BMI, WC, and WHR tended to be as follows: NGT < isolated IFG and isolated IGT < IFG+IGT and NDDM. (2) Among these categories of hyperglycemia, it is noteworthy that unlike with respect to isolated IFG and isolated IGT, there were no significant differences in baseline WHtR or BMI between subjects with IFG+IGT and NDDM. (3) Thus, WHtR, BMI, WC, and WHR could predict the presence of pre-diabetes or diabetes 3 years in advance. Furthermore, the greater were these baseline anthropometric values, the higher was the risk of developing hyperglycemia. (4) Optimal threshold values for the four variables for identification of pre-diabetes and diabetes were calculated, with WHtR performing best of these in the prediction of hyperglycemia.

An Iranian study of 5879 people 9 years after they were initially found to be normoglycemic, found that 1755 subjects had developed pre-diabetes, and that isolated IFG was the commonest pre-diabetic phenotype. This study found that among women, in contrast to the use of BMI, hip and waist circumferences, WHtR was the only significant anthropometric predictor of pre-diabetes [14]. Lyssenko *et al.* reported a study of 1190 subjects in Finland who initially had NGT. During a median follow-up of 6 years, 199 had progressed to pre-diabetes. Compared with those who remained NGT, those with pre-diabetes had substantially higher BMI and WHtR at baseline [15]. Many investigators have shown that anthropometry is tightly correlated with the occurrence of pre-diabetes, although most of the studies conducted have been cross-sectional, rather than longitudinal [16-19].

After reviewing the literature, we found some common themes: (1) With respect to pre-diabetes, the majority of the studies only defined one or two distinct pre-diabetic phenotypes, or defined a single category called "pre-diabetes". (2) Rarely did investigators describe the respective anthropometric characteristics of the various hyperglycemic disorders in their manuscripts. We located only one previous report that gave anthropometric information in detail for all the potential pre-diabetic phenotypes and NDDM [20]. It was shown in this study that WHtR, BMI, WC, and WHR varied substantially among subjects

with NGT, isolated IFG, isolated IGT, IFG+IGT, and NDDM, but none of the anthropometric indices were compared between hyperglycemic groups. Therefore, the possibility that anthropometry might vary between pre-diabetes and NDDM could not be assessed, and moreover, this study was cross-sectional. To our knowledge, the present work is the first prospective cohort study that not only described the anthropometric characteristics of participants who progressed to diverse hyperglycemic conditions, but also demonstrated the variation among WHtR, BMI, WC, and WHR in the transition from NGT to pre-diabetes and overt NDDM.

The pathogenesis of isolated IFG and isolated IGT is heterogeneous, while individuals with IFG+IGT manifest both hepatic and peripheral insulin resistance. Pre-diabetes, as an intermediate hyperglycemic state, carries a high-risk for the subsequent development of diabetes. Among the three pre-diabetic phenotypes, IFG+IGT carries approximately twice the risk of transition to diabetes compared with subjects with just one of abnormalities [21]. In our previous work, we found that several biomarkers in individuals with IFG+IGT had similar values to those present in the NDDM population, but these were different in individuals with IFG or IGT alone [22-24]. Consistent with this, in the present study we observed that participants who subsequently developed hyperglycemia had higher WHtR, BMI, and WHR at baseline than those who remained NGT. Among the three pre-diabetic phenotypes, IFG+IGT subjects had the most adverse anthropometric profiles at baseline, such that there were no significant differences from the NDDM group. These findings may imply that although IFG+IGT is a subtype of pre-diabetes, some aspects of its pathophysiology have already deteriorated to the same extent as in NDDM. However, pre-diabetes is a reversible condition and consequently, prompt intervention is required to avoid or delay its progression, especially for patients with IFG+IGT.

A prospective study conducted in Pima Indians found that BMI and WHtR were the best predictors of diabetes in men, while BMI, WHtR, WC, and waist-to-thigh ratio were the best predictors in women [25]. Chei *et al.* published a cohort study of 5617 Japanese participants, finding that in women only, the significant predictors of T2D were BMI, WC, and WHtR [26]. Finally, in a multi-ethnic cohort of 1073 non-Hispanic white, Hispanic, and African American non-diabetic individuals, baseline anthropometric information showed that BMI was most predictive of diabetes in the non-Hispanic white and Hispanic populations, whereas all the indicators of central obesity were more predictive than measures of overall adiposity in the African American population [27]. The contrasts in these sets of data indicate that the validity of such anthropometric measurements for the prediction of diabetes development vary among different ethnicities, genders, and regions. Based on our ROC analysis, WHtR was most effective for the prediction of pre-diabetes and overt NDDM, followed by WC, while BMI and WHR were relatively weak predictors. Results from two western Pacific studies were consistent with our findings [28, 29].

A systematic review proposed that the threshold values for WHtR in the prediction of diabetes in men and women are 0.52 and 0.53, respectively [30]. In a Chinese community-based prospective cohort study, the optimal threshold values for WHtR and BMI were 0.51 and 24

For peer review only

for men, and 0.55 and 25 for women, respectively [29]. These predictive values were similar to those identified in our study.

Several limitations to our work should be addressed. First, the follow-up period of a median 3.00 years was relatively short. However, we identified high cumulative incidences of pre-diabetes and NDDM (34.6% and 13.8%, respectively). The fast pace of life and sedentary lifestyle of the population may be the main contributor to the rapid growth in hyperglycemia. However, it might also be the result of selection bias, because subjects with a higher risk might be more likely to take part in the follow-up assessment. In addition, the participants were ≥ 40 years old, a little older than the subjects (≥ 35 years) in some other epidemiological studies. This might be also an explanation that a large proportion of subjects became hyperglycemic in this cohort study. Second, the proportion of participants attending the follow-up assessment was low (41.91%). Conducting of a phone interview once a year at least, followed by prompt examination, could improve this statistic in the future. Third, the sample size was limited. On account of this weakness, it was not possible to calculate anthropometric threshold values for each hyperglycemic state by gender. Further studies are required to establish specific screening thresholds for pre-diabetes and NDDM in men and women, especially with regard to WC and WHR. Fourth, there was lack of OGTT reproducibility in each set of measurements. Unwillingness of subjects, and limited staff and financial resources, were the two major causes of this. By combining these data with the questionnaire data and the HbA1c results, we tried to minimize the associated error and improve the diagnostic accuracy as much as possible.

In summary, WHtR, BMI, WC, and WHR are all predictors of the development of pre-diabetes and NDDM 3 years in advance. Individuals with high WHtR, BMI, WC, and WHR are thus at higher risk of developing pre-diabetes and T2D. The optimal thresholds for all the anthropometric measures to predict hyperglycemia were calculated, with a WHtR value of 0.52 performing best at predicting the development of isolated IFG or IGT, IFG+IGT, and NDDM. The magnitude of WHtR and BMI in normoglycemic subjects illustrate the likelihood of progression from normoglycemia to pre-diabetes, and then to overt T2D. Of note, and in contrast to the situation with regard to isolated IFG or IGT, the anthropometric characteristics of IFG+IGT subjects were similar to those of the NDDM population, both at baseline and follow-up.

Contributorship statement

All the authors engaged in the surveys. FZ and NT designed this article. QW, HC, DL and QY acquired and collected data. JL, ZY, QL and YZ organized all the data. FZ, QW and HC analyzed all the information. FZ and LT drafted the manuscript. FZ and NT revised the article critically. All the authors read and approved the final manuscript.

For peer review only

Acknowledgements

All the authors appreciated the staffs from primary health care centers for materials supplement and participant organization.

Competing interests

All the authors declared that there were no competing interests among them.

Fundings

The surveys in Luzhou were supported by the grants from the National Clinical Research Center for Metabolic Diseases (2013BAI09B13), the National Key New Drug Creation and Manufacturing Program of Ministry of Science and Technology (2012ZX09303006—001). The screenings in Wenjiang were supported by the National Key Technology R&D Program of China (Grant No. 2009BAI80B01 and No. 2009BAI80B02) and Sichuan Provincial Science and Technology Foundation (Grant No. 150022 and No. 2015SZ0228).

Data sharing statement

A supplementary profile will be available online which contains comprehensive figure and table of used input data. Inquiries about additional unpublished data could be contacted with the corresponding author.

References

1. Xu Y, Wang L, He J, Bi Y, Li M, Wang T, Wang L, Jiang Y, Dai M, Lu J, Xu M, Li Y, Hu N, Li J, Mi S, Chen CS, Li G, Mu Y, Zhao J, Kong L, Chen J, Lai S, Wang W, Zhao W, Ning G; 2010 China Noncommunicable Disease Surveillance Group. Prevalence and Control of Diabetes in Chinese Adults. *JAMA*. 2013; 310 (9): 948-958.
2. Yang W, Lu J, Weng J, Jia W, Ji L, Xiao J, Shan Z, Liu J, Tian H, Ji Q, Zhu D, Ge J, Lin L, Chen L, Guo X, Zhao Z, Li Q, Zhou Z, Shan G, He J; China National Diabetes and Metabolic Disorders Study Group. Prevalence of Diabetes among Men and Women in China. *N Engl J Med.* 2010; 362: 1090-101.
3. Rydén L, Grant PJ, Anker SD, Berne C, Cosentino F, Danchin N, Deaton C, Escaned J, Hammes HP, Huikuri H, Marre M,Marx N, Mellbin L, Ostergren J, Patrono C, Seferovic P, Uva MS, Taskinen MR, Tendera M, Tuomilehto J, Valensi P, Zamorano JL; ESC Committee for Practice Guidelines (CPG), Zamorano JL, Achenbach S, Baumgartner H, Bax JJ, Bueno H, Dean V, Deaton C, Erol C, Fagard R, Ferrari R, Hasdai D, Hoes AW, Kirchhof P, Knuuti J, Kolh P, Lancellotti P, Linhart A, Nihoyannopoulos P, Piepoli MF, Ponikowski P, Sirnes PA, Tamargo JL, Tendera M, Torbicki A,Wijns W, Windecker S; Document Reviewers, De Backer G, Sirnes PA, Ezquerra EA, Avogaro A, Badimon L, Baranova E, Baumgartner H, Betteridge

J,Ceriello A, Fagard R, Funck-Brentano C, Gulba DC, Hasdai D, Hoes AW, Kjekshus JK, Knuuti J, Kolh P, Lev E, Mueller C, Neyses L, Nilsson PM, Perk J,Ponikowski P, Reiner Z, Sattar N, Schächinger V, Scheen A, Schirmer H, Strömberg A, Sudzhaeva S, Tamargo JL, Viigimaa M, Vlachopoulos C, Xuereb RG. ESC Guidelines on diabetes, pre-diabetes, and cardiovascular diseases developed in collaboration with the EASD. *European Heart Journal.* 2013; 34: 3035–3087.

4. Tanne D, Koren-Morag N, Goldbourt U. Fasting plasma glucose and risk of incident ischemic stroke or transient ischemic attacks: a prospective cohort study. *Stroke.* 2004; 35:2351-5.
5. Norhammar A, Tenerz A, Nilsson G, Hamsten A, Efendíc S, Rydén L, Malmberg K. Glucose metabolism in patients with acute myocardial infarction and no previous diagnosis of diabetes mellitus: a prospective study. *Lancet.* 2002; 359: 2140-4.
6. Chinese Diabetes Society. China Guideline for Type 2 Diabetes. *Chinese Diabetes Society.* 2010. (Chinese)
7. Chen L, Magliano DJ, Balkau B, Colagiuri S, Zimmet PZ, Tonkin AM, Mitchell P, Phillips PJ, Shaw JE. AUSDRISK: an Australian type 2 diabetes risk assessment tool based on demographic, lifestyle and simple anthropometric measures. *Medical Journal of Australia.* 2010; 192 (4): 197–202.
8. Lindstrom J, Tuomilehto J. The diabetes risk score: a practical tool to predict type 2 diabetes risk. *Diabetes Care.* 2003; 26: (3)725–731.
9. Mohan V, Sandeep S, Deepa M, Gokulakrishnan K, Datta M, Deepa R. A diabetes risk score helps identify metabolic syndrome and cardiovascular risk in Indians – the Chennai Urban Rural Epidemiology Study (CURES-38). *Diabetes, Obesity and Metabolism.* 2007; 9 (3): 337–343.
10. Lu J, Bi Y, Wang T, Wang W, Mu Y, Zhao J, Liu C, Chen L, Shi L, Li Q, Wan Q, Wu S, Qin G, Yang T, Yan L, Liu Y, Wang G, Luo Z, Tang X, Chen G, Huo Y, Gao Z, Su Q, Ye Z, Wang Y, Deng H, Yu X, Shen F, Chen L, Zhao L, Dai M, Xu M, Xu Y, Chen Y, Lai S, Ning G. The relationship between insulin-sensitive obesity and cardiovascular diseases in a Chinese population: Results of the REACTION study. *International Journal of Cardiology.* 2014; 172: 388–394.
11. Bi Y, Lu J, Wang W, Mu Y, Zhao J, Liu C, Chen L, Shi L, Li Q, Wan Q, Wu S, Yang T, Yan L, Liu Y, Wang G, Luo Z, Tang X, Chen G, Huo Y, Gao Z, Su Q, Ye Z,Wang Y, Qin G, Deng H, Yu X, Shen F, Chen L, Zhao L, Zhang J, Sun J, Dai M, Xu M, Xu Y, Chen Y, Lai S, Bloomgarden ZT, Li D, Ning G. Cohort profile: Risk evaluation of cancers in Chinese diabetic individuals: A longitudinal (REACTION) study. *J Diabetes.* 2014; 6 (2): 147–57.
12. American Diabetes Association. Standards of medical care in diabetes–2010. *Diabetes Care.* 2011; 33 (Suppl. 1): S11–61.
13. DeLong ER, DeLong DM, Clarke-Pearson DL. Comparing the areas under two or more correlated receiver operating characteristic curves: a nonparametric approach. *Biometrics.* 1988; 44 (3): 837-45.
14. Hadaegh F, Derakhshan A, Zafari N, Khalili D, Mirbolouk M, Saadat N, Azizi F. Pre-diabetes tsunami: incidence rates and risk factors of pre-diabetes and its different phenotypes over 9 years of follow-up. *Diabet Med.* 2017; 34 (1): 69-78.

15. Lyssenko V, Almgren P, Anevski D, Perfekt R, Lahti K, Nissén M, Isomaa B, Forsen B, Homström N, Saloranta C, Taskinen MR, Groop L, Tuomi T, Botnia study group. Predictors of and longitudinal changes in insulin sensitivity and secretion preceding onset of type 2 diabetes. *Diabetes.* 2005; 54 (1):166-74.

16. Xu Z, Qi X, Dahl AK, Xu W. Waist-to-height ratio is the best indicator for undiagnosed type 2 diabetes. *Diabet Med*. 2013; 30 (6):e201-7.

17. Hu D, Xie J, Fu P, Zhou J, Yu D, Whelton PK, He J, Gu D. Central rather than overall obesity is related to diabetes in the Chinese population: the InterASIA study. *Obesity (Silver Spring)*. 2007 Nov; 15 (11):2809-16.

18. Jung SH, Ha KH, Kim DJ. Visceral fat mass has stronger associations with diabetes and prediabetes than other anthropometric obesity indicators among Korean adults. *Yonsei Med J.* 2016 May; 57 (3):674-80.

19. Hayashi T, Boyko EJ, Leonetti DL, McNeely MJ, Newell-Morris L, Kahn SE, Fujimoto WY. Visceral adiposity and the risk of impaired glucose tolerance: a prospective study among Japanese Americans. *Diabetes Care*. 2003; 26 (3):650-5.

20. Alperet DJ, Lim WY, Mok-Kwee Heng D, Ma S, van Dam RM. Optimal anthropometric measures and thresholds to identify undiagnosed type 2 diabetes in three major Asian ethnic groups. *Obesity (Silver Spring).* 2016 Oct; 24 (10):2185-93.

21. Nathan DM, Davidson MB, DeFronzo RA, Heine RJ, Henry RR, Pratley R, Zinman B, American Diabetes Association. Impaired fasting glucose and impaired glucose tolerance: implications for care. *Diabetes Care*. 2007 Mar; 30 (3):753-9.

22. Huang L, Tong Y, Zhang F, Yang Q, Li D, Xie S, Li Y, Cao H, Tang L, Zhang X, Tong N. Increased acyl ghrelin but decreased total ghrelin and unacyl ghrelin in Chinese Han people with impaired fasting glucose combined with impaired glucose tolerance. *Peptides*. 2014 Oct; 60:86-94.

23. Lü Q, Tong N, Liu Y, Li N, Tang X, Zhao J, Cao H, Li D, Gou L, Zhang Y, Wan J, Jiang L. Community-based population data indicates the significant alterations of insulinresistance , chronic inflammation and urine ACR in IFG combined IGT group amongprediabetic pop ulation. *Diabetes Res Clin Pract*. 2009 Jun; 84 (3):319-24.

24. Zhang F, Tang X, Cao H, Lü Q, Li N, Liu Y, Zhang X, Zhang Y, Cao M, Wan J, An Z, Tong N. Impaired secretion of total glucagon-like peptide-1 in people with impaired fasting glucose combined impaired glucose tolerance. *Int J Med Sci.* 2012; 9 (7):574-81.

25. Tulloch-Reid MK, Williams DE, Looker HC, Hanson RL, Knowler WC. Do measures of body fat distribution provide information on the risk of type 2 diabetes in addition to measures of general obesity? Comparison of anthropometric predictors of type 2 diabetes in Pima Indians. *Diabetes Care*. 2003 Sep; 26 (9):2556-61.

26. Chei CL, Iso H, Yamagishi K, Tanigawa T, Cui R, Imano H, Kiyama M, Kitamura A, Sato S, Shimamoto T. Body fat distribution and the risk of hypertension and diabetes among Japanese men and women. *Hypertens Res.* 2008 May; 31 (5):851-7.

27. MacKay MF, Haffner SM, Wagenknecht LE, D'Agostino RB Jr, Hanley AJ. Prediction of type 2 diabetes using alternate anthropometric measures in a multi-ethnic

cohort: the insulin resistance atherosclerosis study. *Diabetes Care.* 2009 May; 32 (5):956-8.

28. Son YJ, Kim J, Park HJ, Park SE, Park CY, Lee WY, Oh KW, Park SW, Rhee EJ. Association of Waist-Height Ratio with Diabetes Risk: A 4-Year Longitudinal Retrospective Study. *Endocrinol Metab (Seoul).* 2016 Mar; 31 (1):127-33.

29. Xu F, Wang YF, Lu L, Liang Y, Wang Z, Hong X, Li J. Comparison of anthropometric indices of obesity in predicting subsequent risk of hyperglycemia among Chinese men and women in Mainland China. *Asia Pac J Clin Nutr.* 2010; 19 (4):586-93.

30. Browning LM, Hsieh SD, Ashwell M. A systematic review of waist-to-height ratio as a screening tool for the prediction of cardiovascular disease and diabetes: 0·5 could be a suitable global boundary value. *Nutrition Research Reviews.* 2010; 23: 247–269.

For peer review only

Table 1. General measurements of subjects at baseline who progressed to hyperglycemia at follow-up

	NGT (n = 972)	Isolated IFG (n = 159)	Isolated IGT (n = 342)	IFG+IGT (n = 152)	NDDM (n = 260)	overall *P* value
Follow-up time (year)	3.00 (2.92—4.17)‡	3.00 (2.92—4.17)‡	2.92 (2.92—3.17)*†	3.00 (2.92—3.17)	3.00 (2.92—3.17)	0.000
Age (year)	53 (46—59)†‡§¶	55 (48—62)*¶	59 (49—65)*	56 (49—62)*¶	60 (54—65)*†§	0.000
Female (N/n%)	675 (69.44%)	96 (60.38%)	220 (64.33%)	97 (63.82%)	166 (63.85%)	0.075
Height (cm)	158.00 (153.10—164.00)	159.45 (154.00—165.52)	157.00 (152.00—162.70)	157.10 (154.00—164.00)	156.00 (152.00—163.20)	0.492
Weight (kg)	58.00 (52.00—65.00)	60.50 (53.99—66.85)	60.00 (53.00—66.20)	62.10 (56.70—69.50)	62.00 (55.00—69.75)	0.498
Hip circumference (cm)	93.00 (88.20—97.20)	94.00 (90.00—99.00)	95.00 (90.20—100.00)	96.00 (92.00—100.30)	96.00 (92.00—101.00)	0.879
SBP (mmHg)	115.67 (105.33—128.67)‡§¶	118.50 (107.46—133.00)¶	122.50 (109.33—136.67)*¶	123.00 (114.00—137.67)*	130.67 (118.67—142.17)*†‡	0.000
DBP (mmHg)	74.33 (68.00—81.33)§¶	77.00 (70.00—83.75)	76.33 (69.00—83.33)¶	77.50 (72.33—82.67)*	79.00 (72.33—88.17)*‡	0.000
FPG (mmol/L)	5.08 (4.83—5.29)†§¶	5.20 (4.98—5.38)*	5.11 (4.90—5.33)	5.16 (4.93—5.36)*	5.16 (4.92—5.36)*	0.000
2hPG (mmol/L)	6.15 (5.40—6.88)‡§	6.14 (5.45—6.93)	6.40 (5.67—7.09)*	6.54 (5.85—7.10)*	6.33 (5.50—7.08)	0.000
HbA1c (%)	5.60 (5.30—5.90)‡§¶	5.70 (5.48—5.90)	5.70 (5.40—5.90)*	5.70 (5.50—6.00)*	5.70 (5.40—6.00)*	0.000
TG (mmol/L)	1.10 (0.80—1.60)	1.12 (0.80—1.63)	1.11 (0.84—1.59)	1.14 (0.89—1.60)	1.07 (0.81—1.50)	0.494
TC (mmol/L)	4.46 ± 1.01	4.45 ± 1.17	4.50 ± 1.02	4.72 ± 1.14	4.52 ± 1.10	0.062
HDL-c (mmol/L)	1.32 (1.09—1.60)	1.32 (1.05—1.52)	1.30 (1.08—1.56)	1.36 (1.20—1.57)	1.31 (1.09—1.60)	0.376
LDL-c (mmol/L)	2.51 (2.04—3.03)	2.44 (1.97—3.09)	2.53 (1.99—2.99)	2.65 (2.06—3.17)	2.45 (1.95—3.01)	0.688
Family history of diabetes (N/%)	119 (12.24%)	12 (7.55%)	29 (8.48%)	18 (11.84%)	29 (11.15%)	0.214
Current smoker (N/%)	137 (14.10%)	28 (17.61%)	42 (12.28%)	18 (11.84%)	44 (16.92%)	0.307
Physical activity (N/%)	719 (73.97%)	107 (67.30%)	255 (74.56%)	113 (74.34%)	195 (75.00%)	0.435

For peer review only

NGT, normal glucose tolerance; IFG, impaired fasting glucose; IGT, impaired glucose tolerance; IFG+IGT, IFG combined with IGT; NDDM, newly-diagnosed diabetes mellitus; SBP, systolic blood pressure; DBP, diastolic blood pressure; FPG, fasting plasma glucose; 2hPG, 2 hour plasma glucose (after oral glucose tolerance test); TG, triglyceride; TC, total cholesterol; HDL-c, high-density lipoprotein cholesterol; LDL-c, low-density lipoprotein cholesterol.

Data are expressed as means ± SD or median (interquartile range) or N (%).

Chi-square test was used to compare gender compositions, family history of diabetes, current smoking status and physical activity among five groups. If needed, chi-square segmentation was applied for further comparisons between any two subgroups with an adjusted significance level (a' = 0.005).

Kruskal-Wallis H analysis was applied for follow-up time among five groups. Mann-Whitney U analysis was performed for comparison within any two subgroups additionally (a' = 0.005).

One-way ANOVA analysis was used for the rest measurements among five groups, while LSD analysis was applied for age, SBP, DBP, FPG, 2hPG and HbA1c comparisons between any two subgroups (a' = 0.005).

*, versus NGT and $P < 0.005$; †, versus isolated IFG and $P < 0.005$; ‡, versus isolated IGT and $P < 0.005$; §, versus IFG+IGT and $P < 0.005$; ¶, versus NDDM and $P < 0.005$.

For peer review only

Table 2. Baseline and follow-up anthropometric values in participants who developed hyperglycemic disorders

	NGT (n = 972)	Isolated IFG (n = 159)	Isolated IGT (n = 342)	IFG+IGT (n = 152)	NDDM (n = 260)	overall *P* value
At follow-up survey						
WHtR (cm/cm)	0.51 (0.47—0.55)‡§¶	0.52 (0.48—0.56)§¶	0.53 (0.49—0.57)*§¶	0.54 (0.51—0.59)*†‡	0.56 (0.52—0.60)*†‡	0.000
BMI (kg/m^2)	23.46 (21.77—25.53)†‡§¶	24.27 (22.49—26.17)*¶	24.44 (22.63—26.50)*¶	25.09 (23.62—27.01)*	25.73 (23.29—27.82)*†‡	0.000
Waist circumference (cm)	80.65 (74.00—87.00)†‡§¶	82.80 (77.00—91.00)*§¶	84.00 (78.00—90.00)*§¶	86.70 (80.28—93.00)*†‡	88.00 (82.00—95.00)*†‡	0.000
WHR (cm/cm)	0.86 (0.81—0.91)†‡§¶	0.88 (0.84—0.92)*¶	0.88 (0.83—0.92)*¶	0.90 (0.86—0.94)*	0.91 (0.87—0.95)*†‡	0.000
At baseline survey						
WHtR (cm/cm)	0.50 ± 0.05†‡§¶	0.52 ± 0.06*§¶	0.53 ± 0.05*¶	0.54 ± 0.05*†	0.55 ± 0.06*†‡	0.000
BMI (kg/m^2)	23.03 (21.23—25.16)‡§¶	23.31 (21.56—25.64)¶	24.03 (22.10—26.22)*	24.98 (23.47—26.67)*	25.42 (23.17—27.22)*†	0.000
Waist circumference (cm)	79.00 (73.00—86.00)	82.00 (76.00—89.00)	83.00 (77.10—89.00)	87.00 (81.00—91.28)	86.00 (80.00—93.00)	0.282
WHR (cm/cm)	0.86 (0.81—0.90)¶	0.87 (0.92—0.92)	0.87 (0.82—0.91)	0.89 (0.86—0.93)	0.90 (0.86—0.94)*	0.010

NGT, normal glucose tolerance; IFG, impaired fasting glucose; IGT, impaired glucose tolerance; IFG+IGT, IFG combined with IGT; NDDM, newly-diagnosed diabetes mellitus; WHtR, waist-to-height ratio; BMI, body mass index; WHR, waist-to-hip ratio.

Data are expressed as median (interquartile range) or means ± SD.

At follow-up survey: One-way ANOVA analysis was used for WHtR, BMI and WC among the five glucose metabolic groups. LSD analysis was applied for the further comparisons between any two subgroups (a' = 0.005). Kruskal-Wallis H analysis was applied for WHR among the five groups and Mann-Whitney U analysis was performed for the following comparisons within any two subgroups (a' = 0.005).

At baseline survey: One-way ANOVA analysis was used for all indices among the five glucose metabolic groups. LSD analysis was applied for WHtR, BMI and WHR between any two subgroups' comparison (a' = 0.005).

*, versus NGT and $P < 0.005$; †, versus isolated IFG and $P < 0.005$; ‡, versus isolated IGT and $P < 0.005$; §, versus IFG+IGT and $P < 0.005$; ¶, versus NDDM and *P*

For peer review only

< 0.005.

For peer review only

Table 3. ROC curve analysis of baseline anthropometric indices for predicting future hyperglycemia

	AUC	SE	*P* value	95%CI	Cut-off point	Youden's value	Sensitivity	Specificity	DeLong's test (*P* value) WHtR (cm/cm)	BMI (kg/m^2)	Waist circumference (cm)	WHR (cm/cm)
Isolated IFG												
WHtR (cm/cm)	0.578	0.025	0.002	(0.529—0.626)	0.51	0.151	54.90%	60.19%	—	0.010	0.201	0.611
BMI (kg/m^2)	0.544	0.025	0.081	(0.495—0.593)	21.36	0.078	80.40%	27.43%	0.010	—	0.023	0.421
Waist circumference (cm)	0.592	0.024	0.000	(0.545—0.639)	77.10	0.148	71.24%	43.54%	0.201	0.023	—	0.195
Women	0.584	0.031	0.010	(0.524—0.644)	75.00	0.166	74.44%	42.11%	—	—	—	—
Men	0.579	0.041	0.050	(0.526—0.631)	87.00	0.165	49.21%	67.24%	—	—	—	—
WHR (cm/cm)	0.567	0.026	0.008	(0.537—0.597)	0.88	0.128	47.06%	65.71%	0.611	0.421	0.195	—
Women	0.568	0.033	0.036	(0.504—0.632)	0.85	0.140	57.78%	56.19%	—	—	—	—
Men	0.525	0.042	0.534	(0.471—0.578)	0.90	0.095	53.97%	55.52%	—	—	—	—
Isolated IGT												
WHtR (cm/cm)	0.634	0.017	0.000	(0.600—0.667)	0.51	0.214	62.24%	59.12%	—	0.003	0.006	0.000

For peer review only

BMI (kg/m^2)	0.591	0.018	0.000	(0.556—0.627)	22.68	0.155	68.88%	46.64%	0.003	—	0.178	0.223
Waist circumference (cm)	0.610	0.017	0.000	(0.576—0.645)	78.00	0.197	71.90%	47.81%	0.006	0.178	—	0.001
Women	0.635	0.021	0.000	(0.593—0.676)	78.00	0.260	68.42%	57.59%	—	—	—	—
Men	0.542	0.032	0.174	(0.480—0.605)	87.80	0.132	43.44%	69.76%	—	—	—	—
WHR (cm/cm)	0.567	0.018	0.000	(0.539—0.594)	0.86	0.123	61.63%	50.64%	0.000	0.223	0.001	—
Women	0.587	0.022	0.000	(0.544—0.630)	0.82	0.154	77.03%	38.39%	—	—	—	—
Men	0.524	0.032	0.433	(0.463—0.586)	0.89	0.098	57.38%	52.41%	—	—	—	—
IFG+IGT												
WHtR (cm/cm)	0.713	0.022	0.000	(0.670—0.755)	0.53	0.351	62.33%	72.79%	—	0.106	0.556	0.026
BMI (kg/m^2)	0.685	0.022	0.000	(0.642—0.729)	23.38	0.316	77.40%	54.22%	0.106	—	0.254	0.492
Waist circumference (cm)	0.706	0.021	0.000	(0.665—0.748)	79.80	0.351	82.88%	52.19%	0.556	0.254	—	0.032
Women	0.732	0.026	0.000	(0.682—0.783)	79.80	0.420	79.57%	62.38%	—	—	—	—
Men	0.656	0.039	0.000	(0.579—0.733)	90.30	0.242	43.40%	80.76%	—	—	—	—

For peer review only

WHR (cm/cm)	0.667	0.022	0.000	(0.638—0.695)	0.87	0.274	69.18%	58.23%	0.026	0.492	0.032	—
Women	0.686	0.027	0.000	(0.633—0.739)	0.83	0.312	86.02%	45.20%	—	—	—	—
Men	0.631	0.038	0.003	(0.556—0.705)	0.92	0.261	54.72%	71.38%	—	—	—	—
NDDM												
WHtR (cm/cm)	0.730	0.017	0.000	(0.696—0.764)	0.52	0.366	74.21%	62.43%	—	0.000	0.001	0.010
BMI (kg/m^2)	0.677	0.020	0.000	(0.639—0.716)	24.32	0.315	64.68%	66.81%	0.000	—	0.093	0.596
Waist circumference (cm)	0.700	0.018	0.000	(0.665—0.735)	78.00	0.292	81.35%	47.81%	0.001	0.093	—	0.429
Women	0.714	0.021	0.000	(0.673—0.756)	77.10	0.344	81.76%	52.63%	—	—	—	—
Men	0.686	0.033	0.000	(0.622—0.750)	88.00	0.298	56.99%	72.85%	—	—	—	—
WHR (cm/cm)	0.688	0.018	0.000	(0.661—0.715)	0.88	0.304	67.73%	62.71%	0.010	0.596	0.429	—
Women	0.696	0.022	0.000	(0.653—0.738)	0.84	0.301	79.75%	50.31%	—	—	—	—
Men	0.681	0.030	0.000	(0.622—0.740)	0.92	0.299	60.22%	69.66%	—	—	—	—

ROC, receiver operating characteristic; IFG, impaired fasting glucose; IGT, impaired glucose tolerance; IFG+IGT, IFG combined with IGT; NDDM, newly-diagnosed diabetes mellitus; AUC, area under curve; SE, standard error; CI, confidence interval; WHtR, waist-to-height ratio; BMI, body mass index; WHR,

For peer review only

waist-to-hip ratio.

For peer review only

Table 4. Multivariate analysis of baseline anthropometric indices with respect to risk of subsequent pre-diabetes and NDDM

	Isolated IFG			Isolated IGT			IFG+IGT			NDDM		
	HR	95% CI	*P*	HR	95% CI	*P*	HR	95% CI	*P*	HR	95% CI	*P*
WHtR (cm/cm)	1.471	(0.901—2.402)	0.123	1.951	(1.550—2.457)	0.000	3.002	(2.137—4.216)	0.000	2.765	(2.065—3.703)	0.000
BMI (kg/m^2)	1.186	(0.699—2.012)	0.526	1.571	(1.241—1.988)	0.000	3.298	(2.224—4.892)	0.000	2.305	(1.773—2.998)	0.000
Waist circumference (cm)	1.603	(1.112—2.310)	0.011	1.644	(1.275—2.118)	0.000	4.570	(2.948—7.084)	0.000	2.666	(1.886—3.769)	0.000
WHR (cm/cm)	1.182	(0.739—1.889)	0.486	0.972	(0.724—1.304)	0.848	1.571	(1.003—2.465)	0.048	1.706	(1.196—2.433)	0.003

NDDM, newly-diagnosed diabetes mellitus; IFG, impaired fasting glucose; IGT, impaired glucose tolerance; IFG+IGT, IFG combined with IGT; HR, hazard ratio; CI, confidence interval; WHtR, waist-to-height ratio; BMI, body mass index; WHR, waist-to-hip ratio.

Cox proportional hazards models were used to calculate HR and 95% CI. A univariate analysis was performed for each potential risk factor firstly, including age (years), gender (male/female), systolic blood pressure (mmHg), diastolic blood pressure (mmHg), fasting plasma glucose (mmol/L), 2 hour plasma glucose (mmol/L) (after oral glucose tolerance test), HbA1c (%), total cholesterol (mmol/L), triglyceride (mmol/L), high-density lipoprotein cholesterol (mmol/L), low-density lipoprotein cholesterol (mmol/L), diabetes family history (yes/no), current smoking status (yes/no), physical activity situation (yes/no), WHtR (low/high), BMI (low/high), WC (low/high) and WHR (low/high). The four anthropometric indicators were dichotomized into low or high level by using cut-off values derived from previous ROC curve analysis. Then those risk factors with a P-value < 0.2 in univariate analysis were selected to enter the multivariate model.

For peer review only

Figure legends

Figure 1. ROC curves of baseline anthropometric indices in subjects who developed (A) isolated IFG, (B) isolated IGT, (C) IFG+IGT and (D) NDDM. ROC, receiver operating characteristic; IFG, impaired fasting glucose; IGT, impaired glucose tolerance; IFG+IGT, IFG combined with IGT; NDDM, newly-diagnosed diabetes mellitus; WHtR, waist-to-height ratio; BMI, body mass index; WHR, waist-to-hip ratio; WC, waist circumference.

Supplemental Figure 1. Flow-chart of study design. NGT, normal glycemic tolerance; OGTT, oral glucose tolerance test; NDDM, newly-diagnosed diabetes mellitus. The re-visited participants in the blue background came from the baseline populations; the subjects in the pink background were the ones recruited in this study, who were normoglycemic at baseline.

For peer review only

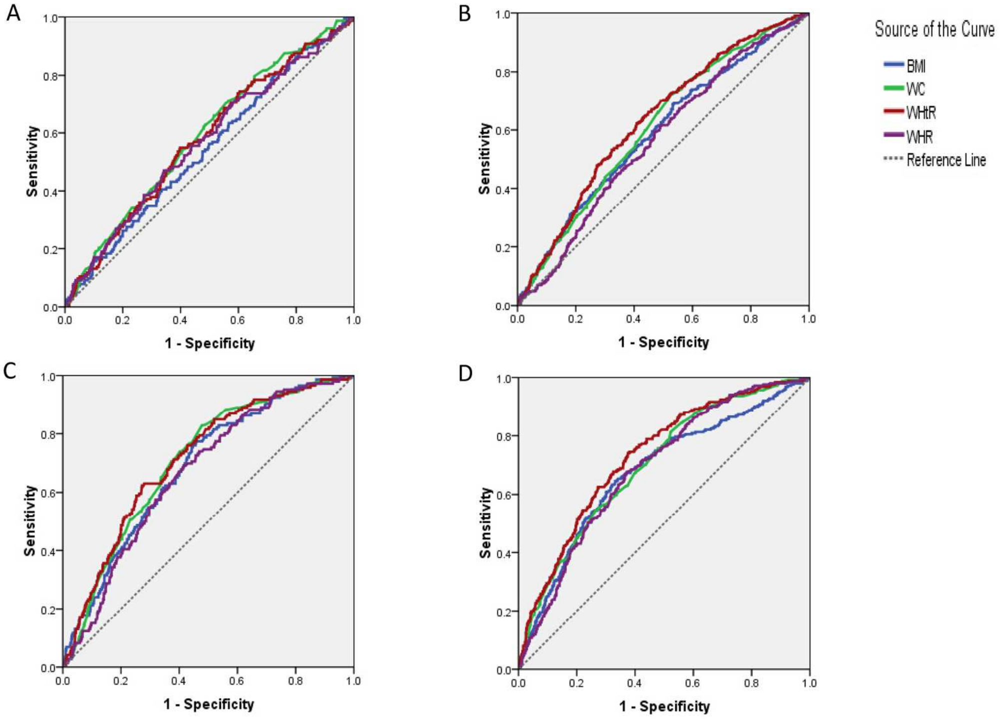

Figure 1. ROC curves of baseline anthropometric indices in subjects who developed (A) isolated IFG, (B) isolated IGT, (C) IFG+IGT and (D) NDDM. ROC, receiver operating characteristic; IFG, impaired fasting glucose; IGT, impaired glucose tolerance; IFG+IGT, IFG combined with IGT; NDDM, newly-diagnosed diabetes mellitus; WHtR, waist-to-height ratio; BMI, body mass index; WHR, waist-to-hip ratio; WC, waist circumference.

158x114mm (300 x 300 DPI)

Supplemental Table 1. Baseline characteristics of all the participants screened in Luzhou and Wenjiang

	Luzhou baseline survey			*P*	Wenjiang baseline survey			*P*
	Total (n = 1582)	Men (n = 495)	Women (n = 1087)		Total (n = 303)	Men (n = 154)	Women (n = 149)	
Age (year)	57 (50—63)	60 (54—66)	56 (49—61)	0.000	47 (43—54)	47 (43—54)	46 (42—52)	0.161
Female (N/n%)	1087 (68.71%)	—	—		149 (49.17%)	—	—	
Height (cm)	157.00 (152.40—163.00)	165.00 (160.50—169.00)	154.55 (151.00—158.20)	0.180	161.28 ±7.60	166.46 ±5.27	155.56 ±5.32	0.000
Weight (kg)	59.00 (53.00—65.30)	65.00 (58.30—72.00)	56.50 (51.20—62.50)	0.000	61.88 ±11.36	67.66 ±8.84	55.49 ±10.38	0.000
Hip circumference (cm)	94.00 (89.20—99.00)	95.00 (90.00—100.00)	94.00 (89.00—98.20)	0.655	93.47 ±6.04	95.01 ±5.43	91.76 ±6.24	0.000
SBP (mmHg)	120.67 (108.67—135.67)	126.00 (113.83—140.17)	119.00 (107.00—133.37)	0.000	114.90 ±14.27	118.20 ±14.02	111.26 ±13.69	0.000
DBP (mmHg)	75.33 (69.00—82.67)	79.00 (71.67—88.17)	74.00 (68.00—80.67)	0.000	78.40 ±16.26	81.15 ±10.84	75.36 ±20.27	0.001
FPG (mmol/L)	5.14 (4.93—5.34)	5.15 (4.96—5.38)	5.13 (4.92—5.32)	0.011	4.90 (4.60—5.10)	4.90 (4.70—5.20)	4.80 (4.60—5.10)	0.286
2hPG (mmol/L)	6.32 (5.57—7.00)	6.32 (5.57—6.98)	6.32 (5.57—7.01)	0.777	6.00 (5.00—6.70)	5.90 (5.03—5.78)	6.00 (5.00—6.80)	0.541
HbA1c (%)	5.70 (5.40—5.90)	5.70 (5.50—5.95)	5.70 (5.40—5.90)	0.069	5.48 ±0.42	5.51 ±0.38	5.45 ±0.45	0.228
TG (mmol/L)	1.33 ±0.94	1.29 ±0.84	1.34 ±0.98	0.388	1.10 (0.80—1.80)	1.50 (0.90—2.18)	0.90 (0.70—1.50)	0.000
TC (mmol/L)	4.44 (3.75—5.18)	4.32 (3.63—5.12)	4.50 (3.82—5.19)	0.017	4.53 ±0.83	4.60 ±0.82	4.45 ±0.83	0.178
HDL-c (mmol/L)	1.28 (1.06—1.52)	1.26 (1.03—1.52)	1.29 (1.08—1.52)	0.107	1.59 ±0.39	1.47 ±0.32	1.71 ±0.41	0.000
LDL-c (mmol/L)	2.52 ±0.77	2.47 ±0.74	2.54 ±0.79	0.078	2.86 ±0.75	2.92 ±0.70	2.79 ±0.80	0.252
WHtR (cm/cm)	0.52 (0.48—0.56)	0.53 (0.49—0.56)	0.52 (0.48—0.56)	0.900	0.49 (0.45—0.53)	0.50 (0.47—0.53)	0.47 (0.44—0.51)	0.000
BMI (kg/m^2)	23.74 (21.61—26.00)	24.04 (21.81—26.14)	23.68 (21.51—25.92)	0.462	23.49 (21.64—25.59)	24.40 (22.33—26.03)	22.44 (21.10—24.24)	0.000
Waist circumference (cm)	82.00 (76.00—89.00)	87.00 (80.00—92.45)	80.00 (75.00—87.10)	0.201	79.00 (72.00—86.00)	84.00 (79.00—89.00)	73.00 (69.75—78.00)	0.000
WHR (cm/cm)	0.86 (0.80—0.91)	0.89 (0.83—0.94)	0.85 (0.79—0.90)	0.476	0.86 (0.80—0.91)	0.83 (0.78—0.89)	0.87 (0.81—0.92)	0.256
Outcomes at follow-up: N/total (%)								
NGT	757 (47.85%)	203 (41.01%)	554 (50.97%)	—	215 (70.96%)	103 (66.88%)	112 (75.17%)	—
Isolated IFG	131 (8.28%)	51 (10.30%)	80 (7.34%)	—	28 (9.24%)	12 (7.79%)	16 (10.74%)	—
Isolated IGT	304 (19.22%)	103 (20.81%)	201 (18.49%)	—	38 (12.54%)	24 (15.58%)	14 (9.40%)	—
IFG+IGT	137 (8.66%)	46 (9.29%）	91 (8.37%)	—	15 (4.95%)	11 (7.14%)	4 (2.68%)	—

For peer review only

NDDM	253 (15.99%)	92 (18.59%)	161 (14.81%)	—	7 (2.31%)	4 (2.61%)	3 (2.01%)	—

SBP, systolic blood pressure; DBP, diastolic blood pressure; FPG, fasting plasma glucose; 2hPG, 2 hour plasma glucose (after oral glucose tolerance test); TG, triglyceride; TC, total cholesterol; HDL-c, high-density lipoprotein cholesterol; LDL-c, low-density lipoprotein cholesterol; WHtR, waist-to-height ratio; BMI, body mass index; WHR, waist-to-hip ratio; NGT, normal glucose tolerance; IFG, impaired fasting glucose; IGT, impaired glucose tolerance; IFG+IGT, IFG combined with IGT; NDDM, newly-diagnosed diabetes mellitus.

Data are expressed as mean ± SD, or median (interquartile range), or N (%).

Mann-Whitney U analysis was used for DBP and BMI in Luzhou, TG and HDL-c in Wenjiang; one-way ANOVA analysis was used for the rest measurements in two surveys.

P value of men versus women.

For peer review only

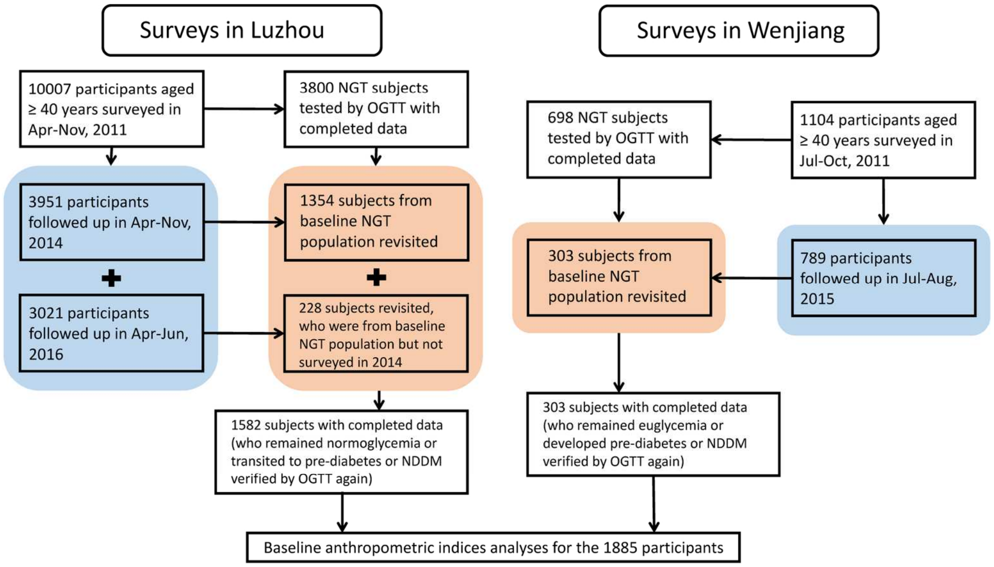

Supplemental Figure 1. Flow-chart of study design. NGT, normal glycemic tolerance; OGTT, oral glucose tolerance test; NDDM, newly-diagnosed diabetes mellitus. The re-visited participants in the blue background came from the baseline populations; the subjects in the pink background were the ones recruited in this study, who were normoglycemic at baseline.

165x94mm (300 x 300 DPI)

view only

STROBE 2007 (v4) checklist of items to be included in reports of observational studies in epidemiology*
Checklist for cohort, case-control, and cross-sectional studies (combined)

Section/Topic	Item #	Recommendation	Reported on page #
**Title and abstract**	1	(*a*) Indicate the study's design with a commonly used term in the title or the abstract	1, 2
		(*b*) Provide in the abstract an informative and balanced summary of what was done and what was found	2
**Introduction**			
Background/rationale	2	Explain the scientific background and rationale for the investigation being reported	4
Objectives	3	State specific objectives, including any pre-specified hypotheses	4
**Methods**			
Study design	4	Present key elements of study design early in the paper	4, 5
Setting	5	Describe the setting, locations, and relevant dates, including periods of recruitment, exposure, follow-up, and data collection	4. 5
Participants	6	(*a*) *Cohort study*—Give the eligibility criteria, and the sources and methods of selection of participants. Describe methods of follow-up<br>*Case-control study*—Give the eligibility criteria, and the sources and methods of case ascertainment and control selection. Give the rationale for the choice of cases and controls<br>*Cross-sectional study*—Give the eligibility criteria, and the sources and methods of selection of participants	4, 5
		(*b*) *Cohort study*—For matched studies, give matching criteria and number of exposed and unexposed<br>*Case-control study*—For matched studies, give matching criteria and the number of controls per case	NA
Variables	7	Clearly define all outcomes, exposures, predictors, potential confounders, and effect modifiers. Give diagnostic criteria, if applicable	5, 6
Data sources/ measurement	8*	For each variable of interest, give sources of data and details of methods of assessment (measurement). Describe comparability of assessment methods if there is more than one group	*5, 6*
Bias	9	Describe any efforts to address potential sources of bias	NA
Study size	10	Explain how the study size was arrived at	4, 5
Quantitative variables	11	Explain how quantitative variables were handled in the analyses. If applicable, describe which groupings were chosen and why	5, 6
Statistical methods	12	(*a*) Describe all statistical methods, including those used to control for confounding	6
		(*b*) Describe any methods used to examine subgroups and interactions	6
		(*c*) Explain how missing data were addressed	6
		(*d*) *Cohort study*—If applicable, explain how loss to follow-up was addressed<br>*Case-control study*—If applicable, explain how matching of cases and controls was addressed	6

For peer review only

		Cross-sectional study—If applicable, describe analytical methods taking account of sampling strategy	
		(*e*) Describe any sensitivity analyses	NA
**Results**			
Participants	13*	(a) Report numbers of individuals at each stage of study—eg numbers potentially eligible, examined for eligibility, confirmed eligible, included in the study, completing follow-up, and analysed	7
		(b) Give reasons for non-participation at each stage	5
		(c) Consider use of a flow diagram	5
Descriptive data	14*	(a) Give characteristics of study participants (eg demographic, clinical, social) and information on exposures and potential confounders	6
		(b) Indicate number of participants with missing data for each variable of interest	NA
		(c) *Cohort study*—Summarise follow-up time (eg, average and total amount)	5
Outcome data	15*	*Cohort study*—Report numbers of outcome events or summary measures over time	6
		Case-control study—Report numbers in each exposure category, or summary measures of exposure	NA
		Cross-sectional study—Report numbers of outcome events or summary measures	NA
Main results	16	(*a*) Give unadjusted estimates and, if applicable, confounder-adjusted estimates and their precision (eg, 95% confidence interval). Make clear which confounders were adjusted for and why they were included	7, 8
		(*b*) Report category boundaries when continuous variables were categorized	6-8
		(*c*) If relevant, consider translating estimates of relative risk into absolute risk for a meaningful time period	7, 8
Other analyses	17	Report other analyses done—eg analyses of subgroups and interactions, and sensitivity analyses	NA
**Discussion**			
Key results	18	Summarise key results with reference to study objectives	8
Limitations	19	Discuss limitations of the study, taking into account sources of potential bias or imprecision. Discuss both direction and magnitude of any potential bias	9, 10
Interpretation	20	Give a cautious overall interpretation of results considering objectives, limitations, multiplicity of analyses, results from similar studies, and other relevant evidence	8-10
Generalisability	21	Discuss the generalisability (external validity) of the study results	10
**Other information**			
Funding	22	Give the source of funding and the role of the funders for the present study and, if applicable, for the original study on which the present article is based	10

*Give information separately for cases and controls in case-control studies and, if applicable, for exposed and unexposed groups in cohort and cross-sectional studies.

Note: An Explanation and Elaboration article discusses each checklist item and gives methodological background and published examples of transparent reporting. The STROBE checklist is best used in conjunction with this article (freely available on the Web sites of PLoS Medicine at http://www.plosmedicine.org/, Annals of Internal Medicine at http://www.annals.org/, and Epidemiology at http://www.epidem.com/). Information on the STROBE Initiative is available at www.strobe-statement.org.

For peer review only

BMJ Open

Identical anthropometric characteristics of impaired fasting glucose combined with impaired glucose tolerance and newly-diagnosed type 2 diabetes: anthropometric indicators to predict hyperglycemia in a community-based prospective cohort study in southwest China

Journal:	*BMJ Open*
Manuscript ID	bmjopen-2017-019735.R2
Article Type:	Research
Date Submitted by the Author:	27-Feb-2018

Complete List of Authors:	Zhang, Fang; Sichuan University West China Hospital, Division of Endocrinology and Metabolism<br>Wan, Qin; Hospital affiliated to Southwest Medical University, Division of Endocrinology and Metabolism<br>Cao, Hongyi; The Fifth People's Hospital of Chengdu, Chengdu<br>Tang, Lizhi; Sichuan University West China Hospital, Division of Endocrinology and Metabolism<br>Li, Daigang; Chengdu Yinchao Community Hospital<br>Lü, Qingguo; Sichuan University West China Hospital<br>Yan, Zhe; Sichuan University West China Hospital<br>Li, Jing; Sichuan University West China Hospital<br>Yang, Qiu; The Fifth People's Hospital of Chengdu<br>Zhang, Yuwei; Sichuan University West China Hospital<br>Tong, Nanwei; Sichuan University West China Hospital, Division of Endocrinology and Metabolism

**Primary Subject Heading**:	Diabetes and endocrinology
Secondary Subject Heading:	Epidemiology, Diabetes and endocrinology
Keywords:	impaired fasting glucose combined with impaired glucose tolerance, newly-diagnosed diabetes mellitus, pre-diabetes, waist-to-height ratio, anthropometric measurements

SCHOLARONE™ Manuscripts

Title:

Identical anthropometric characteristics of impaired fasting glucose combined with impaired glucose tolerance and newly-diagnosed type 2 diabetes: anthropometric indicators to predict hyperglycemia in a community-based prospective cohort study in southwest China

Authors:

Fang Zhang 1, Qin Wan 2, Hongyi Cao 3, Lizhi Tang 1, Daigang Li 4, Qingguo Lü 1, Zhe Yan 1, Jing Li 1, Qiu Yang 3, Yuwei Zhang 1, Nanwei Tong 1△

△Corresponding author

Authors' affiliations:

1. Division of Endocrinology and Metabolism, West China Hospital of Sichuan University, Chengdu, Sichuan, China
2. Division of Endocrinology and Metabolism, Hospital affiliated to Southwest Medical University, Luzhou, Sichuan, China
3. Division of Endocrinology and Metabolism, The Fifth People's Hospital of Chengdu, Chengdu, Sichuan, China
4. Chengdu Yinchao Community Hospital, Chengdu, Sichuan, China

Correspondences:

Address correspondence and reprint requests to Nanwei Tong, MD, Division of Endocrinology and Metabolism, West China Hospital of Sichuan University, 37 Guoxuexiang, Chengdu, Sichuan 610041, China. Telephone number: 00+86-18980601196. Fax number: 00+86-28-85423459. E-mail: tongnw@scu.edu.cn.

For peer review only

Abstract

Objectives: To assess the anthropometric characteristics of normoglycemic individuals who subsequently developed hyperglycemia, and to evaluate the validity of these measures to predict pre-diabetes and diabetes.

Design: A community-based prospective cohort study.

Participants: In total, 1885 residents with euglycemia from six communities were enrolled.

Setting: Sichuan, southwest China

Primary outcome measures: The incidences of pre-diabetes and diabetes were the primary outcomes.

Methods: The waist-to-height ratio (WHtR), body mass index (BMI), waist circumference (WC), and waist-to-hip ratio (WHR) of all participants were measured at baseline and during follow-up. A 75 g glucose oral glucose tolerance test was conducted at each survey.

Results: During a median of 3.00 (interquartile range: 2.92—4.17) years follow-up, the cumulative incidence of isolated impaired fasting glucose (IFG), isolated impaired glucose tolerance (IGT), IFG combined with IGT (IFG+IGT), and newly-diagnosed diabetes mellitus (NDDM) were 8.44%, 18.14%, 8.06%, and 13.79%, respectively. WHtR, BMI, WC, and WHR were significantly different among subjects who subsequently progressed to isolated IFG or IGT, IFG+IGT, or NDDM ($P < 0.05$). The anthropometric characteristics of IFG+IGT subjects were similar to those of the NDDM population ($P > 0.005$). All the baseline anthropometric measurements were useful for the prediction of future pre-diabetes and NDDM ($P < 0.05$). The optimal thresholds for the four measurements were calculated for the prediction of hyperglycemia, with a WHtR value of 0.52 performing best to identify isolated IFG or IGT, IFG+IGT, and NDDM.

Conclusions: Anthropometric measures, especially WHtR, could be used to predict hyperglycemia 3 years in advance. Distinct from isolated IFG and IGT, the individuals who developed combined IFG+IGT had identical anthropometric profiles to those who progressed to NDDM.

Key words: anthropometric measurements, impaired fasting glucose combined with impaired glucose tolerance, newly-diagnosed diabetes mellitus, pre-diabetes, waist-to-height ratio

For peer review only

Strengths and limitations of this study

1. This study described and compared the anthropometric characteristics of participants who subsequently progressed to isolated impaired fasting glucose (IFG), isolated impaired glucose tolerance (IGT), IFG combined with IGT, newly-diagnosed diabetes mellitus (NDDM), or who remained normoglycemic.
2. Variations in waist-to-height ratio, body mass index, waist circumference, and waist-to-hip ratio, were used to predict the transition from euglycemia to pre-diabetes, and overt NDDM in the following 3 years.
3. The optimal threshold values for the prediction of hyperglycemia were determined from the anthropometric measurements collected.
4. The inherent limitations of the present work were a relatively short follow-up period (median 3 years), a low completion ratio of 41.9%, and a limited sample size, meaning that anthropometric threshold values could not be determined by gender for each category of hyperglycemia.

For peer review only

Introduction

The rapidly growing incidence of diabetes means that, it is now reaching epidemic proportions in China. The overall prevalences of diabetes and pre-diabetes were estimated to be 11.6% and 50.1%, respectively, in Chinese adults in 2010 [1]. In 2007—2008, another cross-sectional study conducted across China found that the prevalences of isolated impaired fasting glucose (IFG), isolated impaired glucose tolerance (IGT), and IFG combined with IGT (IFG+IGT), were 3.2%, 11.0%, and 1.9% in men, and 2.2%, 10.9%, and 1.7% in women, respectively [2]. Isolated IFG, isolated IGT, and IFG+IGT, were selected as three different categories of pre-diabetes, reflecting the progression from euglycemia to type 2 diabetes (T2D). Approximately 75%—80% of diabetes patients develop cardiovascular disease (CVD) ultimately, and patients with pre-diabetes have also been shown to be at greater risk of heart attack and stroke [3-5]. It has been estimated that between 2005 and 2015, diabetes and consequent CVD have cost China US$ 557.7 billion [6].

Measures to limit pre-diabetes are critical for the prevention of diabetes. Early recognition of pre-diabetes and prompt intervention could also reduce the impact on society as a whole. Both overall and central adiposity are closely linked to hyperglycemia. Body mass index (BMI) correlates with overall adiposity, while waist circumference (WC), waist-to-height ratio (WHtR), and waist-to-hip ratio (WHR) are indicators of central obesity. These four anthropometric indices are used globally to assess the risk of current or future diabetes [7-9].

Anthropometry is an affordable and practical screening tool for the presence of hyperglycemia, in both wealthy and impoverished areas of China. In this community-based prospective cohort study, we aimed to determine whether these anthropometric indices could predict future pre-diabetes and diabetes, and to establish optimal threshold values for the population. The baseline anthropometric characteristics of normoglycemic subjects, who subsequently developed isolated IFG, isolated IGT, IFG+IGT, and newly-diagnosed diabetes mellitus (NDDM) during follow-up, were compared and the similarities and differences between pairs of hyperglycemic categories were analyzed.

Study design and methods

Study population

The present study included two populations, in Luzhou City and in the Wenjiang area of Chengdu City. The Luzhou population are participants in the Risk Evaluation of cAncers in Chinese diabeTic Individuals: a lONgitudinal (REACTION) study, which is multicenter prospective observational study of 25 communities in mainland China [10, 11]. A total of 10007 residents, aged 40—89 years, were randomly recruited to participate in this study from five communities in Luzhou in 2011. Subjects with a history of diabetes, incident diabetes, or pre-diabetes, verified by an oral glucose tolerance test (OGTT), those missing values or any parameter, or having any of the other conditions (listed below), were excluded. After this, 3800 individuals with normoglycemia remained to form the baseline population. Of these, 1354 participants returned to complete the study in 2014. In addition, in 2016, 228 members of the baseline normoglycemic population who had not been studied in 2014, were followed up. Therefore, data from a total of 1582 subjects from Luzhou baseline screen were available

for analysis.

In the Wenjiang survey, a cohort of 1104 participants aged 40—75 years were randomly recruited from Yinchao community in 2011. Using the same inclusion criteria, 698 normoglycemic individuals comprised the baseline population. Of these, 303 subjects were followed up in 2015 and completed the study. Thus, from Luzhou and Wenjiang, a total of 1885 participants were included in the analysis.

All of the subjects were of Han Chinese ethnicity. A flow diagram of the study design is displayed as Supplemental Figure 1. Individuals with the following conditions were excluded from the study: infection, pregnancy, malignant tumor, acute cardiovascular accident, serious trauma, liver or renal dysfunction, or long history of glucocorticoid use. The research was conducted in accordance with the principles of the Declaration of Helsinki II. All protocols used in this work were approved either by the Medical Ethics Committee of the hospital affiliated to the Southwest Medical University in Luzhou, or by the Committee on Human Research at the Fifth People's Hospital of Chengdu in Wenjiang. Each participant provided written informed consent.

Patient and Public Involvement

All patients were randomly recruited to participate in this study and were interviewed face-to-face by trained investigators for detailed explanation of informed consent at the beginning. Three months later, each participant was received a health report with advised suggestions.

Diagnosis of diabetes and pre-diabetes

The diagnosis of hyperglycemic disorder was made in accordance with the American Diabetes Association recommendations, using OGTT, in 2011 [12]. Normal glycemic tolerance (NGT) was defined by a fasting plasma glucose (FPG) < 5.6 mmol/L and a 2-hour plasma glucose (2hPG) < 7.8 mmol/L. Isolated IFG was defined by 5.6 mmol/L ≤ FPG < 7.0 mmol/L and a 2hPG < 7.8 mmol/L, while isolated IGT was defined by an FPG < 5.6 mmol/L and 7.8 mmol/L ≤ 2hPG < 11.1 mmol/L. IFG+IGT was defined by 5.6 mmol/L ≤ FPG < 7.0 mmol/L and 7.8 mmol/L ≤ 2hPG < 11.1 mmol/L. Diabetes was defined by an FPG ≥ 7.0 mmol/L and/or a 2hPG ≥ 11.1 mmol/L.

Anthropometric measurements

Anthropometric measurements, including body mass, height, WC, and hip circumference were made by trained investigators. Measurements were conducted while all participants were wearing light clothing, without footwear after a 10—12 hour overnight fast in the morning. Measurements were made using calibrated weighing scales, standard steel strip stadiometers, and tape measures. The results were recorded to the nearest 0.1 kg or 0.1 cm. WC was measured at the midpoint between the costal border and the iliac crest at the end of exhalation. Hip circumference was measured around the widest portion of the buttocks. BMI was calculated as body mass (kg) divided by height squared (m^2), WHtR was calculated as WC (cm) divided by height (cm), and WHR as WC (cm) divided by hip circumference (cm).

For peer review only

Lifestyle variables and biological evaluation

Trained investigators collected lifestyle information, consisting of demographic characteristics, current smoking status, physical activity situation, medications, and personal and family disease histories, using a standard questionnaire and face-to-face interviews. The questionnaire categorized the participants into two groups: subjects undertaking vigorous physical activity ≥ 1 day per week and subjects undertaking vigorous physical activity on < 1 day per week. Blood pressure (BP) was measured three times in each participant using an electronic sphygmomanometer (OMRON, HEM-7220, Liaoning, China), with 5 min intervals between measurements, after at least 10 min rest, and the mean value was recorded.

All participants underwent an OGTT. After a 10—12 hour overnight fast, venous blood was drawn both before and 2 hours after they drank 300 ml water containing 75 g anhydrous glucose within 5 min. FPG and 2hPG concentrations were measured within 24 hours using the hexokinase method (Hitachi 7600 automatic biochemical analyzer, Hitachi Ltd., Tokyo, Japan). Fasting blood samples were collected for lipid profile measurements, including total cholesterol (TC), triglyceride (TG), high-density lipoprotein cholesterol (HDL-c), and low-density lipoprotein cholesterol (LDL-c). Serum TC, TG, and HDL-c concentrations were measured using oxidase colorimetric methods, and LDL-c concentration was measured by homogeneous assay, on a Hitachi 7600 automatic biochemical analyzer (Hitachi Ltd., Tokyo, Japan) within 24 hours. Hemoglobin A1c (HbA1c) was measured using the high performance liquid chromatography (VARIANT™ II TURBO Hemoglobin Testing System, Bio-Rad Laboratories, CA, USA). The samples were stored at − 20°C until analysis, which was undertaken within 3 weeks.

Statistical analysis

Data were analyzed using SPSS software version 16.0 (SPSS, Chicago, IL, USA) and MedCalc software version 15.2.2 (MedCalc software, Ostend, Belgium). All data are expressed as mean ± SD, median (interquartile range), or frequency (%), as appropriate. One-way ANOVA was used for parametric data, whereas the rank sum test was applied for non-parametric data. The chi-square test was used for the comparison of ratio. All tests were two-sided. In analyses of more than three groups, overall $P < 0.05$ was considered significant. The Bonferroni correction and chi-square segmentation were used for multiple comparison adjustments. For the comparison of two specific subgroups, $P < 0.005$ was considered significant. For BMI, WHtR, WC, and WHR, receiver operating characteristic (ROC) curve analyses were used to compare their ability to predict incident pre-diabetes and diabetes. The non-parametric approach described by DeLong *et al*. was used to compare the areas under ROC curves [13]. The predictive threshold values for hyperglycemia were calculated. COX proportional hazards regression was used to evaluate associations between anthropometric indices and hyperglycemic categories; the time axis consisted of the period of follow-up until pre-diabetes or diabetes developed, or the end of the study. Hazard ratio (HR) and 95% confidence interval (CI) were calculated.

Results

For peer review only - http://bmjopen.bmj.com/site/about/guidelines.xhtml

Characteristics of subjects at baseline

A total of 1885 normoglycemic subjects (649 men and 1236 women), with a median age of 56 (interquartile range: 48—61) years old, were recruited in 2011. After a median follow-up of 3.00 (2.92—4.17) years, 159 individuals had developed isolated IFG, 342 had developed isolated IGT, 152 had developed IFG+IGT, 260 had developed NDDM, and the remaining 972 participants remained normoglycemic. The incidences of pre-diabetes and NDDM were calculated to be 104.9 per 1000 person-years and 41.8 per 1000 person-years, respectively. The characteristics of all the subjects at baseline in Luzhou and Wenjiang are shown in Supplemental Table 1. The participants in Luzhou were older than the participants in Wenjiang, and had higher glucose levels at baseline and greater incidences of pre-diabetes and diabetes during follow-up. The baseline measurements of the participants who subsequently developed isolated IFG, isolated IGT, IFG+IGT, or NDDM in the future, are shown in Table 1. The subjects who developed NDDM were the oldest group at baseline of the five groups (P = 0.000). The individuals who transited to isolated IGT, IFG+IGT, or NDDM had higher baseline HbA1c levels than the subjects who remained normoglycemic ($P < 0.005$).

Baseline and follow-up anthropometric values in subjects who subsequently developed hyperglycemic disorders

During the follow-up examination, it was found that WHtR in the NGT group was lower than in the isolated IGT, IFG+IGT, or NDDM groups ($P < 0.005$) (Table 2), and was lower in the isolated IFG and isolated IGT groups than in the IFG+IGT and NDDM groups ($P < 0.005$). The P values were 0.009 and 0.006 for BMI in isolated IFG versus IFG+IGT, and isolated IGT versus IFG+IGT, respectively, and 0.005 for WHR in the isolated IFG or IGT groups versus the IFG+IGT group. There were the trends towards the differences in both BMI and WHR between the isolated IFG or IGT groups, and the IFG+IGT group. To summarize, BMI, WC, and WHR in the five hyperglycemic groups tended to follow the following pattern: NGT < isolated IFG and isolated IGT < IFG+IGT and NDDM. Unlike when the isolated IFG or isolated IGT groups were compared, the anthropometric characteristics of the IFG+IGT group were similar to those of the NDDM at follow-up ($P > 0.005$).

To assess whether the anthropometric values were already different before hyperglycemia developed, we evaluated the differences between groups at baseline, when all the subjects were still normoglycemic. Baseline WHtR, BMI, and WHR, but not WC, substantially differed among the five groups ($P < 0.05$) (Table 2). NGT subjects had lower WHtR than the subjects who subsequently developed hyperglycemia ($P < 0.005$). The WHtR values of the IFG+IGT and NDDM groups were higher than those of the isolated IFG group ($P < 0.005$), while the isolated IGT group had a lower WHtR than the NDDM group ($P < 0.005$). The BMI of the NGT group was lower than those of the isolated IGT, IFG+IGT, and NDDM groups ($P < 0.005$), and the isolated IFG group had a lower BMI than NDDM subjects ($P < 0.005$). In addition, NGT individuals had a lower WHR than NDDM patients at baseline ($P < 0.005$). Consistent with the findings at follow-up, it is worth noting that at baseline, there were no significant differences in WHtR, BMI, and WHR between individuals who subsequently developed IFG+IGT and those who converted to NDDM ($P > 0.005$).

For peer review only

Use of baseline anthropometric indices to predict future pre-diabetes and NDDM

For the prediction of isolated IFG, baseline WHtR, WC, and WHR showed significantly different areas under the curve (AUCs) (P < 0.05) (Table 3). WHtR and WC were more effective at predicting isolated IFG than BMI (P < 0.05) (Figure 1A). For subjects who developed isolated IGT, the AUCs of all the four indices were significant (P = 0.000). WHtR had a higher predictive value than BMI, WC, and WHR (P < 0.05), while WC was superior to WHR for predicting isolated IGT (P < 0.05) (Figure 1B). For IFG+IGT incidence, all four parameters were valuable predictors (P = 0.000), among which WHtR and WC ranked higher than WHR (P < 0.05) (Figure 1C). For the prediction of NDDM, the four indices were significant (P < 0.05), but WHtR was the best predictor (P < 0.05) (Figure 1D). The optimal thresholds for predicting hyperglycemia for the four indices (WC and WHR thresholds for men and women) were then calculated.

Multivariate analysis of baseline anthropometric indices with respect to risk of subsequent pre-diabetes and NDDM

According to COX proportional hazards regression, the risk of developing isolated IFG was greater with higher WC at baseline (P < 0.05) (Table 4). The risk factors for the development of isolated IGT were baseline WHtR, BMI, and WC (P < 0.05). For both IFG+IGT and NDDM, high baseline WHtR, BMI, WC, and WHR were all risk factors (P < 0.05).

Discussion

In this community-based prospective cohort study, we have shown that: (1) For patients with hyperglycemia, WHtR, BMI, WC, and WHR tended to be as follows: NGT < isolated IFG and isolated IGT < IFG+IGT and NDDM. (2) Among these categories of hyperglycemia, it is noteworthy that unlike with respect to isolated IFG and isolated IGT, there were no significant differences in baseline WHtR or BMI between subjects with IFG+IGT and NDDM. (3) Thus, WHtR, BMI, WC, and WHR could predict the presence of pre-diabetes or diabetes 3 years in advance. Furthermore, the greater were these baseline anthropometric values, the higher was the risk of developing hyperglycemia. (4) Optimal threshold values for the four variables for identification of pre-diabetes and diabetes were calculated, with WHtR performing best of these in the prediction of hyperglycemia.

An Iranian study of 5879 people 9 years after they were initially found to be normoglycemic, found that 1755 subjects had developed pre-diabetes, and that isolated IFG was the commonest pre-diabetic phenotype. This study found that among women, in contrast to the use of BMI, hip and waist circumferences, WHtR was the only significant anthropometric predictor of pre-diabetes [14]. Lyssenko *et al.* reported a study of 1190 subjects in Finland who initially had NGT. During a median follow-up of 6 years, 199 had progressed to pre-diabetes. Compared with those who remained NGT, those with pre-diabetes had substantially higher BMI and WHtR at baseline [15]. Many investigators have shown that anthropometry is tightly correlated with the occurrence of pre-diabetes, although most of the studies conducted have been cross-sectional, rather than longitudinal [16-19].

After reviewing the literature, we found some common themes: (1) With respect to

For peer review only

pre-diabetes, the majority of the studies only defined one or two distinct pre-diabetic phenotypes, or defined a single category called "pre-diabetes". (2) Rarely did investigators describe the respective anthropometric characteristics of the various hyperglycemic disorders in their manuscripts. We located only one previous report that gave anthropometric information in detail for all the potential pre-diabetic phenotypes and NDDM [20]. It was shown in this study that WHtR, BMI, WC, and WHR varied substantially among subjects with NGT, isolated IFG, isolated IGT, IFG+IGT, and NDDM, but none of the anthropometric indices were compared between hyperglycemic groups. Therefore, the possibility that anthropometry might vary between pre-diabetes and NDDM could not be assessed, and moreover, this study was cross-sectional. To our knowledge, the present work is the first prospective cohort study that not only described the anthropometric characteristics of participants who progressed to diverse hyperglycemic conditions, but also demonstrated the variation among WHtR, BMI, WC, and WHR in the transition from NGT to pre-diabetes and overt NDDM.

The pathogenesis of isolated IFG and isolated IGT is heterogeneous, while individuals with IFG+IGT manifest both hepatic and peripheral insulin resistance. Pre-diabetes, as an intermediate hyperglycemic state, carries a high-risk for the subsequent development of diabetes. Among the three pre-diabetic phenotypes, IFG+IGT carries approximately twice the risk of transition to diabetes compared with subjects with just one of abnormalities [21]. In our previous work, we found that several biomarkers in individuals with IFG+IGT had similar values to those present in the NDDM population, but these were different in individuals with IFG or IGT alone [22-24]. Consistent with this, in the present study we observed that participants who subsequently developed hyperglycemia had higher WHtR, BMI, and WHR at baseline than those who remained NGT. Among the three pre-diabetic phenotypes, IFG+IGT subjects had the most adverse anthropometric profiles at baseline, such that there were no significant differences from the NDDM group. These findings may imply that although IFG+IGT is a subtype of pre-diabetes, some aspects of its pathophysiology have already deteriorated to the same extent as in NDDM. However, pre-diabetes is a reversible condition and consequently, prompt intervention is required to avoid or delay its progression, especially for patients with IFG+IGT.

A prospective study conducted in Pima Indians found that BMI and WHtR were the best predictors of diabetes in men, while BMI, WHtR, WC, and waist-to-thigh ratio were the best predictors in women [25]. Chei *et al.* published a cohort study of 5617 Japanese participants, finding that in women only, the significant predictors of T2D were BMI, WC, and WHtR [26]. Finally, in a multi-ethnic cohort of 1073 non-Hispanic white, Hispanic, and African American non-diabetic individuals, baseline anthropometric information showed that BMI was most predictive of diabetes in the non-Hispanic white and Hispanic populations, whereas all the indicators of central obesity were more predictive than measures of overall adiposity in the African American population [27]. The contrasts in these sets of data indicate that the validity of such anthropometric measurements for the prediction of diabetes development vary among different ethnicities, genders, and regions. Based on our ROC analysis, WHtR was most effective for the prediction of pre-diabetes and overt NDDM, followed by WC, while BMI

For peer review only

and WHR were relatively weak predictors. Results from two western Pacific studies were consistent with our findings [28, 29].

A systematic review proposed that the threshold values for WHtR in the prediction of diabetes in men and women are 0.52 and 0.53, respectively [30]. In a Chinese community-based prospective cohort study, the optimal threshold values for WHtR and BMI were 0.51 and 24 for men, and 0.55 and 25 for women, respectively [29]. These predictive values were similar to those identified in our study.

Several limitations to our work should be addressed. First, the follow-up period of a median 3.00 years was relatively short. However, we identified high cumulative incidences of pre-diabetes and NDDM (34.6% and 13.8%, respectively). The fast pace of life and sedentary lifestyle of the population may be the main contributor to the rapid growth in hyperglycemia. However, it might also be the result of selection bias, because subjects with a higher risk might be more likely to take part in the follow-up assessment. In addition, the participants were ≥ 40 years old, a little older than the subjects (≥ 35 years) in some other epidemiological studies. This might be also an explanation that a large proportion of subjects became hyperglycemic in this cohort study. Second, the proportion of participants attending the follow-up assessment was low (41.91%). Conducting of a phone interview once a year at least, followed by prompt examination, could improve this statistic in the future. Third, the sample size was limited. On account of this weakness, it was not possible to calculate anthropometric threshold values for each hyperglycemic state by gender. Further studies are required to establish specific screening thresholds for pre-diabetes and NDDM in men and women, especially with regard to WC and WHR. Fourth, there was lack of OGTT reproducibility in each set of measurements. Unwillingness of subjects, and limited staff and financial resources, were the two major causes of this. By combining these data with the questionnaire data and the HbA1c results, we tried to minimize the associated error and improve the diagnostic accuracy as much as possible.

In summary, WHtR, BMI, WC, and WHR are all predictors of the development of pre-diabetes and NDDM 3 years in advance. Individuals with high WHtR, BMI, WC, and WHR are thus at higher risk of developing pre-diabetes and T2D. The optimal thresholds for all the anthropometric measures to predict hyperglycemia were calculated, with a WHtR value of 0.52 performing best at predicting the development of isolated IFG or IGT, IFG+IGT, and NDDM. The magnitude of WHtR and BMI in normoglycemic subjects illustrate the likelihood of progression from normoglycemia to pre-diabetes, and then to overt T2D. Of note, and in contrast to the situation with regard to isolated IFG or IGT, the anthropometric characteristics of IFG+IGT subjects were similar to those of the NDDM population, both at baseline and follow-up.

Contributorship statement

For peer review only

All the authors engaged in the surveys. FZ and NT designed this article. QW, HC, DL and QY acquired and collected data. JL, ZY, QL and YZ organized all the data. FZ, QW and HC analyzed all the information. FZ and LT drafted the manuscript. FZ and NT revised the article critically. All the authors read and approved the final manuscript.

Acknowledgements

All the authors appreciated the doctors and other staffs from primary health care centers for health report suggestions, materials supplement, and participant organization.

Competing interests

All the authors declared that there were no competing interests among them.

Fundings

The surveys in Luzhou were supported by the grants from the National Clinical Research Center for Metabolic Diseases (2013BAI09B13), the National Key New Drug Creation and Manufacturing Program of Ministry of Science and Technology (2012ZX09303006—001). The screenings in Wenjiang were supported by the National Key Technology R&D Program of China (Grant No. 2009BAI80B01 and No. 2009BAI80B02) and Sichuan Provincial Science and Technology Foundation (Grant No. 150022 and No. 2015SZ0228).

Data sharing statement

A supplementary profile will be available online which contains comprehensive figure and table of used input data. Inquiries about additional unpublished data could be contacted with the corresponding author.

References

1. Xu Y, Wang L, He J, Bi Y, Li M, Wang T, Wang L, Jiang Y, Dai M, Lu J, Xu M, Li Y, Hu N, Li J, Mi S, Chen CS, Li G, Mu Y, Zhao J, Kong L, Chen J, Lai S, Wang W, Zhao W, Ning G; 2010 China Noncommunicable Disease Surveillance Group. Prevalence and Control of Diabetes in Chinese Adults. *JAMA.* 2013; 310 (9): 948-958.
2. Yang W, Lu J, Weng J, Jia W, Ji L, Xiao J, Shan Z, Liu J, Tian H, Ji Q, Zhu D, Ge J, Lin L, Chen L, Guo X, Zhao Z, Li Q, Zhou Z, Shan G, He J; China National Diabetes and Metabolic Disorders Study Group. Prevalence of Diabetes among Men and Women in China. *N Engl J Med.* 2010; 362: 1090-101.
3. Rydén L, Grant PJ, Anker SD, Berne C, Cosentino F, Danchin N, Deaton C, Escaned J, Hammes HP, Huikuri H, Marre M,Marx N, Mellbin L, Ostergren J, Patrono C, Seferovic P, Uva MS, Taskinen MR, Tendera M, Tuomilehto J, Valensi P, Zamorano

JL; ESC Committee for Practice Guidelines (CPG), Zamorano JL, Achenbach S, Baumgartner H, Bax JJ, Bueno H, Dean V, Deaton C, Erol C, Fagard R, Ferrari R, Hasdai D, Hoes AW, Kirchhof P, Knuuti J, Kolh P, Lancellotti P, Linhart A, Nihoyannopoulos P, Piepoli MF, Ponikowski P, Sirnes PA, Tamargo JL, Tendera M, Torbicki A,Wijns W, Windecker S; Document Reviewers, De Backer G, Sirnes PA, Ezquerra EA, Avogaro A, Badimon L, Baranova E, Baumgartner H, Betteridge J,Ceriello A, Fagard R, Funck-Brentano C, Gulba DC, Hasdai D, Hoes AW, Kjekshus JK, Knuuti J, Kolh P, Lev E, Mueller C, Neyses L, Nilsson PM, Perk J,Ponikowski P, Reiner Z, Sattar N, Schächinger V, Scheen A, Schirmer H, Strömberg A, Sudzhaeva S, Tamargo JL, Viigimaa M, Vlachopoulos C, Xuereb RG. ESC Guidelines on diabetes, pre-diabetes, and cardiovascular diseases developed in collaboration with the EASD. *European Heart Journal.* 2013; 34: 3035–3087.

4. Tanne D, Koren-Morag N, Goldbourt U. Fasting plasma glucose and risk of incident ischemic stroke or transient ischemic attacks: a prospective cohort study. *Stroke.* 2004; 35:2351-5.
5. Norhammar A, Tenerz A, Nilsson G, Hamsten A, Efendíc S, Rydén L, Malmberg K. Glucose metabolism in patients with acute myocardial infarction and no previous diagnosis of diabetes mellitus: a prospective study. *Lancet.* 2002; 359: 2140-4.
6. Chinese Diabetes Society. China Guideline for Type 2 Diabetes. *Chinese Diabetes Society.* 2010. (Chinese)
7. Chen L, Magliano DJ, Balkau B, Colagiuri S, Zimmet PZ, Tonkin AM, Mitchell P, Phillips PJ, Shaw JE. AUSDRISK: an Australian type 2 diabetes risk assessment tool based on demographic, lifestyle and simple anthropometric measures. *Medical Journal of Australia.* 2010; 192 (4): 197–202.
8. Lindstrom J, Tuomilehto J. The diabetes risk score: a practical tool to predict type 2 diabetes risk. *Diabetes Care.* 2003; 26: (3)725–731.
9. Mohan V, Sandeep S, Deepa M, Gokulakrishnan K, Datta M, Deepa R. A diabetes risk score helps identify metabolic syndrome and cardiovascular risk in Indians – the Chennai Urban Rural Epidemiology Study (CURES-38). *Diabetes, Obesity and Metabolism.* 2007; 9 (3): 337–343.
10. Lu J, Bi Y, Wang T, Wang W, Mu Y, Zhao J, Liu C, Chen L, Shi L, Li Q, Wan Q, Wu S, Qin G, Yang T, Yan L, Liu Y, Wang G, Luo Z, Tang X, Chen G, Huo Y, Gao Z, Su Q, Ye Z, Wang Y, Deng H, Yu X, Shen F, Chen L, Zhao L, Dai M, Xu M, Xu Y, Chen Y, Lai S, Ning G. The relationship between insulin-sensitive obesity and cardiovascular diseases in a Chinese population: Results of the REACTION study. *International Journal of Cardiology.* 2014; 172: 388–394.
11. Bi Y, Lu J, Wang W, Mu Y, Zhao J, Liu C, Chen L, Shi L, Li Q, Wan Q, Wu S, Yang T, Yan L, Liu Y, Wang G, Luo Z, Tang X, Chen G, Huo Y, Gao Z, Su Q, Ye Z,Wang Y, Qin G, Deng H, Yu X, Shen F, Chen L, Zhao L, Zhang J, Sun J, Dai M, Xu M, Xu Y, Chen Y, Lai S, Bloomgarden ZT, Li D, Ning G. Cohort profile: Risk evaluation of cancers in Chinese diabetic individuals: A longitudinal (REACTION) study. *J Diabetes.* 2014; 6 (2): 147–57.
12. American Diabetes Association. Standards of medical care in diabetes–2010. *Diabetes Care.* 2011; 33 (Suppl. 1): S11–61.

For peer review only

13. DeLong ER, DeLong DM, Clarke-Pearson DL. Comparing the areas under two or more correlated receiver operating characteristic curves: a nonparametric approach. *Biometrics.* 1988; 44 (3): 837-45.

14. Hadaegh F, Derakhshan A, Zafari N, Khalili D, Mirbolouk M, Saadat N, Azizi F. Pre-diabetes tsunami: incidence rates and risk factors of pre-diabetes and its different phenotypes over 9 years of follow-up. *Diabet Med*. 2017; 34 (1): 69-78.

15. Lyssenko V, Almgren P, Anevski D, Perfekt R, Lahti K, Nissén M, Isomaa B, Forsen B, Homström N, Saloranta C, Taskinen MR, Groop L, Tuomi T, Botnia study group. Predictors of and longitudinal changes in insulin sensitivity and secretion preceding onset of type 2 diabetes. *Diabetes.* 2005; 54 (1):166-74.

16. Xu Z, Qi X, Dahl AK, Xu W. Waist-to-height ratio is the best indicator for undiagnosed type 2 diabetes. *Diabet Med*. 2013; 30 (6):e201-7.

17. Hu D, Xie J, Fu P, Zhou J, Yu D, Whelton PK, He J, Gu D. Central rather than overall obesity is related to diabetes in the Chinese population: the InterASIA study. *Obesity (Silver Spring)*. 2007 Nov; 15 (11):2809-16.

18. Jung SH, Ha KH, Kim DJ. Visceral fat mass has stronger associations with diabetes and prediabetes than other anthropometric obesity indicators among Korean adults. *Yonsei Med J.* 2016 May; 57 (3):674-80.

19. Hayashi T, Boyko EJ, Leonetti DL, McNeely MJ, Newell-Morris L, Kahn SE, Fujimoto WY. Visceral adiposity and the risk of impaired glucose tolerance: a prospective study among Japanese Americans. *Diabetes Care*. 2003; 26 (3):650-5.

20. Alperet DJ, Lim WY, Mok-Kwee Heng D, Ma S, van Dam RM. Optimal anthropometric measures and thresholds to identify undiagnosed type 2 diabetes in three major Asian ethnic groups. *Obesity (Silver Spring).* 2016 Oct; 24 (10):2185-93.

21. Nathan DM, Davidson MB, DeFronzo RA, Heine RJ, Henry RR, Pratley R, Zinman B, American Diabetes Association. Impaired fasting glucose and impaired glucose tolerance: implications for care. *Diabetes Care*. 2007 Mar; 30 (3):753-9.

22. Huang L, Tong Y, Zhang F, Yang Q, Li D, Xie S, Li Y, Cao H, Tang L, Zhang X, Tong N. Increased acyl ghrelin but decreased total ghrelin and unacyl ghrelin in Chinese Han people with impaired fasting glucose combined with impaired glucose tolerance. *Peptides*. 2014 Oct; 60:86-94.

23. Lü Q, Tong N, Liu Y, Li N, Tang X, Zhao J, Cao H, Li D, Gou L, Zhang Y, Wan J, Jiang L. Community-based population data indicates the significant alterations of insulinresistance , chronic inflammation and urine ACR in IFG combined IGT group amongprediabetic pop ulation. *Diabetes Res Clin Pract*. 2009 Jun; 84 (3):319-24.

24. Zhang F, Tang X, Cao H, Lü Q, Li N, Liu Y, Zhang X, Zhang Y, Cao M, Wan J, An Z, Tong N. Impaired secretion of total glucagon-like peptide-1 in people with impaired fasting glucose combined impaired glucose tolerance. *Int J Med Sci.* 2012; 9 (7):574-81.

25. Tulloch-Reid MK, Williams DE, Looker HC, Hanson RL, Knowler WC. Do measures of body fat distribution provide information on the risk of type 2 diabetes in addition to measures of general obesity? Comparison of anthropometric predictors of

type 2 diabetes in Pima Indians. *Diabetes Care*. 2003 Sep; 26 (9):2556-61.

26. Chei CL, Iso H, Yamagishi K, Tanigawa T, Cui R, Imano H, Kiyama M, Kitamura A, Sato S, Shimamoto T. Body fat distribution and the risk of hypertension and diabetes among Japanese men and women. *Hypertens Res.* 2008 May; 31 (5):851-7.
27. MacKay MF, Haffner SM, Wagenknecht LE, D'Agostino RB Jr, Hanley AJ. Prediction of type 2 diabetes using alternate anthropometric measures in a multi-ethnic cohort: the insulin resistance atherosclerosis study. *Diabetes Care.* 2009 May; 32 (5):956-8.
28. Son YJ, Kim J, Park HJ, Park SE, Park CY, Lee WY, Oh KW, Park SW, Rhee EJ. Association of Waist-Height Ratio with Diabetes Risk: A 4-Year Longitudinal Retrospective Study. *Endocrinol Metab (Seoul).* 2016 Mar; 31 (1):127-33.
29. Xu F, Wang YF, Lu L, Liang Y, Wang Z, Hong X, Li J. Comparison of anthropometric indices of obesity in predicting subsequent risk of hyperglycemia among Chinese men and women in Mainland China. *Asia Pac J Clin Nutr.* 2010; 19 (4):586-93.
30. Browning LM, Hsieh SD, Ashwell M. A systematic review of waist-to-height ratio as a screening tool for the prediction of cardiovascular disease and diabetes: 0·5 could be a suitable global boundary value. *Nutrition Research Reviews.* 2010; 23: 247–269.

For peer review only

Table 1. General measurements of subjects at baseline who progressed to hyperglycemia at follow-up

	NGT (n = 972)	Isolated IFG (n = 159)	Isolated IGT (n = 342)	IFG+IGT (n = 152)	NDDM (n = 260)	overall *P* value
Follow-up time (year)	3.00 (2.92—4.17)‡	3.00 (2.92—4.17)‡	2.92 (2.92—3.17)*†	3.00 (2.92—3.17)	3.00 (2.92—3.17)	0.000
Age (year)	53 (46—59)†‡§¶	55 (48—62)*¶	59 (49—65)*	56 (49—62)*¶	60 (54—65)*†§	0.000
Female (N/n%)	675 (69.44%)	96 (60.38%)	220 (64.33%)	97 (63.82%)	166 (63.85%)	0.075
Height (cm)	158.00 (153.10—164.00)	159.45 (154.00—165.52)	157.00 (152.00—162.70)	157.10 (154.00—164.00)	156.00 (152.00—163.20)	0.492
Weight (kg)	58.00 (52.00—65.00)	60.50 (53.99—66.85)	60.00 (53.00—66.20)	62.10 (56.70—69.50)	62.00 (55.00—69.75)	0.498
Hip circumference (cm)	93.00 (88.20—97.20)	94.00 (90.00—99.00)	95.00 (90.20—100.00)	96.00 (92.00—100.30)	96.00 (92.00—101.00)	0.879
SBP (mmHg)	115.67 (105.33—128.67)‡§¶	118.50 (107.46—133.00)¶	122.50 (109.33—136.67)*¶	123.00 (114.00—137.67)*	130.67 (118.67—142.17)*†‡	0.000
DBP (mmHg)	74.33 (68.00—81.33)§¶	77.00 (70.00—83.75)	76.33 (69.00—83.33)¶	77.50 (72.33—82.67)*	79.00 (72.33—88.17)*‡	0.000
FPG (mmol/L)	5.08 (4.83—5.29)†§¶	5.20 (4.98—5.38)*	5.11 (4.90—5.33)	5.16 (4.93—5.36)*	5.16 (4.92—5.36)*	0.000
2hPG (mmol/L)	6.15 (5.40—6.88)‡§	6.14 (5.45—6.93)	6.40 (5.67—7.09)*	6.54 (5.85—7.10)*	6.33 (5.50—7.08)	0.000
HbA1c (%)	5.60 (5.30—5.90)‡§¶	5.70 (5.48—5.90)	5.70 (5.40—5.90)*	5.70 (5.50—6.00)*	5.70 (5.40—6.00)*	0.000
TG (mmol/L)	1.10 (0.80—1.60)	1.12 (0.80—1.63)	1.11 (0.84—1.59)	1.14 (0.89—1.60)	1.07 (0.81—1.50)	0.494
TC (mmol/L)	4.46 ± 1.01	4.45 ± 1.17	4.50 ± 1.02	4.72 ± 1.14	4.52 ± 1.10	0.062
HDL-c (mmol/L)	1.32 (1.09—1.60)	1.32 (1.05—1.52)	1.30 (1.08—1.56)	1.36 (1.20—1.57)	1.31 (1.09—1.60)	0.376
LDL-c (mmol/L)	2.51 (2.04—3.03)	2.44 (1.97—3.09)	2.53 (1.99—2.99)	2.65 (2.06—3.17)	2.45 (1.95—3.01)	0.688
Family history of diabetes (N/%)	119 (12.24%)	12 (7.55%)	29 (8.48%)	18 (11.84%)	29 (11.15%)	0.214
Current smoker (N/%)	137 (14.10%)	28 (17.61%)	42 (12.28%)	18 (11.84%)	44 (16.92%)	0.307
Physical activity (N/%)	719 (73.97%)	107 (67.30%)	255 (74.56%)	113 (74.34%)	195 (75.00%)	0.435

For peer review only

NGT, normal glucose tolerance; IFG, impaired fasting glucose; IGT, impaired glucose tolerance; IFG+IGT, IFG combined with IGT; NDDM, newly-diagnosed diabetes mellitus; SBP, systolic blood pressure; DBP, diastolic blood pressure; FPG, fasting plasma glucose; 2hPG, 2 hour plasma glucose (after oral glucose tolerance test); TG, triglyceride; TC, total cholesterol; HDL-c, high-density lipoprotein cholesterol; LDL-c, low-density lipoprotein cholesterol.

Data are expressed as means ± SD or median (interquartile range) or N (%).

Chi-square test was used to compare gender compositions, family history of diabetes, current smoking status and physical activity among five groups. If needed, chi-square segmentation was applied for further comparisons between any two subgroups with an adjusted significance level (a' = 0.005).

Kruskal-Wallis H analysis was applied for follow-up time among five groups. Mann-Whitney U analysis was performed for comparison within any two subgroups additionally (a' = 0.005).

One-way ANOVA analysis was used for the rest measurements among five groups, while LSD analysis was applied for age, SBP, DBP, FPG, 2hPG and HbA1c comparisons between any two subgroups (a' = 0.005).

*, versus NGT and $P < 0.005$; †, versus isolated IFG and $P < 0.005$; ‡, versus isolated IGT and $P < 0.005$; §, versus IFG+IGT and $P < 0.005$; ¶, versus NDDM and $P < 0.005$.

For peer review only

For peer review only

Table 2. Baseline and follow-up anthropometric values in participants who developed hyperglycemic disorders

	NGT (n = 972)	Isolated IFG (n = 159)	Isolated IGT (n = 342)	IFG+IGT (n = 152)	NDDM (n = 260)	overall *P* value
At follow-up survey						
WHtR (cm/cm)	0.51 (0.47—0.55)‡§¶	0.52 (0.48—0.56)§¶	0.53 (0.49—0.57)*§¶	0.54 (0.51—0.59)*†‡	0.56 (0.52—0.60)*†‡	0.000
BMI (kg/m^2)	23.46 (21.77—25.53)†‡§¶	24.27 (22.49—26.17)*¶	24.44 (22.63—26.50)*¶	25.09 (23.62—27.01)*	25.73 (23.29—27.82)*†‡	0.000
Waist circumference (cm)	80.65 (74.00—87.00)†‡§¶	82.80 (77.00—91.00)*§¶	84.00 (78.00—90.00)*§¶	86.70 (80.28—93.00)*†‡	88.00 (82.00—95.00)*†‡	0.000
WHR (cm/cm)	0.86 (0.81—0.91)†‡§¶	0.88 (0.84—0.92)*¶	0.88 (0.83—0.92)*¶	0.90 (0.86—0.94)*	0.91 (0.87—0.95)*†‡	0.000
At baseline survey						
WHtR (cm/cm)	0.50 ± 0.05†‡§¶	0.52 ± 0.06*§¶	0.53 ± 0.05*¶	0.54 ± 0.05*†	0.55 ± 0.06*†‡	0.000
BMI (kg/m^2)	23.03 (21.23—25.16)‡§¶	23.31 (21.56—25.64)¶	24.03 (22.10—26.22)*	24.98 (23.47—26.67)*	25.42 (23.17—27.22)*†	0.000
Waist circumference (cm)	79.00 (73.00—86.00)	82.00 (76.00—89.00)	83.00 (77.10—89.00)	87.00 (81.00—91.28)	86.00 (80.00—93.00)	0.282
WHR (cm/cm)	0.86 (0.81—0.90)¶	0.87 (0.92—0.92)	0.87 (0.82—0.91)	0.89 (0.86—0.93)	0.90 (0.86—0.94)*	0.010

NGT, normal glucose tolerance; IFG, impaired fasting glucose; IGT, impaired glucose tolerance; IFG+IGT, IFG combined with IGT; NDDM, newly-diagnosed diabetes mellitus; WHtR, waist-to-height ratio; BMI, body mass index; WHR, waist-to-hip ratio.

Data are expressed as median (interquartile range) or means ± SD.

At follow-up survey: One-way ANOVA analysis was used for WHtR, BMI and WC among the five glucose metabolic groups. LSD analysis was applied for the further comparisons between any two subgroups (a' = 0.005). Kruskal-Wallis H analysis was applied for WHR among the five groups and Mann-Whitney U analysis was performed for the following comparisons within any two subgroups (a' = 0.005).

At baseline survey: One-way ANOVA analysis was used for all indices among the five glucose metabolic groups. LSD analysis was applied for WHtR, BMI and WHR between any two subgroups' comparison (a' = 0.005).

*, versus NGT and $P < 0.005$; †, versus isolated IFG and $P < 0.005$; ‡, versus isolated IGT and $P < 0.005$; §, versus IFG+IGT and $P < 0.005$; ¶, versus NDDM and *P*

< 0.005.

For peer review only

Table 3. ROC curve analysis of baseline anthropometric indices for predicting future hyperglycemia

	AUC	SE	*P* value	95%CI	Cut-off point	Youden's value	Sensitivity	Specificity	DeLong's test (*P* value) WHtR (cm/cm)	BMI (kg/m^2)	Waist circumference (cm)	WHR (cm/cm)
Isolated IFG												
WHtR (cm/cm)	0.578	0.025	0.002	(0.529—0.626)	0.51	0.151	54.90%	60.19%	—	0.010	0.201	0.611
BMI (kg/m^2)	0.544	0.025	0.081	(0.495—0.593)	21.36	0.078	80.40%	27.43%	0.010	—	0.023	0.421
Waist circumference (cm)	0.592	0.024	0.000	(0.545—0.639)	77.10	0.148	71.24%	43.54%	0.201	0.023	—	0.195
Women	0.584	0.031	0.010	(0.524—0.644)	75.00	0.166	74.44%	42.11%	—	—	—	—
Men	0.579	0.041	0.050	(0.526—0.631)	87.00	0.165	49.21%	67.24%	—	—	—	—
WHR (cm/cm)	0.567	0.026	0.008	(0.537—0.597)	0.88	0.128	47.06%	65.71%	0.611	0.421	0.195	—
Women	0.568	0.033	0.036	(0.504—0.632)	0.85	0.140	57.78%	56.19%	—	—	—	—
Men	0.525	0.042	0.534	(0.471—0.578)	0.90	0.095	53.97%	55.52%	—	—	—	—
Isolated IGT												
WHtR (cm/cm)	0.634	0.017	0.000	(0.600—0.667)	0.51	0.214	62.24%	59.12%	—	0.003	0.006	0.000

For peer review only

BMI (kg/m^2)	0.591	0.018	0.000	(0.556—0.627)	22.68	0.155	68.88%	46.64%	0.003	—	0.178	0.223
Waist circumference (cm)	0.610	0.017	0.000	(0.576—0.645)	78.00	0.197	71.90%	47.81%	0.006	0.178	—	0.001
Women	0.635	0.021	0.000	(0.593—0.676)	78.00	0.260	68.42%	57.59%	—	—	—	—
Men	0.542	0.032	0.174	(0.480—0.605)	87.80	0.132	43.44%	69.76%	—	—	—	—
WHR (cm/cm)	0.567	0.018	0.000	(0.539—0.594)	0.86	0.123	61.63%	50.64%	0.000	0.223	0.001	—
Women	0.587	0.022	0.000	(0.544—0.630)	0.82	0.154	77.03%	38.39%	—	—	—	—
Men	0.524	0.032	0.433	(0.463—0.586)	0.89	0.098	57.38%	52.41%	—	—	—	—
IFG+IGT												
WHtR (cm/cm)	0.713	0.022	0.000	(0.670—0.755)	0.53	0.351	62.33%	72.79%	—	0.106	0.556	0.026
BMI (kg/m^2)	0.685	0.022	0.000	(0.642—0.729)	23.38	0.316	77.40%	54.22%	0.106	—	0.254	0.492
Waist circumference (cm)	0.706	0.021	0.000	(0.665—0.748)	79.80	0.351	82.88%	52.19%	0.556	0.254	—	0.032
Women	0.732	0.026	0.000	(0.682—0.783)	79.80	0.420	79.57%	62.38%	—	—	—	—
Men	0.656	0.039	0.000	(0.579—0.733)	90.30	0.242	43.40%	80.76%	—	—	—	—

For peer review only

WHR (cm/cm)	0.667	0.022	0.000	(0.638—0.695)	0.87	0.274	69.18%	58.23%	0.026	0.492	0.032	—
Women	0.686	0.027	0.000	(0.633—0.739)	0.83	0.312	86.02%	45.20%	—	—	—	—
Men	0.631	0.038	0.003	(0.556—0.705)	0.92	0.261	54.72%	71.38%	—	—	—	—
NDDM												
WHtR (cm/cm)	0.730	0.017	0.000	(0.696—0.764)	0.52	0.366	74.21%	62.43%	—	0.000	0.001	0.010
BMI (kg/m^2)	0.677	0.020	0.000	(0.639—0.716)	24.32	0.315	64.68%	66.81%	0.000	—	0.093	0.596
Waist circumference (cm)	0.700	0.018	0.000	(0.665—0.735)	78.00	0.292	81.35%	47.81%	0.001	0.093	—	0.429
Women	0.714	0.021	0.000	(0.673—0.756)	77.10	0.344	81.76%	52.63%	—	—	—	—
Men	0.686	0.033	0.000	(0.622—0.750)	88.00	0.298	56.99%	72.85%	—	—	—	—
WHR (cm/cm)	0.688	0.018	0.000	(0.661—0.715)	0.88	0.304	67.73%	62.71%	0.010	0.596	0.429	—
Women	0.696	0.022	0.000	(0.653—0.738)	0.84	0.301	79.75%	50.31%	—	—	—	—
Men	0.681	0.030	0.000	(0.622—0.740)	0.92	0.299	60.22%	69.66%	—	—	—	—

ROC, receiver operating characteristic; IFG, impaired fasting glucose; IGT, impaired glucose tolerance; IFG+IGT, IFG combined with IGT; NDDM, newly-diagnosed diabetes mellitus; AUC, area under curve; SE, standard error; CI, confidence interval; WHtR, waist-to-height ratio; BMI, body mass index; WHR,

For peer review only

waist-to-hip ratio.

For peer review only

Table 4. Multivariate analysis of baseline anthropometric indices with respect to risk of subsequent pre-diabetes and NDDM

	Isolated IFG			Isolated IGT			IFG+IGT			NDDM		
	HR	95% CI	*P*	HR	95% CI	*P*	HR	95% CI	*P*	HR	95% CI	*P*
WHtR (cm/cm)	1.471	(0.901—2.402)	0.123	1.951	(1.550—2.457)	0.000	3.002	(2.137—4.216)	0.000	2.765	(2.065—3.703)	0.000
BMI (kg/m^2)	1.186	(0.699—2.012)	0.526	1.571	(1.241—1.988)	0.000	3.298	(2.224—4.892)	0.000	2.305	(1.773—2.998)	0.000
Waist circumference (cm)	1.603	(1.112—2.310)	0.011	1.644	(1.275—2.118)	0.000	4.570	(2.948—7.084)	0.000	2.666	(1.886—3.769)	0.000
WHR (cm/cm)	1.182	(0.739—1.889)	0.486	0.972	(0.724—1.304)	0.848	1.571	(1.003—2.465)	0.048	1.706	(1.196—2.433)	0.003

NDDM, newly-diagnosed diabetes mellitus; IFG, impaired fasting glucose; IGT, impaired glucose tolerance; IFG+IGT, IFG combined with IGT; HR, hazard ratio; CI, confidence interval; WHtR, waist-to-height ratio; BMI, body mass index; WHR, waist-to-hip ratio.

Cox proportional hazards models were used to calculate HR and 95% CI. A univariate analysis was performed for each potential risk factor firstly, including age (years), gender (male/female), systolic blood pressure (mmHg), diastolic blood pressure (mmHg), fasting plasma glucose (mmol/L), 2 hour plasma glucose (mmol/L) (after oral glucose tolerance test), HbA1c (%), total cholesterol (mmol/L), triglyceride (mmol/L), high-density lipoprotein cholesterol (mmol/L), low-density lipoprotein cholesterol (mmol/L), diabetes family history (yes/no), current smoking status (yes/no), physical activity situation (yes/no), WHtR (low/high), BMI (low/high), WC (low/high) and WHR (low/high). The four anthropometric indicators were dichotomized into low or high level by using cut-off values derived from previous ROC curve analysis. Then those risk factors with a *P*-value < 0.2 in univariate analysis were selected to enter the multivariate model.

For peer review only

Figure legends

Figure 1. ROC curves of baseline anthropometric indices in subjects who developed (A) isolated IFG, (B) isolated IGT, (C) IFG+IGT and (D) NDDM. ROC, receiver operating characteristic; IFG, impaired fasting glucose; IGT, impaired glucose tolerance; IFG+IGT, IFG combined with IGT; NDDM, newly-diagnosed diabetes mellitus; WHtR, waist-to-height ratio; BMI, body mass index; WHR, waist-to-hip ratio; WC, waist circumference.

Supplemental Figure 1. Flow-chart of study design. NGT, normal glycemic tolerance; OGTT, oral glucose tolerance test; NDDM, newly-diagnosed diabetes mellitus. The re-visited participants in the blue background came from the baseline populations; the subjects in the pink background were the ones recruited in this study, who were normoglycemic at baseline.

For peer review only

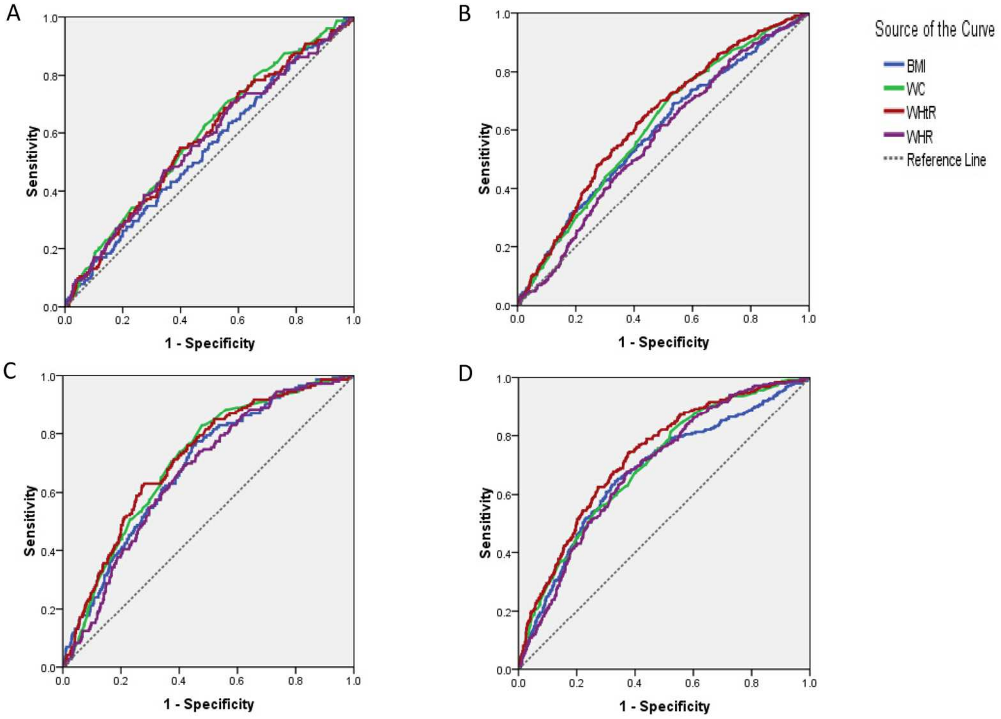

Figure 1. ROC curves of baseline anthropometric indices in subjects who developed (A) isolated IFG, (B) isolated IGT, (C) IFG+IGT and (D) NDDM. ROC, receiver operating characteristic; IFG, impaired fasting glucose; IGT, impaired glucose tolerance; IFG+IGT, IFG combined with IGT; NDDM, newly-diagnosed diabetes mellitus; WHtR, waist-to-height ratio; BMI, body mass index; WHR, waist-to-hip ratio; WC, waist circumference.

158x114mm (300 x 300 DPI)

only

Supplemental Table 1. Baseline characteristics of all the participants screened in Luzhou and Wenjiang

	Luzhou baseline survey			*P*	Wenjiang baseline survey			*P*
	Total (n = 1582)	Men (n = 495)	Women (n = 1087)		Total (n = 303)	Men (n = 154)	Women (n = 149)	
Age (year)	57 (50—63)	60 (54—66)	56 (49—61)	0.000	47 (43—54)	47 (43—54)	46 (42—52)	0.161
Female (N/n%)	1087 (68.71%)	—	—		149 (49.17%)	—	—	
Height (cm)	157.00 (152.40—163.00)	165.00 (160.50—169.00)	154.55 (151.00—158.20)	0.180	161.28 ±7.60	166.46 ±5.27	155.56 ±5.32	0.000
Weight (kg)	59.00 (53.00—65.30)	65.00 (58.30—72.00)	56.50 (51.20—62.50)	0.000	61.88 ±11.36	67.66 ±8.84	55.49 ±10.38	0.000
Hip circumference (cm)	94.00 (89.20—99.00)	95.00 (90.00—100.00)	94.00 (89.00—98.20)	0.655	93.47 ±6.04	95.01 ±5.43	91.76 ±6.24	0.000
SBP (mmHg)	120.67 (108.67—135.67)	126.00 (113.83—140.17)	119.00 (107.00—133.37)	0.000	114.90 ±14.27	118.20 ±14.02	111.26 ±13.69	0.000
DBP (mmHg)	75.33 (69.00—82.67)	79.00 (71.67—88.17)	74.00 (68.00—80.67)	0.000	78.40 ±16.26	81.15 ±10.84	75.36 ±20.27	0.001
FPG (mmol/L)	5.14 (4.93—5.34)	5.15 (4.96—5.38)	5.13 (4.92—5.32)	0.011	4.90 (4.60—5.10)	4.90 (4.70—5.20)	4.80 (4.60—5.10)	0.286
2hPG (mmol/L)	6.32 (5.57—7.00)	6.32 (5.57—6.98)	6.32 (5.57—7.01)	0.777	6.00 (5.00—6.70)	5.90 (5.03—5.78)	6.00 (5.00—6.80)	0.541
HbA1c (%)	5.70 (5.40—5.90)	5.70 (5.50—5.95)	5.70 (5.40—5.90)	0.069	5.48 ±0.42	5.51 ±0.38	5.45 ±0.45	0.228
TG (mmol/L)	1.33 ±0.94	1.29 ±0.84	1.34 ±0.98	0.388	1.10 (0.80—1.80)	1.50 (0.90—2.18)	0.90 (0.70—1.50)	0.000
TC (mmol/L)	4.44 (3.75—5.18)	4.32 (3.63—5.12)	4.50 (3.82—5.19)	0.017	4.53 ±0.83	4.60 ±0.82	4.45 ±0.83	0.178
HDL-c (mmol/L)	1.28 (1.06—1.52)	1.26 (1.03—1.52)	1.29 (1.08—1.52)	0.107	1.59 ±0.39	1.47 ±0.32	1.71 ±0.41	0.000
LDL-c (mmol/L)	2.52 ±0.77	2.47 ±0.74	2.54 ±0.79	0.078	2.86 ±0.75	2.92 ±0.70	2.79 ±0.80	0.252
WHtR (cm/cm)	0.52 (0.48—0.56)	0.53 (0.49—0.56)	0.52 (0.48—0.56)	0.900	0.49 (0.45—0.53)	0.50 (0.47—0.53)	0.47 (0.44—0.51)	0.000
BMI (kg/m^2)	23.74 (21.61—26.00)	24.04 (21.81—26.14)	23.68 (21.51—25.92)	0.462	23.49 (21.64—25.59)	24.40 (22.33—26.03)	22.44 (21.10—24.24)	0.000
Waist circumference (cm)	82.00 (76.00—89.00)	87.00 (80.00—92.45)	80.00 (75.00—87.10)	0.201	79.00 (72.00—86.00)	84.00 (79.00—89.00)	73.00 (69.75—78.00)	0.000
WHR (cm/cm)	0.86 (0.80—0.91)	0.89 (0.83—0.94)	0.85 (0.79—0.90)	0.476	0.86 (0.80—0.91)	0.83 (0.78—0.89)	0.87 (0.81—0.92)	0.256
Outcomes at follow-up: N/total (%)								
NGT	757 (47.85%)	203 (41.01%)	554 (50.97%)	—	215 (70.96%)	103 (66.88%)	112 (75.17%)	—
Isolated IFG	131 (8.28%)	51 (10.30%)	80 (7.34%)	—	28 (9.24%)	12 (7.79%)	16 (10.74%)	—
Isolated IGT	304 (19.22%)	103 (20.81%)	201 (18.49%)	—	38 (12.54%)	24 (15.58%)	14 (9.40%)	—
IFG+IGT	137 (8.66%)	46 (9.29%）	91 (8.37%)	—	15 (4.95%)	11 (7.14%)	4 (2.68%)	—

For peer review only

NDDM	253 (15.99%)	92 (18.59%)	161 (14.81%)	—	7 (2.31%)	4 (2.61%)	3 (2.01%)	—

SBP, systolic blood pressure; DBP, diastolic blood pressure; FPG, fasting plasma glucose; 2hPG, 2 hour plasma glucose (after oral glucose tolerance test); TG, triglyceride; TC, total cholesterol; HDL-c, high-density lipoprotein cholesterol; LDL-c, low-density lipoprotein cholesterol; WHtR, waist-to-height ratio; BMI, body mass index; WHR, waist-to-hip ratio; NGT, normal glucose tolerance; IFG, impaired fasting glucose; IGT, impaired glucose tolerance; IFG+IGT, IFG combined with IGT; NDDM, newly-diagnosed diabetes mellitus.

Data are expressed as mean ± SD, or median (interquartile range), or N (%).

Mann-Whitney U analysis was used for DBP and BMI in Luzhou, TG and HDL-c in Wenjiang; one-way ANOVA analysis was used for the rest measurements in two surveys.

P value of men versus women.

For peer review only

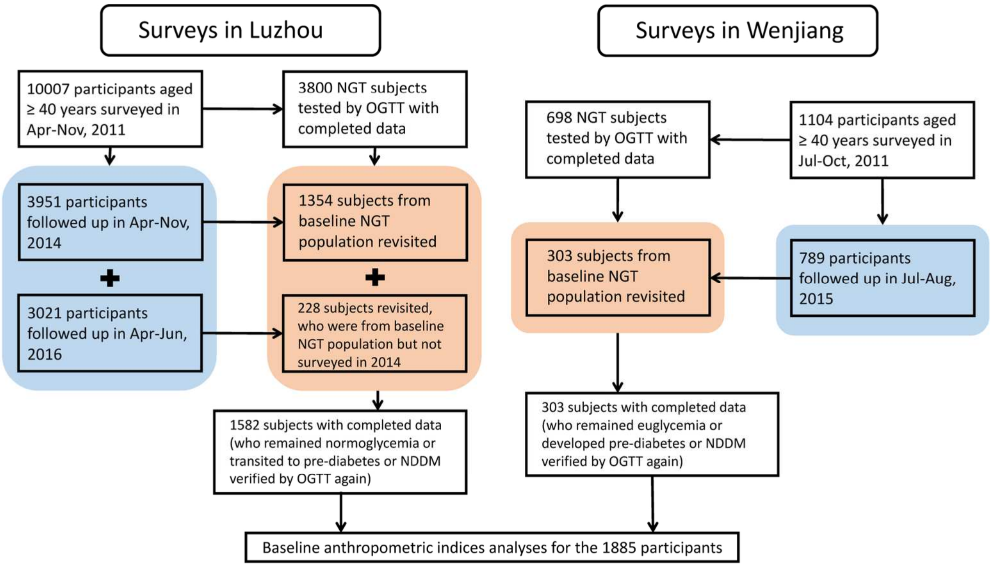

Supplemental Figure 1. Flow-chart of study design. NGT, normal glycemic tolerance; OGTT, oral glucose tolerance test; NDDM, newly-diagnosed diabetes mellitus. The re-visited participants in the blue background came from the baseline populations; the subjects in the pink background were the ones recruited in this study, who were normoglycemic at baseline.

165x94mm (300 x 300 DPI)

view only

STROBE 2007 (v4) checklist of items to be included in reports of observational studies in epidemiology*
Checklist for cohort, case-control, and cross-sectional studies (combined)

Section/Topic	Item #	Recommendation	Reported on page #
**Title and abstract**	1	(*a*) Indicate the study's design with a commonly used term in the title or the abstract	1, 2
		(*b*) Provide in the abstract an informative and balanced summary of what was done and what was found	2
**Introduction**			
Background/rationale	2	Explain the scientific background and rationale for the investigation being reported	4
Objectives	3	State specific objectives, including any pre-specified hypotheses	4
**Methods**			
Study design	4	Present key elements of study design early in the paper	4, 5
Setting	5	Describe the setting, locations, and relevant dates, including periods of recruitment, exposure, follow-up, and data collection	4. 5
Participants	6	(*a*) *Cohort study*—Give the eligibility criteria, and the sources and methods of selection of participants. Describe methods of follow-up<br>*Case-control study*—Give the eligibility criteria, and the sources and methods of case ascertainment and control selection. Give the rationale for the choice of cases and controls<br>*Cross-sectional study*—Give the eligibility criteria, and the sources and methods of selection of participants	4, 5
		(*b*) *Cohort study*—For matched studies, give matching criteria and number of exposed and unexposed<br>*Case-control study*—For matched studies, give matching criteria and the number of controls per case	NA
Variables	7	Clearly define all outcomes, exposures, predictors, potential confounders, and effect modifiers. Give diagnostic criteria, if applicable	5, 6
Data sources/ measurement	8*	For each variable of interest, give sources of data and details of methods of assessment (measurement). Describe comparability of assessment methods if there is more than one group	*5, 6*
Bias	9	Describe any efforts to address potential sources of bias	NA
Study size	10	Explain how the study size was arrived at	4, 5
Quantitative variables	11	Explain how quantitative variables were handled in the analyses. If applicable, describe which groupings were chosen and why	5, 6
Statistical methods	12	(*a*) Describe all statistical methods, including those used to control for confounding	6
		(*b*) Describe any methods used to examine subgroups and interactions	6
		(*c*) Explain how missing data were addressed	6
		(*d*) *Cohort study*—If applicable, explain how loss to follow-up was addressed<br>*Case-control study*—If applicable, explain how matching of cases and controls was addressed	6

For peer review only

		Cross-sectional study—If applicable, describe analytical methods taking account of sampling strategy	
		(*e*) Describe any sensitivity analyses	NA
**Results**			
Participants	13*	(a) Report numbers of individuals at each stage of study—eg numbers potentially eligible, examined for eligibility, confirmed eligible, included in the study, completing follow-up, and analysed	7
		(b) Give reasons for non-participation at each stage	5
		(c) Consider use of a flow diagram	5
Descriptive data	14*	(a) Give characteristics of study participants (eg demographic, clinical, social) and information on exposures and potential confounders	6
		(b) Indicate number of participants with missing data for each variable of interest	NA
		(c) *Cohort study*—Summarise follow-up time (eg, average and total amount)	5
Outcome data	15*	*Cohort study*—Report numbers of outcome events or summary measures over time	6
		Case-control study—Report numbers in each exposure category, or summary measures of exposure	NA
		Cross-sectional study—Report numbers of outcome events or summary measures	NA
Main results	16	(*a*) Give unadjusted estimates and, if applicable, confounder-adjusted estimates and their precision (eg, 95% confidence interval). Make clear which confounders were adjusted for and why they were included	7, 8
		(*b*) Report category boundaries when continuous variables were categorized	6-8
		(*c*) If relevant, consider translating estimates of relative risk into absolute risk for a meaningful time period	7, 8
Other analyses	17	Report other analyses done—eg analyses of subgroups and interactions, and sensitivity analyses	NA
**Discussion**			
Key results	18	Summarise key results with reference to study objectives	8
Limitations	19	Discuss limitations of the study, taking into account sources of potential bias or imprecision. Discuss both direction and magnitude of any potential bias	9, 10
Interpretation	20	Give a cautious overall interpretation of results considering objectives, limitations, multiplicity of analyses, results from similar studies, and other relevant evidence	8-10
Generalisability	21	Discuss the generalisability (external validity) of the study results	10
**Other information**			
Funding	22	Give the source of funding and the role of the funders for the present study and, if applicable, for the original study on which the present article is based	10

*Give information separately for cases and controls in case-control studies and, if applicable, for exposed and unexposed groups in cohort and cross-sectional studies.
Note: An Explanation and Elaboration article discusses each checklist item and gives methodological background and published examples of transparent reporting. The STROBE checklist is best used in conjunction with this article (freely available on the Web sites of PLoS Medicine at http://www.plosmedicine.org/, Annals of Internal Medicine at http://www.annals.org/, and Epidemiology at http://www.epidem.com/). Information on the STROBE Initiative is available at www.strobe-statement.org.

For peer review only
